# The Bermuda Triangle: The Pragmatics, Policies, and Principles for Data Sharing in the History of the Human Genome Project

**DOI:** 10.1007/s10739-018-9538-7

**Published:** 2018-12

**Authors:** Kathryn Maxson Jones, Rachel A. Ankeny, Robert Cook-Deegan

**Affiliations:** 1 Department of History, Princeton University, Princeton, NJ, USA; 2 MBL McDonnell Foundation Scholar, Marine Biological Laboratory, Woods Hole, MA, USA; 3 School of Humanities, The University of Adelaide, Adelaide, Australia; 4 School for the Future of Innovation in Society, Consortium for Science, Policy & Outcomes, Arizona State University, Barrett & O’Connor Washington Center, Washington, D.C., USA

**Keywords:** Bayh-Dole Act, Bermuda Principles, Big science, Bioinformatics, Biotechnology, *C. elegans*, Celera Genomics, Co-production, Community resource projects, Data hoarding, Data release, Data sharing, Databases, DNA Databank of Japan (DDBJ), Department of Energy (DOE), DNA sequencing, Ethical Legal and Social Implications (ELSI), European Bioinformatics Institute (EBI), GenBank, Gene patenting, Genetics, Genetic mapping, Genome commons, Genomics, Human Genome Project (HGP), Intellectual property, Medical genetics, Model organisms, Molecular biology, Moral economy of science, National Center for Human Genome Research (NCHGR), National Human Genome Research Institute (NHGRI), National Institutes of Health (NIH), Nematode worm, Open science, Patents, Physical mapping, Post-genomics, Public domain, Reference sequence, Science policy, Wellcome Trust

## Abstract

The Bermuda Principles for DNA sequence data sharing are an enduring legacy of the Human Genome Project (HGP). They were adopted by the HGP at a strategy meeting in Bermuda in February of 1996 and implemented in formal policies by early 1998, mandating daily release of HGP-funded DNA sequences into the public domain. The idea of daily sharing, we argue, emanated directly from strategies for large, goal-directed molecular biology projects first tested within the “community” of *C. elegans* researchers, and were introduced and defended for the HGP by the nematode biologists John Sulston and Robert Waterston. In the *C. elegans* community, and subsequently in the HGP, daily sharing served the pragmatic goals of quality control and project coordination. Yet in the HGP human genome, we also argue, the Bermuda Principles addressed concerns about gene patents impeding scientific advancement, and were aspirational and flexible in implementation and justification. They endured as an archetype for how rapid data sharing could be realized and rationalized, and permitted adaptation to the needs of various scientific communities. Yet in addition to the support of Sulston and Waterston, their adoption also depended on the clout of administrators at the US National Institutes of Health (NIH) and the UK nonprofit charity the Wellcome Trust, which together funded 90% of the HGP human sequencing effort. The other nations wishing to remain in the HGP consortium had to accommodate to the Bermuda Principles, requiring exceptions from incompatible existing or pending data access policies for publicly funded research in Germany, Japan, and France. We begin this story in 1963, with the biologist Sydney Brenner’s proposal for a nematode research program at the Laboratory of Molecular Biology (LMB) at the University of Cambridge. We continue through 2003, with the completion of the HGP human reference genome, and conclude with observations about policy and the historiography of molecular biology.

## Introduction

The daily flow of new genomic sequence information into the public domain, generated by a global network of laboratories, became one of the signature features of the Human Genome Project (HGP). The HGP was the nonprofit and publicly funded effort that generated the first genomic reference sequences for *Homo sapiens* and five model organisms between 1990 and 2003 and helped to refine many key genomics technologies.^1^ The HGP culminated in a draft human sequence completed in 2001 (Lander et al.). A more refined human reference genome was published in pieces through 2004 (International Human Genome Sequencing Consortium), with the model organism sequences all made publicly available by 2002 (Goffeau et al. 1996; Blattner et al. 1997; The *C. elegans* Sequencing Consortium 1998; Adams et al. 2000; Mouse Genome Sequencing Consortium 2002).^2^ This deluge of DNA sequence data, to which scientists and the general public had immediate access, stood in stark contrast both to the conventional sharing of data at the time of journal publication and to the restrictions that corporate sequencers were placing on their data.^3^ The HGP investigators from the six participant nations—the US, the UK, Germany, France, Japan, and China—gave all of their HGP-funded DNA sequences away, online and for free, presumably within 24 h of generating them (1996 Wellcome Trust Press Release; Lander et al. 2001, pp. 860, 876). Francis Collins, the director of the US National Institutes of Health (NIH) since 2009 and a predominant leader in the HGP, noted in a folksy song he penned to commemorate the project that, “they worked without resting, and gave it away” (Jasanoff 2005, p. 234).

The “Bermuda Principles,” as they came to be known by their authors and followers, were the first official recommendations that the HGP-funded DNA sequences be deposited daily into the public domain.^4^ Drafted and ratified as the official HGP-wide data-sharing policies at the 1996 International Strategy Meeting for Human Genome Sequencing at the Hamilton Princess Hotel in Bermuda, which was sponsored by the British nonprofit philanthropic funder the Wellcome Trust, the Bermuda Principles stood at the core of the strategy that the HGP’s leaders crafted to decipher the six target genomes rapidly, to high quality, and with minimal restrictions on data access (1996 NIH Bermuda Meeting Report; 1996 Wellcome Trust Bermuda Meeting Report; 1996 Wellcome Trust Press Release).^5^ The Principles were applied first to HGP human genomic sequences and, throughout 1998, to the remainder of the model organisms included in the HGP: yeast (*Saccharomyces cerevisiae*), bacteria (*Escherichia coli*), nematode worm (*Caenorhabditis elegans*), fruit fly (*Drosophila melanogaster*), and mouse (*Mus musculus*) (Guyer 1998). They have featured in popular, journalistic, scientific, policy, and legal literature ever since, a symbol of science conducted with public funds, and for the public good (Marshall 1996 2001; Waterston and Sulston 1998; Sulston and The International Human Genome Sequencing Consortium 2001; Sulston and Ferry 2002, Chaps. 4–5; Waterston et al. 2002; Collins et al. 2003b; Powledge 2003; Edwards 2008; Church and Hillier 2009; Contreras 2010, 2011, 2014, 2015, 2016, 2017; Cook-Deegan and Heaney 2010; Jasny 2013; Sherkow and Greely 2015; Rai and Sherkow 2016; Reardon et al. 2016; Cook-Deegan et al. 2017).

Since 1996, and especially since the “completion” of the HGP in 2003, similar practices and project-wide policies have also become much more common, and have sometimes even been mandated by funders. Funding agency policies, however, frequently diverge from those adopted by the international, consortium-style projects that now populate much of genomics, as well as many other twenty-first century biological research fields (Holden 2002; The ENCODE Project Consortium 2004). Francis Collins and other leaders from the Human Genome Project have often lauded the HGP’s pre-publication sharing precedent, promoting this practice as a touchstone of modern, “data-centric” biology alongside the adoption of compatible policies by funders (Collins 1998; Sulston and The International Human Genome Sequencing Consortium 2001; Collins et al. 2003a, b; Contreras 2015; Green et al. 2015).^6^ Since the conclusion of the HGP, the NIH in particular has continued to write rapid data-sharing mandates into its grant requirements, especially in genomics, with additional public and private funders and large communities of researchers, such as in proteomics, following suit with their own sets of policies, recommendations, and requirements (Salzberg et al. 2003; Cottingham 2008; Piwowar et al. 2008; Rodriguez 2008; Toronto International Data Release Workshop 2009; Field et al. 2009; Kaye et al. 2009; Rodriguez et al. 2009; Knoppers et al. 2011; Piwowar 2011; Davies et al. 2013; Leonelli 2013b, 2016; Knoppers 2014; Contreras 2015; Levin and Leonelli 2016).

Humanists, social scientists, and policy analysts have responded accordingly, exploring the practical and ethical challenges of sharing biological research data, and sometimes materials, with unprecedented rapidity. Major difficulties include data curation and maintenance; medical privacy and discrimination; widely divergent understandings of openness; the compatibility of policies and legal structures across projects, fields, and nations; the hegemony of the journal as a medium for releasing research results; and the construction of databases and brick- and-mortar institutions for sharing datasets, dynamic models, reagents and other materials, and even model organism strains (McCain 1991, 1995; Leonelli 2013a; Levin and Leonelli 2016; Reardon et al. 2016; Cook-Deegan et al. 2017; Deverka et al. 2017; Hilgartner 2017; Reardon 2017). An additional complicating factor is that the relationships between sharing and commercialization, two processes often perceived to be in tension with one another, are in fact quite knotty (Leonelli 2010; Williams 2013; Parthasarathy 2017). Our purpose in this paper is to explore how, in an endeavor as visible, expensive, and widespread as the HGP, such a radical policy for sharing molecular biological research data—the daily, online release of HGP-funded DNA sequences—arose and endured, both inside and outside of the project.^7^

## Mapping the Way Forward

Several lines of scholarship have addressed these questions, regarding the origins and motivations for the daily release of sequence data during the HGP. In some ways, as the historian Bruno Strasser has argued, such rapid data sharing was an outgrowth of strategies inspired by the persistence of natural historical practices, namely collection and comparison, in the work of the molecular biologists who made early use of the US DNA sequence databank, called GenBank, in the 1980s (Strasser 2005, 2006, 2008, 2009, 2011; also, Cook-Deegan 1994, Chap. 18; Stemerding and Hilgartner 1998). By promoting analyses requiring massive data inputs, gleaned from new media such as databanks but also older reference works of data collections (of protein sequences and structures, for example), these biologists engaged in data-sharing practices that challenged, and sometimes conflicted with, the more traditional model of sharing research results through journal articles (and thus at the time of publication). Another inspiration, detailed by historian Hallam Stevens, was the countercultural ethos of the Free and Open Source Software (FOSS) movement, which was concentrated at the Massachusetts Institute of Technology (MIT) and Stanford University by the early 1960s. The scientists associated with the FOSS movement, Stevens argues, buoyed a collaborative, decentralized, and non-proprietary style of work, which was ultimately imported into molecular biology and then the HGP and reflected in the project’s data-sharing policies (Stevens 2011, 2013, esp. Chap. 5, 2015a, c; on FOSS, also Kelty 2008; Tozzi 2017).

Sheila Jasanoff and Stephen Hilgartner have interpreted the situation sociologically, utilizing the science and technology studies (STS) framework of “interactional co-production” pioneered by Jasanoff and developed with San-Hyun Kim (Jasanoff 2003, 2004, 2005, 2011; Jasanoff and Kim 2009, 2015).^8^ In their STS interpretation, pre-publication data sharing emerged in the HGP largely through embeddedness in a “constitutional” system, wherein scientific and social order were co-produced and the HGP’s data-sharing policies were conditioned, if not fully determined, by “imaginaries,” or widely held and deeply institutionalized visions of what science and technology can achieve if properly governed (Hilgartner 1995b, 2004a, b, 2012b, 2013, 2017; Jasanoff 2003, 2005). Science policies, public understandings, scientific publications and pronouncements, the media, and culture all help constitute these imaginaries; and one imaginary intimately linked to rapid data sharing was that of a genome sequence “commons,” generated by the HGP, residing in the public domain, and unencumbered by patent claims.

A related line of inquiry by legal and policy analysts has built on broader theoretical frameworks of the commons, especially the genome commons, and has explored its nature and function in biology from the late twentieth century to the present (Ostrom 1990, 2010; Hardin 1998; Eisenberg 2000, 2006; Eisenberg and Nelson 2002; Cook-Deegan and Dedeurwaerdere 2006; Cook-Deegan 2007; Hess and Ostrom 2007; Boyle 2010; Contreras 2010, 2011, 2014, 2015, 2016, 2017; Wiechers et al. 2013; Cook-Deegan et al. 2017, esp. pp. 399–401; Deverka et al. 2017; Parthasarathy 2017; Strandburg et al. 2017). The central problem for these authors has been how data in the public domain, which anyone can read and use, creates public goods, whether through unfettered basic research making use of the data, intellectual property protections, commercialization, or other means. The HGP created a “topsy-turvy” world in which the pre-publication sharing of genomic sequences promoted both rapid follow-on research and some instances of patenting and commercialization, while simultaneously undercutting other such instances (Cook-Deegan and Heaney 2010, p. 402). These legal and policy studies have intersected with yet further lines of anthropological and STS scholarship, interrogating how the HGP’s joint goals—of a public-domain human genome and economic growth—have sometimes come into conflict, creating novel challenges for ethics, governance, healthcare delivery, and identity politics (Helmreich 2000; Jasanoff 2004, 2005, 2011; Reardon 2005, 2017; Rajan 2006; Helmreich and Roosth 2010; Leonelli 2010; Hilgartner 2017; Roosth 2017). While valuable for exploring the consequences of rapid data sharing and the associated policies in both public and private institutions, however, such work has generally not interrogated the historical causes of rapid sharing, leaving this question open for further study.

Stephen Hilgartner has argued that a new “governing frame” materialized as fledgling genome scientists struggled with the exigencies of communication and the territorial politics of smaller-scale, mostly bench-based molecular biology around 1990, precipitating the adoption of new data-sharing policies (Hilgartner 1998, 2004a, 2013, 2017, esp. Chaps. 3–4).^9^ The necessity of rapid sharing to monitor the HGP’s progress and quality became apparent under these uncharted circumstances, giving rise to what Hilgartner calls the “‘when’ question” regarding the mandated release of HGP-funded data (Hilgartner 2017, Chap. 6, esp. p. 167). Heavy-handed control via policy by the NIH, the US Department of Energy (DOE), and later the UK Wellcome Trust was written into the HGP from the beginning (Hilgartner 2004a, p. 124, 2013, pp. 404–406, 2017), for instance, especially through prescriptive (though often revised) 5-year policy plans (Collins and Galas 1993; Collins 1995, 1998). Deliberate efforts by the HGP’s leadership resulted in policies for the rapid sharing of genetic, physical, and genomic maps, the precursors to sequencing in the HGP, by 1992 (NIH, DOE Guidelines Encourage Sharing of Data, Resources 1993; Hilgartner 1998).^10^ Whether they were ready for journal publication or not, according to these 1992 policies, all of the HGP’s maps and early sequences funded by the NIH or the DOE were to be deposited into the public biomolecular databases within 6 months of their generation (NIH, DOE Guidelines Encourage Sharing of Data, Resources 1993). Yet in this instance, as Hallam Stevens has demonstrated, while the imperatives of new kinds of biological projects indeed engendered the need for rapid data sharing, scientists and administrators in the HGP were nevertheless relying on technological infrastructures, biomolecular databases, already in place, thereby raising the question of how these technologies were already influencing sharing practices in the life sciences (Stevens 2017).

Sociologists, philosophers, and historians, alongside several prominent scientists, have validated how in the last quarter century, these networked databases have reconfigured scientific data itself. They have challenged traditional divisions of public and private, enabling the rapid electronic sharing of various data outputs amongst collaborators, journals, and the general public and helping to transform genomics into a global enterprise (Burks 1989; Smith 1990; McCain 1991, 1995; Cinkosky et al. 1992; Hilgartner and Brandt-Rauf 1994; Hilgartner 1995b, 1997, 1998, 2017; Burks and Wood 2003; Leonelli 2010, 2012, 2013a, b, 2016; Davies et al. 2013; Ankeny and Leonelli 2015; Levin and Leonelli 2016; Stevens 2017). One crucial impetus for databases in the life sciences, alongside the persistence of natural historical practices and the FOSS movement, was the rise of model organism “communities,” such as those centered on fruit fly, yeast, mouse, and the nematode worm.^11^ The work practices and related moral economies in these communities promoted frequent communication and the rapid sharing of data, materials, and methods among collaborators, as well as with geographically dispersed colleagues working on the same model organisms (Kohler 1994; Ankeny 1997, 2000, 2001, 2003, 2007; Creager 2001; Rader 2004; Nelson 2017).

The creators of the maps and early sequences for these organisms adopted cutting-edge advances in both molecular biology and computing, becoming some of the earliest contributors to, and users of, databases for genetics and genomics (Leonelli 2011; Kelty 2008, 2012; Leonelli and Ankeny 2012; November 2012; Dietrich et al. 2014; García-Sancho 2015). To provide an infrastructure for this rising tide of DNA sequences, moreover, by the 1980s the “International Nucleotide Sequence Database Collaboration” had emerged amongst GenBank at the US NIH, the Nucleotide Archive of the European Molecular Biology Laboratory (EMBL) in Heidelberg, and the DNA Databank of Japan (DDBJ) in Tokyo (Cook-Deegan 1994, Chap. 18, esp. pp. 288–291; Stevens 2017). Through an arrangement not without its own political complexities (Stevens 2017), these three databases shared data daily, rapidly producing mirror images of the DNA sequences then available in the public domain. In the decade prior, the American community of protein crystallographers had also established the Protein Data Bank (PDB) at the Brookhaven National Laboratory in New York, enabling pre-publication sharing of intricate structural X-ray coordinates (Berol 2001; Berman et al. 2004). By the early 1990s, in sum, a diverse array of avenues for rapidly sharing biological research data, often well before journal publication, had emerged, all of which were both technologically feasible and professionally acceptable.

When the HGP’s scientists turned from mapping to sequencing the human genome in 1996, quality control and project coordination remained central issues. Echoing a similar, earlier argument by the legal scholar Jorge Contreras (2011), Hilgartner has recently maintained that by refining the former NIH-DOE policies, from 1992, and mandating the daily sharing of the HGP’s DNA sequences in the public databases, the Bermuda Principles served both of these goals while also reframing the HGP’s obligations to databases and journals (Hilgartner 2017, esp. Chaps. 6, 8). Because traditional publication alone was too slow to permit project coordination, the Bermuda Principles called for unprecedentedly rapid (daily) release of the HGP’s sequences (first in the human and later in the five model species) to GenBank, EMBL, and the DDBJ (Ouellette and Boguski 1997; Stevens 2017). The move multiplied what Hilgartner calls “UJAD data,” which was “unpublished in journals and available in databases” (Hilgartner 2017, esp. pp. 175–185, 229); and because UJAD data was so new to biologists in such high volumes, this move, in turn, required new rules for governance and control.

Initially limiting the applicability of the Bermuda Principles to the HGP did ease their adoption within this circumscribed project, but the Principles still rewrote collective norms, or “knowledge-control regimes,” of how data, albeit of new kinds and volumes, would be shared in biomolecular databases and journal publications (Hilgartner 2017, Chaps. 6, 8). One important way in which the Bermuda policies remained a part of the HGP was via periodic clarification about authorship: from 1998 through 2003, a series of revisions to the Bermuda Principles stipulated how sequence users could share the HGP’s data while still remaining fair to the data’s generators, usually distinct groups of people, who often planned analytical, follow-up journal publications after initial data release (Hilgartner 2017, pp. 172–184). Journal articles were the hallmark currency of scientific credit, and remain so today; modifying the Bermuda Principles to conform to this standard promoted their survival in the Human Genome Project and beyond.

Two crucial questions, however, remain unanswered in the literature. Why *daily* sharing? And how was this controversial policy ratified and, in several cases, extended well beyond the HGP? It was no small task, and certainly an improbable one: getting agreement from hundreds of scientists, with their politically sensitive public funders, to converge on a single data-sharing policy. Thanks to the World Wide Web and the Internet, HGP scientists could feasibly share data daily by the mid-1990s; as Hallam Stevens has indicated, at least in privileged institutions, the technical capability was indeed present (Stevens 2017). Yet technology alone does not explain why the HGP’s leadership chose such an iconoclastic policy over other options. Neither does Hilgartner’s account (Hilgartner 2017), which, despite its explicit attention to the “‘when’ question” (p. 167), notes that even amongst the staunchest supporters of daily data sharing, surprise surrounded its putative adoption in the HGP at the 1996 International Strategy Meeting for Human Genome Sequencing in Bermuda (2017, esp. p. 173 and Chap. 6, n. 69).^12^

Other authors have implied that daily data release was uncontroversial within the HGP, and that its execution was simple (McElheny 2010, p. 118; Nielsen 2011, p. 7). In both the US and abroad, however, several leading HGP scientists and administrators had legitimate concerns about flouting publication norms, adversely affecting data quality and downstream research by premature sharing, and infringing upon national and agency-level policies already in place to promote patenting and commercialization (Unterhuber 1997; Balter 1998; Eisenberg 2000; Cook-Deegan and Heaney 2010; Contreras 2011; Jasny 2013). In this paper, we explore more deeply how disagreements among the HGP’s scientists and administrators were resolved, and we address how this same group of individuals overcame the blatant incompatibility of the Bermuda Principles with major commercialization policies and interests in the US, Germany, Japan, and France. We build on our own shorter communications (Reardon et al. 2016; Cook-Deegan et al. 2017), which address these open issues, and add considerable detail and nuance to the existing interpretations of how daily data sharing became embedded in the HGP after 1996.

It is our central claim that daily sharing arose in the HGP most directly based on the strategies for completing large, goal-directed molecular biology projects first tested within the “community of *C. elegans* researchers” founded by Sydney Brenner (Wood and the community of *C. elegans* researchers 1988). While often referenced by historians and other analysts, some of whom have explored the history of *C. elegans* biology in depth (Ankeny 1997, 2000, 2001, 2003, 2007; Wilson 1999; Horvitz and Sulston 2000; Sulston and the International Human Genome Sequencing Consortium 2001, p. 28; Sulston and Ferry 2002; Ankeny and Leonelli 2015; Stevens 2015a, c; Leonelli 2016; Reardon et al. 2016; Cook-Deegan et al. 2017; Hilgartner 2017, pp. 165–175, esp. chpa. 6, n. 69), this connection—between the nematode worm and the human genome—has yet to be fully documented. Sharing data well before publication was one of several tactics that emerged amongst early *C. elegans* cellular, genetic, and physical mappers for quality control, progress assessment, and coordination in the 1970s and 1980s. This sharing strategy extended to preliminary worm sequencing from 1990 through 1994, when the leading nematode biologists John Sulston and Robert Waterston also took up human sequencing. Other model organism biologists, such as those working on yeast and mouse, also engaged in pre-publication data sharing; but by 1995, thanks to computing advances, *daily* data sharing was both possible and encouraged for nematode *and* human DNA sequences produced by scientists within the *C. elegans* community. John Sulston and Robert Waterston endorsed this practice for all HGP human sequences in 1996, and the pragmatic rationales, quality control and project coordination, still remained from the earlier decades of nematode biology. By 1998, the year in which the Bermuda Principles were extended to all of the HGP’s non-human model organisms (Guyer 1998), the project’s major funders in the US, the UK, Germany, France, and Japan had at least ostensibly allowed their HGP scientists to abide by daily data sharing. China followed suit in 1999, its entry to the HGP contingent upon compliance with the Bermuda Principles (Qiang 2004).

In addition to documenting their *C. elegans* origins, we argue that the Bermuda Principles endured as a cornerstone of the HGP because their central tenet, daily data sharing, was partly aspirational. The daily-sharing standard was only sometimes, if ever, required to reach the goals for which it was supposedly employed, so it persisted partly as an archetype, while the Bermuda Principles, as policies, remained flexible in their implementation and justification. The metrics of data quality and completeness for sequences to be released at various timescales—and the technologies enabling such release—were in flux before and during the HGP, and remain so today. Even for *C. elegans*, technical factors resulted in evolving data-sharing practices before 1995; and when daily sharing did become technologically feasible in certain places, even the HGP centers most ardently committed to it (namely, Sulston and Waterston’s laboratories in Cambridge, England, and Washington University in St. Louis, Missouri) still sometimes fell short. Following the 1996 International Strategy Meeting for Human Genome Sequencing in Bermuda, the daily-sharing standard remained but a rhetorical aspiration in many other places. Some center leaders never even attempted to abide by it, trying to release data as rapidly as possible but treating daily sharing as unrealistic or unwise, despite the HGP’s explicit policy calling for it. Practices also diverged for model species after 1998, particularly in the HGP’s laboratories located outside the US and Great Britain (Goffeau et al. 1996; Blattner et al. 1997; The *C. elegans* Sequencing Consortium 1998; Adams et al. 2000; Mouse Genome Sequencing Consortium 2002). Daily data sharing grew more feasible for human sequencing as the HGP narrowed from a larger set of pilot centers to the five largest and most technologically advanced centers, nicknamed the “G5,” in 1999 (Hilgartner 2017, pp. 209–210).

The policy translation of the Bermuda Principles proved similarly piecemeal. Although daily sharing moved from the *C. elegans* moral economy to explicit HGP policies, covering first the human genome and then all five of what were, by that time, the project’s model organisms, the Bermuda Principles became part of agency policies only within the HGP divisions of the US NIH and the UK-based Wellcome Trust.^13^ All of the government funders for human sequencing, including those in the US, Germany, Japan, and France, had to finesse, or even sidestep, existing or pending science policies encouraging patenting or restricted data release to incorporate daily sharing into their projects. In some cases, these ostensibly incompatible policies gave national corporations privileged and limited pre-publication access to data. Tacit understandings about the Bermuda Principles usually prevailed, allowing scientists to partake in the international HGP while also keeping national and agency policies for the commercialization of molecular biology intact. The aspirational nature of the Bermuda Principles, in these instances, made them flexible; this same plasticity was essential for the Principles to become enduring policies in the HGP and beyond, despite not being fully honored always and everywhere.^14^

The arguments justifying daily data release were likewise malleable, shifting with the stakeholders and stakes and alongside various scientific and political realities. After 1996, the pragmatic Bermuda standards for HGP data quality and completeness continued to be adapted to advances in sequencing and computing. But equally importantly, as daily release was redeployed in human genomics from the nematode testing ground, the commercial potential of molecular biology became ever more entwined with the fate of the policy. Human genomics, in contrast to worm genomics, falls in the upper-right-hand corner of “Pasteur’s Quadrant,” harboring applied relevance in both medicine and industry while also contributing to basic knowledge (Stokes 1997; Cook-Deegan and Heaney 2010, p. 391). The private and public rhetoric, utilized by the HGP’s leadership in relation to daily data sharing, expanded in tandem, reflecting the new stakes of this practice for intellectual property. Arguments in favor of daily sharing appealed to the commons, wherein the daily release of the HGP’s sequences promoted both unfettered research use and some patents while also precluding broad patents that might hinder future research.^15^ The Bermuda Principles, for example, effectively prevented patents on genes of unknown biological functions, by funneling sequences daily into the public domain as prior art (Cook-Deegan 1994; Eisenberg 1996, 2000; Burris et al. 1998; Eisenberg and Nelson 2002). Yet, at least in the US, they hardly endangered patents on genes of understood organic significance and clear commercial value, such as on insulin or growth hormone, and in fact promoted such patenting as a part of the economic justification for the HGP (Williams 2013). In some of the debates about daily data release, especially at the three International Strategy Meetings for Human Genome Sequencing in Bermuda from 1996 to 1998, these nuances—including the fact that the HGP’s leadership was not entirely opposed to commercialization—were clear. Yet in other instances, including in press releases and public pronouncements in the later phases of the Human Genome Project, this point was largely lost.

The Bermuda Principles’ flexibility, in both implementation and justification, surfaced perhaps most clearly in 1998. J. Craig Venter, a scientist formerly at the NIH, announced that a new company, eventually named Celera Genomics, would “race” the public HGP to complete the human genome, selling restrictive database subscriptions, releasing data quarterly, and patenting genes and other technologies in turn (Private-Sector Sequencing Planned 1998; Shreeve 2004; Williams 2013). Upon the commencement of this race, the HGP’s skeptics claimed that the job could be done faster and more accurately by industry, using Venter’s strategy, which differed from that of the HGP both technically and in its data-sharing policies (Hilgartner 2017, Chap. 7). Yet as a defense of the HGP, and even as a proposal, the Bermuda Principles accentuated the contrasts between the public project and the proprietary database on offer by Celera. This defense merged with the public works mission that had characterized the HGP from the start, because, consistent with the goals of the Bermuda Principles, the financial support for the HGP was based on creating openly available, high-quality resources (Cook-Deegan 1994; Hilgartner 2017). When the HGP’s survival was threatened in 1998, supporters of the public effort could point to its data being utilized by scientists around the world, before publications or reference sequences were completed, and to the fact that Celera’s promise of quarterly data sharing was hardly binding. The HGP survived: a tie with Celera was brokered in 2000 (Office of the White House Press Secretary 2000); the dueling “draft” sequence papers from the public and private efforts were published in *Nature* and *Science* in 2001 (Lander et al.; Venter et al.); and the “completion” of the HGP was hailed in 2003 (International Human Genome Sequencing Consortium). These accomplishments, in turn, served as grounds for the Wellcome Trust and the NIH to extend the Bermuda Principles to other genomics and large-scale “community resource projects” in biology (Collins et al. 2003b; *Sharing Data from Large-Scale Biological Research Projects*; Toronto International Data Release Workshop 2009). This success story, however, created some misconceptions, obscuring the full history of the Bermuda Principles such as their pragmatic foundations from *C. elegans* and the nuanced stance they represented towards the genome commons and commercialization.

Today, the Bermuda Principles represent an enduring legacy of the HGP, and, according to the goals and the metrics of those who supported them, have been wildly successful. Yet these outcomes, as with any outcomes resulting from deeply human affairs, were far from inevitable. At the outset of the HGP, it was hardly obvious that the data-sharing policies to be developed for human genomics would draw so heavily on practices from model organisms, let alone from the tiny nematode worm *C. elegans*. It was perhaps at least as likely, moreover, that the large community of human geneticists, and their more restrictive data-sharing norms, would have informed these basic policy structures for the HGP (Cook-Deegan 1994). The history of how the worm won out, especially over human genetics, was complex, contingent, and, when viewed from the distance of nearly a quarter-century, at times downright surprising: a tale of powerful personalities, high-stakes science, realpolitik, and perhaps unlikely collaborations. This history commenced, or at least our story does, in 1963, when the biologist Sydney Brenner began his research in England on *C. elegans*, launching the formation of a community in which daily data sharing germinated, took root, and later grew in the HGP. We trace this story to 2003, when the human genome sequence was nearing its celebrated completion and several key follow-on projects were already underway.

## Data Sharing in *C. elegans* Mapping and Sequencing (1965–1995)

At the Laboratory of Molecular Biology (LMB) at the University of Cambridge in England, within the Division of Molecular Genetics led by Francis Crick of DNA-structure fame, Sydney Brenner spent the latter half of the 1960s building a research program on the physiology and genetics of *C. elegans*. In 1963, he wrote to the LMB chairman and crystallographer Max Perutz that he wanted to expand his research program from bacteria and the viruses that infect them (bacteriophages) to include multicellular animals (metazoans) (Ankeny 1997, p. 49; 2003; de Chadarevian 1998, p. 85; 2002, p. 286). Brenner’s original idea was to study *C. briggsae* (Ankeny 2001, p. 475), but by 1965, he had settled on *C. elegans* because it was “small, rapidly growing, and easily handled in the laboratory” (Brenner 2009, p. 413). In a tradition dating back to Thomas Hunt Morgan and his fruit flies at Columbia University in New York City, Brenner focused on building genetic maps of mutations for behavioral aberrations, locating the relative positions of genetic markers, or identifiable DNA sequences, in the worm genome based on the frequencies with which they were (or were not) inherited together or with other markers (Allen 1978; Kohler 1994; Gaudillière and Rheinberger 2004). As the nematode project took off, bolstered by the postwar growth in molecular biology in Britain, Brenner joined Crick as a Division Head within the LMB (de Chadarevian 2002, Chap. 9).

By the late 1960s and early 1970s, Brenner was inviting numerous researchers to visit his laboratory (Ankeny 1997, Chap. 3, esp. pp. 99–100; de Chadarevian 1998, pp. 100–102). The publications arising from the worm project were limited in the early years, and visiting the laboratory was the main way to find out about progress, learn new techniques, and obtain worm strains and other materials required for research. John Sulston joined the LMB as a staff scientist in 1969, after having completed his postdoctoral fellowship at the Salk Institute in La Jolla, California with Leslie Orgel (Ankeny 1997, p. 99). Orgel introduced Sulston to Crick, who introduced him to Brenner (Ferry 2009). Upon his arrival in Cambridge, Sulston joined a group of researchers who were all focused on the molecular biology of *C. elegans*, from its genetics to its biochemical and cellular environments through cell lineage maps and behavioral phenotypes (Ankeny 1997; de Chadarevian 1998). While Sulston did not contribute substantially to the early genetic mapping, by 1974 Brenner and others amongst his LMB colleagues had described over 300 behavioral mutants with suspected genetic causes and had mapped over 100 *C. elegans* genes (Brenner 1973, 1974a, b). In 1974, Sulston and Brenner together described the DNA content of the worm (Sulston and Brenner 1974).

The laboratory prioritized complete physiological understandings and long-term outputs, achieved via techniques that the historical science writer Horace Freeland Judson described as “brute-force” (1996, p. 613).^16^ The general spirit of the LMB was collaborative, as Brenner and the other laboratory members openly shared ideas; and some participants recall individual members, including those more junior, being permitted great independence in deciding which projects to pursue, and how, even if these projects did not always result in successes (Waterston to KMJ 2017). Junior researchers and technicians, who might have been treated subordinately elsewhere, often authored or co-authored major publications (Sulston 1976; Sulston and Horvitz 1977; Sulston et al. 1983), receiving only loose guidance from Brenner (Waterston to KMJ 2017). This style echoed that of Frederick Sanger, the LMB head of Protein Chemistry, who at the time was developing one of the first methods for DNA sequencing (Sanger and Coulson 1975; Sanger et al. 1977; García-Sancho 2006, 2015; Chow-White and García-Sancho 2012).^17^ Sanger promoted what John Sulston recalled in 2011 as a “flat structure” in his laboratory, such that especially in social spaces, such as coffee klatches, members were “encouraged to gather” (Waterston and Sulston interview 2011). Sulston, alongside several historians and even Sanger himself, recalled an infrastructural element in Sanger’s work, with the goal of creating tools for other biologists to use (Sanger 1988; Brownlee 2014; García-Sancho 2015, esp. Chaps. 1–2). “The whole purpose” of this “Sanger style,” Sulston also recollected, “was to make sequencing faster, faster, faster. First protein and then nucleic acid” (Waterston and Sulston interview 2011).

Throughout the 1970s, the LMB also played host to numerous other researchers and students who later established their own laboratories for worm biology. These collaborators included Waterston, who after a meeting with Brenner at the 1969 Physiology course at the Marine Biological Laboratory (MBL) in Woods Hole, Massachusetts became a postdoctoral fellow in 1972 to work on muscle mutants (Ankeny 1997, pp. 100–101, n. 22). Another was H. Robert Horvitz, who joined in 1974 to work on nerve cell lineages after completing his doctoral work on mutations in T4 bacteriophage (Ankeny 1997, p. 120; 2003, p. 88). These acolytes maintained and promoted the values that they encountered at the LMB, but also developed a distributed work structure that was based, at least initially, on the literal subdivision of *C. elegans* experiments by the worm’s anatomical systems (Pickvance 1976; Wood and the community of *C. elegans* researchers 1988; de Chadarevian 1998, p. 93). Community members complemented this division of labor by sharing data, techniques, and materials as rapidly amongst collaborators as possible (Ankeny 1997, Chap. 3, 2003; de Chadarevian 2004, pp. 102–104). Worm research generally failed to appear rapidly in articles because many projects were larger in scale and took considerable time, and the LMB’s funding structures in particular usually did not require prompt journal publications. More informal sharing mechanisms thus helped to maintain the coherence of this geographically diffuse yet intellectually unified group, in principle to avoid costly research duplications (Waterston and Sulston interview 2011).

Such sharing took on various forms and utilized evolving technologies. It first occurred, for instance, in the context of biannual workshops and via quarterly newsletters (most notably, the *Worm Breeder’s Gazette*), and later through worm-strain sharing centers (e.g., first at the University of Minnesota, then at the University Missouri) and electronic databases (e.g., A *C. elegans DataBase*, or *ACeDB*, and its successor, Wormbase) (de Chadarevian 1998; Kelty 2008, 2012; Stevens 2015c, p. 473; Waterston to KMJ 2017).^18^ There was some dissent: not everyone in the worm community equally endorsed these values. Some members also did duplicative work in the early years, and the need to provide tangible results—especially for those completing their dissertations, securing postdoctoral positions, or trying to earn tenure in conventional university settings—at times created tensions with those at the LMB who did not need to publish results rapidly (Ankeny 1997, pp. 81–82, n. 1). Despite these conflicts, however, the nematode community was robust and growing by the late 1980s. Around 100 worm laboratories populated the US, the UK, Europe, and Japan, and pre-publication data sharing was becoming a key community value (de Chadarevian 2004, p. 100). By the 1990s, the tally of worm laboratories had reached about 150, with the highest concentration in the US and the UK.

Sulston remained a conspicuous leader. In the early 1970s, the research on *C. elegans* suggested that a fixed cell lineage generated the entire complement of cells in embryogenesis, implying no further divisions after hatching (Ankeny 1997, Chap. 3; Waterston to KMJ 2017). Despite this, however, Sulston found dividing cells in the larval ventral nerve cord. Using Nomarski microscope optics that allowed him to visualize individual cells, Sulston began tracing nervous system development in embryonic *C. elegans* (Horvitz and Sulston 2000).^19^ With colleagues at the LMB including Robert Horvitz, Sulston helped establish the full developmental cell lineage of this system in the worm (Sulston 1976; Sulston and Horvitz 1977; Sulston et al. 1983). The work was lauded as groundbreaking both inside and well beyond the worm community, and it formed the basis of Sulston’s share of the 2002 Nobel Prize in Physiology or Medicine, which he won alongside Sydney Brenner and Horvitz.

Sulston resolved in 1983 to build a physical map of the worm genome (García-Sancho 2007). Following his “brute-force” tactics in the embryonic cell lineage, this project was “the obvious next step … as infrastructure for an entire community” (Eddy interview 2012). Unlike the low-resolution genetic map, however, which only established the relative locations of genetic markers, this “contig” or physical map would locate DNA markers in their exact chromosomal addresses (Bostanci 2004; de Chadarevian 2004; Hilgartner 2017, Chap. 2, esp. Figures 2.7–2.8). “Contig” maps were built through meticulous analyses of “clones,” massively replicated pieces of DNA generated by recombinant DNA (rDNA) methodologies (Hughes 2001, 2011; Yi 2008, 2015).^20^ These methods spliced random fragments of genomic DNA into bacteriophage or other molecular vectors (cosmids) inserted into the genomes of bacterial hosts (Marshall 1995, p. 784), which replicated normally to amplify the target DNA for chemical analyses. Clones containing overlapping fragments were recognized, then tiled together to form “contigs,” digital constructs reflecting overlaps of “contiguous” DNA sequences. Genetic maps, in contrast, were built by placing mutated genes, traditionally recognized by phenotypic (organism-level) disruptions, in their relative orders on chromosomes (Allen 1978; Kohler 1994; Gaudillière and Rheinberger 2004; Hilgartner 2017, Chap. 2). The standard measure of distance for physical maps was lengths of DNA, or numbers of base pairs, but for genetic maps, it reflected the frequency of co-inheriting markers during cell division.

The construction of the physical contig map was best completed by a central laboratory, or a small number of laboratories, capable of generating large amounts of data. The correlation of clones with genes on the genetic map, however, was most easily pursued on a distributed basis, “by continuous collaboration with the whole worm community” (Sulston and the International Human Genome Sequencing Consortium 2001, p. 28; also, Waterston to KMJ 2017). Integrating the physical and genetic maps would produce a “hybrid,” or true “genomic” map, which would remove some of the limitations of genetic and contig maps on their own and eventually make large-scale genome sequencing easier (Olson et al. 1989, p. 1434; Doggett 1992; Waterston to KMJ 2018).^21^ Yet such integration called for fastidiously coordinated analyses of data, alongside the systematic sharing of clones. Sulston instituted the sharing of mapping data with the community as rapidly as possible, using evolving digital networks such as BITNET (de Chadarevian 2004, p. 99 and Figure 5.2; also, García-Sancho 2015, pp. 123–126). But BITNET, founded in 1981 as the “Because It’s There NETwork” or “Because It’s Time NETwork,” was slow, and unable to handle large amounts of data (Stevens 2015a, esp. p. 855; Waterston to KMJ 2018). Sulston often had to wait a month or more to share progress on the physical map with the worm community, sending copies on magnetic tape to sites in the US and elsewhere, and keeping duplicates for himself to back up the work (Sulston and Ferry 2002, p. 55; de Chadarevian 2004, pp. 98–99; Stevens 2015c, p. 477; Waterston to KMJ 2018). The early worm mappers and cloners, in other words, progressed imperfectly and in fits and starts. But their sharing practices, first interpersonal and then paper-based, and soon implanted into the wobbly-yet-improving computing infrastructure, were pragmatic. Coordination between the LMB and Robert Waterston’s team, by that point based at Washington University in St. Louis, on the physical map was crucial, a link that solidified from 1985 to 1986 when Waterston spent a sabbatical year in Cambridge (Roberts 1990, p. 1312; Ankeny 1997, p. 100; Wilson 1999, p. 52).

Not long after Waterston returned stateside, however, a breakthrough changed how physical mapping was done. Until that time, nematode biologists had been relying on bacterial cosmids to amplify target DNA (Coulson et al. 1986; Monaco and Larin 1994). Yet a group of geneticists at Washington University, which included Maynard Olson, soon developed Yeast Artificial Chromosomes (YACs), which could insert much larger fragments of DNA into hosts before replicating and thus generate different biases from those introduced by the bacterial cosmids used to that point (Cook-Deegan 1989, p. 662; Olson et al. 1986; Burke et al. 1987; Carbon 1993). The sizes of the cloned DNA pieces, and different representations of target sequences, could aid in closing pesky gaps between contigs, enabling completion of the worm physical map by the late 1980s (Coulson et al. 1988, 1991, 1995; Sulston et al. 1992; Sulston and the International Human Genome Sequencing Consortium 2001, p. 28). Seeing the echoes of Brenner and Sanger’s emphases on infrastructure building in his own style, Sulston labeled himself jauntily in 2011 as “not a proper scientist.” By this, he meant that he was “not problem-oriented really. I’m a mapper and always have been” (Waterston and Sulston interview 2011). Empowered by their successes in managing large-scale projects in the nematode worm, including the new physical map, Sulston the “improper” scientist, alongside Robert Waterston, would soon assume leading roles in the HGP.

Meanwhile, Michael Morgan, the director of Research Projects and Ventures at the Wellcome Trust in London, was receiving a rapidly increasing number of grant applications in genetics (Giles 2010). Morgan had arrived at the Wellcome Trust in 1983, initially with the title of program manager for Molecular and Cellular Biology (Morgan and Wallace interview 2012). By the mid-1980s, under Morgan’s leadership and on the advice of the medical geneticist David Weatherall, the Trust had established a “Genetics Interest Group,” the purpose of which was to begin reviewing these grants more competently. The group included the Nobelist James Watson, who became the founding director of the NIH Office and, by 1989, the National Center for Human Genome Research (NCHGR), in October of 1988 (Watson 1990, p. 46). From these positions of heavy administrative clout, and complemented by his enormous scientific authority, Watson promoted Anglo-American genomics collaborations for the remainder of his career, including those already emerging between the Sulston and Waterston *C. elegans* groups around the nematode physical map (see below; also, Cook-Deegan 1994, esp. pp. 333–336).

**Fig. 1:**
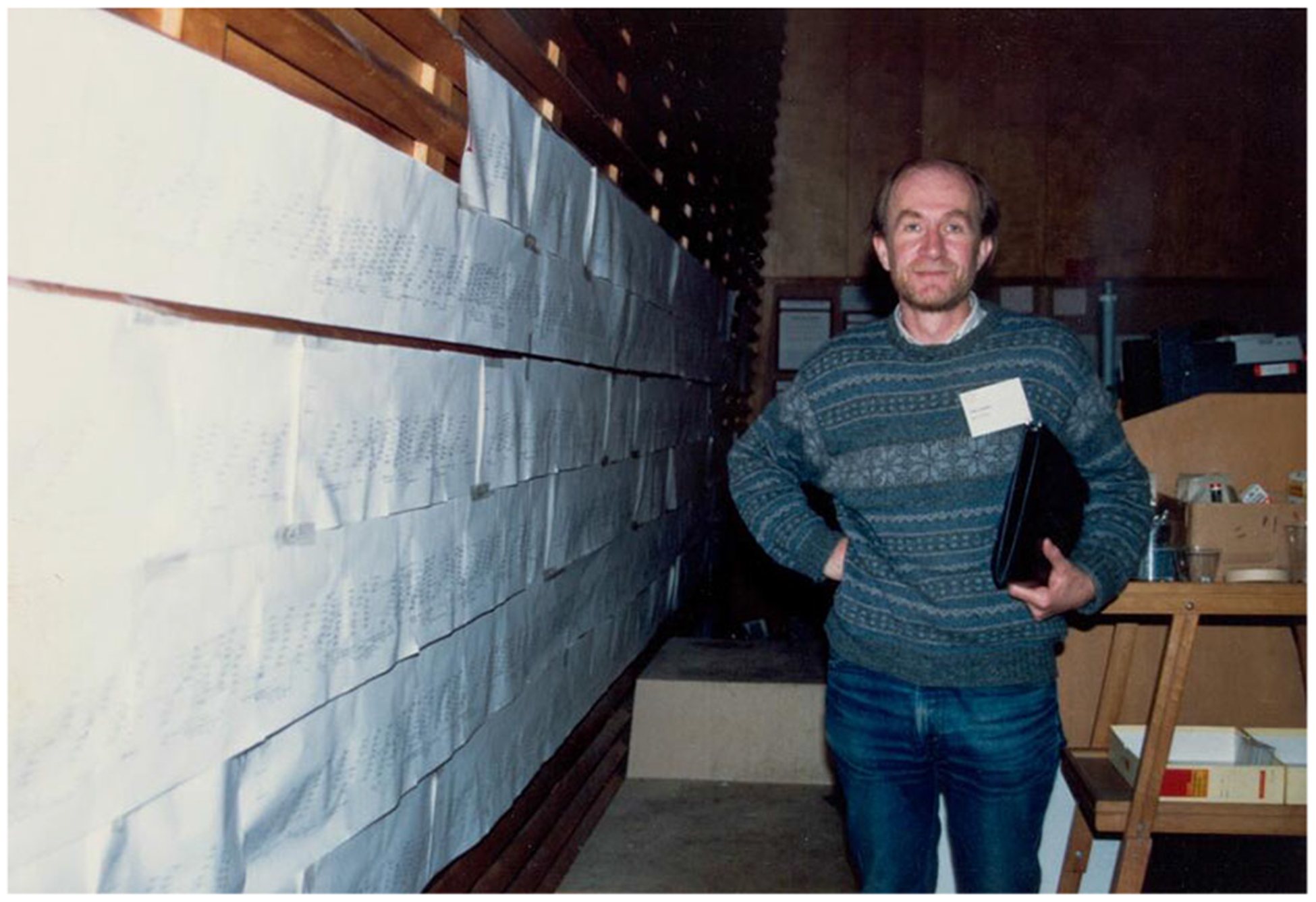
Alan Coulson standing beside the physical map of the worm genome, nearly completed and displayed at the 1989 International *C. elegans* Meeting at CSHL. This material has been provided by the Wellcome Library where the originals may be consulted. Reproduced with Alan Coulson’s permission. “Photographs of CSHL Meeting, May 1989,” Alan Coulson Papers, PPCOU/B/1/11, License: CC-BY-NC. https://wellcomelibrary.org/item/b20268634#?m=0&cv=0&c=0&s=0&z=−0.0755%2C-0.0254%2C1.0001%2C0.8925

A second event involving Watson, data sharing, and the worm took place at the Cold Spring Harbor Laboratory (CSHL) on Long Island. CSHL had been home to Max Delbrück’s phage group after the Second World War (Summers 1993; Creager 2009, 2010), and in the late 1980s played an integral role in the origins of the HGP. The laboratory had just hosted the 1986 Molecular Biology of *Homo sapiens* symposium, at which Walter Gilbert of Harvard famously slapped a $3 billion anticipated price tag on the human genome sequence, an audacious goal at a cost that afflicted nearly all in attendance with sticker shock (Cook-Deegan 1994, pp. 109–116). CSHL also hosted the International *C. elegans* Meeting in 1989, where Robert Waterston, John Sulston, and Alan Coulson displayed a nearly finished version of the nematode’s physical map (Sulston and Ferry 2002, pp. 66–68; de Chadarevian 2004, p. 103). This map was printed on successive pieces of paper and taped to a wall, after a 1988 meeting in Virginia had prompted the decision that the HGP would employ a “map-then-sequence” strategy—already underway in the nematode—for tackling the human genome (Watson 1990, p. 46; de Chadarevian 2004, pp. 102–104 and Figure 5.4; also, Fig. 1, below). Privileging genetic and physical maps before beginning sequencing in earnest, this strategy was also ongoing in *E. coli* and yeast, with the preliminary plans for a genome project in Drosophila underway too (Watson 1990, p. 48). Yet when Watson saw the worm physical map, so cogently displayed in CSHL with “virtually all of the genome … available as cloned sets of overlapping DNA fragments,” he decided that the human project should adopt the nematode community’s methods for mapping and sequencing, complete with their unique data-sharing norms (Watson 1990, p. 48; Sulston and the International Human Genome Sequencing Consortium 2001, p. 28; Waterston to KMJ 2017).^22^ According to Waterston’s recollections, Watson’s verdict likely represented the first significant input from administrators on either side of the Atlantic in the fledgling HGP, at least with regards to data release (Waterston to KMJ 2017). Watson’s input, moreover, helped to coalesce the worm community around a full-scale genome-sequencing project, to be continued after the mapping phases were completed.^23^

By 1990, the nematode community was transitioning from mapping to sequencing. In 1992, funded by the British Medical Research Council (MRC) and the US NIH, Waterston and Sulston scaled up their preliminary sequencing efforts, building on existing genetic and physical maps (Sulston et al. 1992, funding information on 41; Waterston and Sulston interview 2011; Waterston to authors Jan 2017). Success in the nematode led to early human sequencing, and in October 1992 the pair proposed in an extension of their original NIH grant, as Waterston noted: “1) to complete the rest of the worm sequence; 2) to contribute 3 Mb [three megabases (Mb), or three million base pairs] to complete the yeast sequence; and 3) to initiate human sequencing on the PKD [polycystic kidney disease] region and on gene rich regions of chromosome 7 in order to test our methods on human genomic DNA” (Waterston to authors Jan 2017). This foray into the human (and also into yeast), which was “an extension of the partnership … formed for the worm,” was approved, and the renewed grant, with a new number from the NIH but the same title, “*C. elegans* sequencing,” ran from 1993 through 1998 with Waterston as the Principal Investigator (Project Information, 1P01HG000956–01 1993; Waterston to authors Feb 2017).

Progress in both the worm and the human genomes continued apace. By August 1993, Sulston and Waterston had sequenced over 2 Mb of contiguous nematode DNA, enabling the discovery of new genes and functional elements (Wilson 1999, p. 52; Wilson et al. 1994). It was becoming clear, however, that large-scale human sequencing was going to require considerably larger facilities; and in response, in 1993, the Wellcome Trust christened the Sanger Centre near the University of Cambridge in Hinxton (García-Sancho 2015, esp. Chap. 1). The attribution was a clear tribute to Frederick Sanger’s contributions to biology: not just the DNA sequencing method that had earned him a share of the Nobel Prize in 1980, but also his self-deprecating and highly collegial style of doing science. Sulston became the Sanger Centre’s first director, and through his team the Wellcome Trust became the primary patron of human genome sequencing in the UK (Cook-Deegan 1994, pp. 334–335; 1996 Wellcome Trust Press Release; García-Sancho 2015). Next to the Sanger Centre, Waterston’s St. Louis center produced a somewhat smaller overall volume of sequence from then on, but the two laboratories in England and Missouri were still “sharing fully in dealing with the new problems that human sequence brought” (Waterston to authors Feb 2017). The Waterston-Sulston partnership also continued its nematode work, and in August 1994 a meeting between the Sanger Centre and Waterston’s laboratory personnel produced a 5-year plan to finish the worm genome (Wilson 1999, p. 53). The worm sequence became publicly available by 1998, entering the public domain via GenBank (The *C. elegans* Sequencing Consortium 1998).

Genomic sequencing at the Sanger and St. Louis centers was always an assembly-line endeavor, aided by the rapid sharing of materials and mapping and sequencing data to prevent misassemblies (for a general description of these strategies, see Bostanci 2004, esp. pp. 163–165). In St. Louis, the NIH funded the worm, yeast, and early human sequencing. At the Sanger Centre, the MRC funded the worm, while the Wellcome Trust supported yeast and human sequencing with the aid of supplemental NIH backing.^24^ “The Wellcome Trust’s funding to the Sanger Centre” was from late 1995, and remained through the remainder of the HGP, “the largest commitment to a single centre for human genomic sequencing in the world” (1996 Wellcome Trust Press Release), with the Centre also leading the worldwide distribution of *C. elegans* clones (de Chadarevian 2004, p. 106). The data-sharing efforts in the worm and human genomes, moreover, relied on evolving technologies, including databases and other electronic resources. *ACeDB*, which was similar to other genome databases and browsers then in development, used hypertext to help discern the relative relationships amongst clones and contigs (Bostanci 2004, pp. 164–165; de Chadarevian 2004, pp. 106–107; Stevens 2015c, p. 480); and the Worm Community System (WCS), which was an early hyper-library, incorporated map and sequence data from *ACeDB*, the *Worm Breeder’s Gazette*, and other print and digital sources into a hub for collaborators.^25^

The exact timing of sequence sharing and the recipient audiences, however, varied with sequence quality. In 1992, the NIH and the DOE instituted their policies mandating the release of HGP-funded mapping and early sequence data to the public repositories within 6 months (NIH, DOE Guidelines Encourage Sharing of Data, Resources 1993). All sequences were to be sent to GenBank, and human genetic maps (with their correlative disease data) to the Genome Database (GDB) at Johns Hopkins University in Baltimore (Brandt 1993; Fashman et al. 1997; Letovsky et al. 1998).^26^ Technically, the 6-month disclosure rule applied to all the data generated under Waterston and Sulston’s NIH grant funds. But the nematode community’s sharing norms were much more rapid than this, as demonstrated by the duo’s strategies for all organisms from 1992 and quoted from Waterston’s personal files:

Our purpose is to make … sequence data available to the scientific community as quickly as possible. Therefore, data will be released to the public databases immediately upon completion. We will define completion as an annotated contiguous double stranded sequence. (Waterston to authors Jan 2017)

Although Waterston’s records “lack notes on some of the crucial decisions” about the evolution of his and Sulston’s sharing practices from this point forward (Waterston to authors Feb 2017), we have utilized grant applications and other sources to reconstruct the process.

Sulston and Waterston’s 1992 statement referred obliquely to a crucial division between “finished” (or “completed”) and “unfinished” data. Ideally, finished sequences were to contain minimal errors (e.g., incorrectly named bases or places where base repeats were erroneously identified), no gaps, and annotations flagging matches between sequences and known genes or other functional elements. There was, as of yet, no common standard for maximum error rate in finished sequence, but unfinished data—essentially, contigs assembled by computers when the clones went through the early sequencing machines—contained more errors. The laboratory of the computational biologist Phil Green, first at Washington University in St. Louis and later at the University of Washington in Seattle, developed the predominant algorithms and software for aiding in clone alignment and the burdensome human labor of finishing, the major bottleneck in completing any genome (Brownlee 2004, p. 13992; Hilgartner 2017, Chap. 2). Green’s *phrap* (or “Phil’s revised assembly program”) handled clone alignment; and *phred* (“Phil’s revised editor”), developed in its early phases with the bioinformaticist LaDeana Hillier and first tested on worm clones sequenced by Waterston in St. Louis and Leroy Hood, Lee Rowen, and Maynard Olson in Seattle, assigned quality scores to bases, outperforming the software installed on the sequencing machines from the then-predominant commercial manufacturer, Applied Biosystems (ABI) (Ewing and Green 1998; Ewing et al. 1998; Brownlee 2004, p. 13992).^27^ David Gordon and Chris Abajian, in Green’s laboratory at the University of Washington, developed *Consed* (“consensus editor”)-*Autofinish*, a graphical tool for visualizing and editing contigs during finishing (Gordon et al. 1998, 2001; Brownlee 2004, p. 13992). *Consed* was later revised for next-generation sequencing, the technologies developed after Frederick Sanger’s methodology was fully automated (Mardis 2008; Shendure and Ji 2008; Gordon and Green 2013; Curnutte et al. 2014, 2016; García-Sancho 2015, esp. Chaps. 5–6). All three programs remain in use today (Phred, Phrap, Consed).

Through 1993, unfinished sequences were simply too unreliable for Sulston and Waterston to share, either with collaborators or more publicly. They released their finished, or “completed,” *C. elegans* and human sequences to GenBank, also sharing the nematode data with their community through *ACeDB* and the *Worm Breeder’s Gazette*, but they did not want colleagues building on unreliable or low-quality data (Waterston to authors Jan 2017; Feb 2017). Despite holding back their unfinished sequences, however, release of finished data was rapid, even for sequences that were too difficult to complete in a timely manner. These recalcitrant data were annotated as such and released to the public domain as quickly as possible, with the explicit intent “clear that we will submit the sequence as soon as it is ready, not waiting for publication or imposing any other delay” (Waterston to authors Jan 2017). Almost always, this type of release occurred before the NIH-DOE 6-month requirement.

The situation shifted again between 1994 and 1995, when the programs *phred* and *phrap* were improved and adapted for human sequencing (Waterston to authors Feb 2017). After the 1994 paper announcing the completion of 2.2 continuous Mb of nematode DNA (Wilson et al.; de Chadarevian 2004, p. 104), Waterston and Sulston began feeling more confident about their unfinished sequence assemblies. They published a list of cosmids, cloned pieces of DNA that had been sequenced, in the *Worm Breeder’s Gazette*, noting those with error problems and those matching the sequences of known genes (The *C. elegans* Genome Consortium and Sanger Centre 1994, p. 14; Waterston to authors Feb 2017). One noteworthy part of this communication was that Sulston and Waterston were reporting on data of divergent quality. “Cambridge has included only finished cosmids and those cosmids which are contiguous but still have one or more problem areas,” the newsletter stated; but “St. Louis has also included cosmids which have one or two gaps but which are otherwise in good shape” (The *C. elegans* Genome Consortium and Sanger Centre 1994, p. 14). Even more critical, however, was the shift in data-sharing practices that the authors revealed just three sentences later: “the Consortium will make preliminary sequence data available to the community with the caveat that it is preliminary and may still contain errors. Furthermore, we are willing to help locate genes for persons having a bit of sequence data.” By February 1994, Waterston and Sulston were willing to share their unfinished data with other nematode biologists on request. This type of sharing, while not yet daily release, enabled other worm biologists to keep track of their progress, while also identifying biologically interesting elements in their own and others’ worm sequences.

A move to daily release took place over the following year. In late 1994, Sulston attended a workshop on human chromosome 22 and commented to Waterston afterwards, according to the latter’s personal archive, that “a key step in gaining the trust of the community was to make it an absolute rule that all the sequence produced at the Sanger Centre, whether from worm, yeast or human … be immediately released into the public domain” (Waterston to authors Feb 2017). As the public databases did not yet accept unfinished sequences, the duo resorted to posting this data on their personal laboratory websites via file transfer protocol (FTP) (Abbate 1999; Berners-Lee and Fischetti 1999). They continued to submit finished sequences to GenBank and the EMBL; the European leg of the DNA database tripod, moreover, became a close neighbor, when the EMBL opened its European Bioinformatics Institute (EBI) next door to the Sanger Centre in Hinxton (García-Sancho 2015, p. 130). Waterston recalls that this crucial shift, to daily sharing in late 1994, was likely “the result of improved initial assemblies (due to PHRAP and PHRED) and a positive experience with our sharing of the sequence with the community. It may have also been triggered,” he recalled in 2017, “by our increasing involvement with human sequencing” (Waterston to authors Feb 2017).

While the exact causes of this transition do prove difficult to document, we can pinpoint Sulston and Waterston’s report of their strategy in late 1995. Thanks to the accomplishments of the Sanger and St. Louis centers in early human sequencing, alongside some heavy pressure from Maynard Olson, the NCHGR initiated a grant competition for larger-scale, but still pilot, human sequencing at this time (Marshall 1995; Olson 1995). The major purposes of these pilot grants, according to an “Interim NCHGR Policy” statement, were to “have a well-tested approach, as well as a number of experienced groups,” for full-scale human genome sequencing, and through these groups to complete approximately 3% of the human genome before scaling up the effort to a larger number of laboratories (1995 Interim NCHGR Policy). Technology development was another priority. In their successful joint proposal to the NCHGR, “The Human Genome Sequence-A Pilot Project,” Sulston and Waterston promised that in order “to provide early access to partially finished and partially contiguous data”:

Contigs longer than 1 Kb [one kilobase (Kb), or one thousand base pairs] will be posted immediately … after … checking, but independently of whether the data has been reviewed further for content. This is a policy that was instituted for the *C. elegans* project and has been well received. After manual finishing, new sequences will be analyzed for the last time, annotated with manual review and submitted to public repositories promptly within two weeks. (Waterston to authors Jan 2017)

This pilot sequencing grant ran until 2000, and beginning in 1997, the authors confirmed in abstracts for their annual renewals that this data-sharing approach drew directly on the strategy they adopted in the worm: “the essence of our approach will be to utilize the methods with which we have completed over 20 Mb of the *C. elegans* genome in the past 2 years” (Project Information, 5P50HG001458–02 1997). In a 1996 press release about the pilot projects, the NCHGR noted similarly of Waterston’s St. Louis laboratory that: “The world’s largest contribution to sequencing of a single genome has been made by this group, in collaboration with … the Sanger Centre…. Together they have completed over 37 million bases of the nematode worm…. Rapid public release of data is their hallmark” (1996 NCHGR Pilot Study Press Release).

These practices set powerful precedents, particularly for the other centers piloting human sequencing. By late 1995, Waterston and Sulston had jointly produced more DNA sequences for a single genome than any other collaborative unit in the world. A public-nonprofit partnership had completed the *Haemophilus influenzae* genome by 1995 (Fleischmann et al. 1995). The *E. coli*, fly, and *Arabidopsis thaliana* projects, the latter not yet officially a part of the HGP, were also underway (Blattner et al. 1997; Arabidopsis Genome Initiative 2000; Rhee et al. 2003). But the Sanger and St. Louis centers, alongside Olson’s group, had also contributed substantially to the nearly completed yeast genome, and the *C. elegans* genome, their hallmark project, was nearly an order of magnitude larger than this (Goffeau et al. 1996). Sulston, moreover, was preparing to tackle “the longest contiguous piece of human DNA ever sequenced,” the 2.2-million base pair segment of chromosome 4 associated with Huntington’s disease, and Sulston and Waterston’s next goal was human chromosome 22 alongside regions of the X chromosome (Marshall 1995, p. 784; 1996 NCHGR Pilot Study Press Release). By August 1995, Sulston’s team had completed nearly 1 Mb of human genomic sequence, while Waterston’s team had added another 250 Kb (Waterston to authors Jan 2017).

Daily sharing of unfinished data, however, was sometimes more aspirational than actual. In late 1995, the Sanger Centre issued a press release confirming the daily sharing practice for both the Cambridge and St. Louis laboratories, in relation to a collaborative sequencing project on the breast cancer gene, *BRCA2* (Waterston to authors Feb 2017). “In accord with our standard practice of releasing data to the scientific community as quickly as possible,” the communication read, “the DNA sequence is being made publicly available by ftp at the 2 centres in preliminary form…. Importantly, Drs. Wooster and Stratton,” who were collaborating with the Sanger Centre and St. Louis on the *BRCA2* project (Wooster et al. 1994, 1995; Stratton et al. 1994; Wooster and Stratton 1995; Davies and White 1996; Parthasarathy 2017), “have agreed to immediate release of the data, without delay or prior access” (Waterston to authors Feb 2017; also, Durbin interview 2012). In January 1996, however, at a UK summit on human genome analysis documented by LaDeana Hillier, the Sanger Centre reported that its sequencing data went “to the ftp server weekly (sanger) and nightly (st. louis)” (Hillier 1996 Notes; Waterston to authors Feb 2017). The disparity was small, but nonetheless real. One month before the First International Strategy Meeting on Human Genome Sequencing, where the Bermuda Principles for daily data sharing were initially ratified for the HGP, the largest DNA sequencing center in the world was releasing its unfinished data weekly rather than daily.

Both Sulston and Waterston strove for daily release, but pre-publication sharing was the more important outcome. It created common resources in the nematode and human genomes, and in so doing helped to coordinate these large-scale, distributed, and novel projects while also helping to delineate, for the data’s users, between sequences of unfinished and finished quality. It must be noted that many in the worm community were dedicated to a “democratic” biology, as the FOSS bioinformatics teams had been. They were skeptical of the patent claims proliferating on DNA sequences, such as those for the breast cancer genes *BRCA1* and *BRCA2* (Davies and White 1996; Parthasarathy 2017). For John Sulston, these beliefs were rooted in the socialist politics he had embraced since at least the 1960s, described in his autobiographical account published with Georgina Ferry in 2002 (Sulston and Ferry 2002; also, Sulston and the International Human Genome Sequencing Consortium 2001, pp. 28–31). Jane Rogers, who organized and managed the 17 sequencing groups established at the Sanger Centre by the mid-1990s (3 for yeast, 4 for nematode, and 10 for human), recalled in 2012 that for Sulston, rapid sharing was, “a continuation of … the hippie culture of freedom” (Rogers interview 2012). Those anxious about the commercialization of science, however, had little to worry about with regards to worm research. There was no billion-dollar market for nematode insulin or growth hormone; much to Sulston’s chagrin, the Wellcome Trust had even refused to fund *C. elegans* sequencing in the early 1990s because it lacked obvious medical applications (Morgan and Wallace interview 2012). Pragmatic justifications for data sharing thus prevailed in nematode biology, spilling into human biology through the Cambridge and St. Louis sequencing centers. But in its generalization to all of the HGP’s human-sequencing centers, the daily-sharing standard, as enshrined in the Bermuda Principles, encountered new complications. The origins of these complications reached to the very foundations of the HGP: its dual roles as a publicly funded infrastructure project and as a driver of economic growth, and, perhaps counter-intuitively, the attendant ambiguities about sharing and the commons that had plagued it from the beginning.

## Data Sharing in the Early HGP (1985–1990)

In 1985, discussions of a human genome project were already churning in the United States.^28^ In May of that year, Robert Sinsheimer, a molecular biologist and also the chancellor of the University of California at Santa Cruz, pitched an idea for a project to sequence the human genome (Cook-Deegan 1994, Chap. 5, esp. pp. 79–80). The ensuing discussions, which included John Sulston and Robert Waterston, followed a 1984 conference in Utah on whether new analytical DNA methods “could … permit direct detection of mutations” or “any increase in the mutation rate among survivors of … Hiroshima and Nagasaki” (Cook-Deegan 1989, p. 661). The DOE Office of Health and Environmental Research (OHER) funded the 1984 meeting in Utah, but the findings appeared in a report of the Congressional Office of Technology Assessment (OTA) 2 years later, in 1986 (*Technologies for Detecting Heritable Mutations in Human Beings*). Reading a draft of this document, Charles DeLisi, the new OHER director, was struck with his own idea for a “dedicated human genome project” within the DOE (quote in Cook-Deegan 1989, p. 662; also, DeLisi 1988). The proposal built on a long legacy of DOE patronage in biology, one in which the histories of twentieth-century particle physics and molecular biology were intimately linked (Heilbron and Kevles 1988; Creager 2013).

During the Second World War, and through the Office of Scientific Research and Development (OSRD), civil servants at the Los Alamos National Laboratory (LANL) and elsewhere were tasked with investigating the biological effects of radiation (Cook-Deegan 1994, Chap. 7; also, Kevles 1995; Rasmussen 1997; Creager 2013). Under the persistent Cold War threat of nuclear war, the field of radiation biology continued after 1945, thanks to the Atomic Energy Commission and the eagerness of physicists to work towards peaceful applications for nuclear energy. By the 1970s, a complex sequence of legislative rearrangements had created the DOE, placing a large network of national laboratories under its jurisdiction (Pub. L. No. 79–585; Pub. L. No. 83–703; Pub. L. No. 93–438; Pub. L. No. 93–577; Pub. L. No. 95–91).^29^ By the end of this decade, the NIH had also augmented the DOE’s investments in quantitative biology, when the LANL physicist Walter Goad and the company Bolt, Beranek, and Newman (BBN) engaged in a competition with the *Atlas of Protein Sequence and Structure* creator, Margaret Dayhoff, for NIH funding to host the US database that would become GenBank (Dayhoff et al. 1965; Smith 1990; Strasser 2011, p. 66; Stevens 2013, Chap. 5, 2017). To make sense of the many DNA sequences already emerging, administrators at the NIH had realized that a centralized nucleotide database was paramount; and collection and comparison, the centerpieces of natural history, would dominate as analytical tools, building on the rich traditions in these areas from molecular phylogenetics and protein sequencing (Dietrich 1998; Suárez-Díaz 2007; Suárez-Díaz and Anaya-Muñoz 2008; Stevens 2011). The LANL team won the contract, which began in 1982 (Strasser 2011, pp. 89–90), but which also raised novel questions about the relationships between scientific data sharing and journal publication.

Key differences in the Goad-BBN and Dayhoff proposals involved the collection, ownership, and distribution of data. While sequences in the Dayhoff bank were to be collected voluntarily from biologists and also from data published in journals, the Goad team planned to work directly with journals, whose editors Goad hoped would begin to request, and possibly to require, the deposition of sequences to a database (Strasser 2011, esp. pp. 81–86). While neither team proposed that the NIH retain the right to reproduce the sequences (copyright), and both indicated that the data would remain in the public domain, only Goad’s team promised online distribution and accessibility.^30^ Dayhoff’s team, in contrast, proposed paper distribution, as had been the case with *Atlas of Protein Sequence and Structure* (Dayhoff et al. 1965). For a combination of these reasons, the NIH preferred Walter Goad’s proposal. The upshot was that it fit more cleanly within the moral economy of molecular biology, wherein publication, rather than database deposition, provided the hallmark form of scientific credit (Strasser 2011, p. 83). While protecting the hegemony of the journal (Hilgartner 2017, Chap. 6, esp. pp. 155–165), however, Goad’s team also prioritized widespread and rapid circulation of data, a goal the NIH shared.

This priority carried into the first renewal of the GenBank contract. In 1984, the NIH contracted with the company, IntelliGenetics, to run BIONET.^31^ BIONET built on connections to FOSS through the Stanford Artificial Intelligence Laboratory (SAIL), and grew into one of the most trafficked thoroughfares to GenBank by the late 1980s (Stevens 2015a). The network let investigators upload their own sequences to GenBank, a major reason that the LANL, one of the winners (through Goad) of the first GenBank contract, had come to partner with IntelliGenetics in the first place. The IntelliGenetics-LANL team also won the GenBank contract from 1987 to 1992, an agreement which lasted through the first few years of the HGP. In 1992 the NIH itself, through the National Library of Medicine (NLM), became home to the American database (Strasser 2011, p. 90; Stevens 2013, pp. 162–164, 2015c, p. 476). None of these various institutions, however, ever suggested distributing unpublished data (Strasser 2011, p. 83). Even the Protein Data Bank (PDB) at the Brookhaven National Laboratory, which had been hosting and distributing both unpublished and published crystallographic coordinates since 1973 (Berol 2001, Chap. 6; Berman et al. 2004; Strasser 2011, p. 67), restricted some of its data for up to 4 years *after* publication, enabling the generators to continue to interpret and publish freely from it during that time (Strasser 2011, p. 84).^32^

As sequences grew easier to produce, however, their scientific value changed (Colwell 1989; Ankeny and Leonelli 2015; Stevens 2013, Chap. 5, 2015a). This transformation was due in part to journal editors, who by the late 1980s had begun encouraging the direct deposition of DNA sequences to GenBank (Lewin 1986; Burks 1989; Marshall 1990a, b; Ankeny and Leonelli 2015, pp. 127, 130–132). By 1992, at least 36 journals required deposition of sequences to databanks “as a condition of publication” (quote on Hilgartner 1995b, p. 253; also, McCain 1995); and the CSHL-based journal, *Genome Research*, was even founded with its own database companion (Hilgartner 1995b, p. 258). Raw DNA sequences were simply taking up too many pages, and were largely useless without computer analyses. Yet, with only a few exceptions, these new policies were intended to create supplements, not substitutes, to publications, usually between 6 months to a year after the corresponding journal articles had appeared in the literature (Hilgartner and Brandt-Rauf 1994; McCain 1995, pp. 411–412, 416). The editor of *Nature*, John Maddox, bucked even this trend. He refused to require the deposition of sequences to public databanks, a decision that resulted in delays of up to a year (or even more) before some published sequences appeared in the public domain (Maddox 1989a, b; McCain 1995, p. 415, Appendix B; Stevens 2015a, p. 857). As voluminous as nucleotide sequences were becoming, molecular biology was simply too competitive, and too commercially valuable, for a data deposition requirement, which Maddox understood as beyond the “journal’s jurisdiction” (Hilgartner 2017, p. 162). Despite GenBank’s close connections to electronic data sharing, therefore, and despite the fact that such databases were essential for enabling rapid sharing in general (Stevens 2015a, c, 2017), the Bermuda Principles were not a direct product of database policies and technologies.

The US HGP continued to gestate through 1986 and 1987. In 1986, the DOE announced its Human Genome Initiative (HGI), allocating $5.3 million to the national laboratory network for resource and technology development (*Human Genome Program Report*, ii). Also in 1986, the National Research Council (NRC) of the US National Academy of Sciences investigated the feasibility of mapping and sequencing large genomes, with Sydney Brenner representing the viewpoints of the UK mappers and sequencers and Maynard Olson bringing further first-hand experience to the study.^33^ In 1987, a DOE committee recommended a 15-year project to map and sequence the human genome (*Human Genome Program Report*, ii). Shortly thereafter, the DOE and the NIH began allocating explicit funding for mapping and sequencing, securing their first official HGP budgets from Congress (Cook-Deegan 1994, pp. 99–106, 142–147, 355–356). Finally, on August 7, 1987, the OTA hosted the workshop that dovetailed from the notorious 1986 CSHL symposium of $3 billion-price-tag fame, organized by David Guston (at the time an OTA intern) and chaired by Paul Berg of Stanford University (*Mapping Our Genes*, *Transcript of Workshop*). John Sulston (who was still at the LMB) discussed data sharing and the technologies likely to be required for successful and efficient human genome sequencing, and other attendees included Christian Burks (GenBank), Walter Gilbert (Harvard), Ruth Kirschstein (NIH), and James Watson (CSHL). No one in the yeast community, such as Maynard Olson, attended, and John Sulston was the only physical mapper present.

In 1988, in a crucial pair of reports, both the NRC and the OTA formally endorsed a human genome project. The NRC report enjoyed input from Sydney Brenner and Maynard Olson. Alongside investment in DNA sequencing technologies, the NRC recommended the production of a reference human genome via the map-then-sequence strategy (*Mapping and Sequencing the Human Genome*, Chaps. 4–5, esp. pp. 37–40, 56, 60–65; Olson et al. 1989). While the *E. coli* physical map was the largest completed to date, the bacterial genome was estimated to be three orders of magnitude smaller than that of the human (*Mapping and Sequencing the Human Genome*, p. 51; Hilgartner 2004a, pp. 114–115, 2017, Chap. 2, esp. Table 2.2). Echoing practices in worm and yeast mapping, the NRC recommended that a distributed network of laboratories, coordinated by rapid sharing, carry out the project, noting: “essential to the success of the project will be cooperation between laboratories … and the ready availability of data and materials to all participants” (*Mapping and Sequencing the Human Genome*, pp. 99–100).

A crucial difference, however, separated the yeast and nematode genome projects in the 1980s. The “international yeast community” had been meeting regularly since the early 1960s (von Borstel 1993; Weinberg 1993, p. 72). The community had long encouraged the timely sharing of techniques and yeast strains, alongside the publication of maps and early sequences in the journal *Yeast*.^34^ Yet while the yeast guru Maynard Olson usually shared his physical maps with collaborators before publication, he never released data on a daily basis, due mostly to quality concerns (Olson to Waterston 1996). Delays also took place in the yeast genome-sequencing project, which was established within the European Community (EC) by 1989 with additional laboratories in Canada, Japan, and the United States (Anderson 1992; Kahn 1996; Joly and Mangematin 1998). Whether because of issues with credit distribution or journal priority, some yeast sequences failed to appear in the appointed database of the Martinsried Institute for Protein Sequences as rapidly as certain participants, particularly those who appreciated the norms of the nematode community, would have liked (Anderson 1992; Cook-Deegan 1994, p. 201).^35^ Yet as Olson noted in a news feature in Science in 1992, so long as the data was available at publication, the EC yeast project was still consistent with “standard scientific practice” (Anderson 1992, p. 462).

Large-scale genome sequencing, in short, was uncharted territory. In England, Rodger Staden had helped Frederick Sanger begin computerizing his sequencing methods in the late 1970s (Staden 1979; García-Sancho 2015, esp. pp. 83–93). By 1982, however, Sanger sequencing had only been tested on phage and mitochondrial DNAs (García-Sancho 2015, Chap. 3, esp. pp. 71–76, and 121). Further-automated Sanger sequencing, spearheaded in the laboratory of Leroy Hood at the California Institute of Technology, was just beginning to garner results (García-Sancho 2015, pt 3; Smith et al. 1985, 1986). The term “genomics” was coined only in 1987, when the leading geneticists Victor McKusick and Frank Ruddle named a journal, *Genomics*, after “the newly developing discipline of mapping/sequencing” (McKusick and Ruddle 1987, p. 1); and the longest known human sequence—of the growth hormone gene—stretched only to 67,000 nucleotides (*Understanding Our Genetic Inheritance*, p. 6). In the late 1980s, various credible views existed on how best to handle genomic data. Some experts, such as John Sulston, Robert Waterston, and others in the *C. elegans* community, veered far from the academic norm of sharing data at the time of journal publication. Other individuals, including Maynard Olson, tended to hem much closer to it.

Beyond exhortations for sharing data, the 1988 NRC report failed to articulate a clear data-sharing policy. It stressed the creation of a commons, “to be used by biomedical scientists to accelerate the understanding of human biology and the application of this knowledge to human health,” alongside a central database for sharing maps and sequences (*Mapping and Sequencing the Human Genome*, pp. 82–84, 91). It also noted that the project might generate “materials of potential commercial value,” such as “clones that encode previously undiscovered hormones, growth factors, or mediators of immunity” (p. 99). But on the matters of “copyright protection of the data and … intellectual property,” the NRC was strikingly vague (pp. 99–100). “Sequences and material generated by … publicly funded projects should and even must be made freely available,” the report asserted (p. 8). But no timeline was provided, despite the report’s insistence that “a single, unified policy must prevail if the information is to be accurately acquired, stored, analysed, and distributed” (p. 85).

In 1980, several events coincided to raise the commercial value of molecular biology, and especially medical and human genetics, extensively (Cook-Deegan and Heaney 2010, pp. 391–394; Rasmussen 2014; Sherkow and Greely 2015, esp. pp. 165–166). These events are often credited with launching the biotechnology industry in the US, which has a history closely linked to that of the HGP (Gaudillière 2009; Hughes 2011; Popp Berman 2012; Rasmussen 2014; Yi 2015). One of these events was the US Supreme Court’s decision in *Diamond v. Chakrabarty* (447 U.S. 303 (1980)), which declared that a General Electric patent, claiming a bacterium engineered to digest hydrocarbons, was valid: the first US patent on a man-made living object (Kevles 1994; Sherkow and Greely 2015, p. 165, n. 35). A second was the issuing of the first patent (of an eventual three) related to recombinant DNA (rDNA) methodologies granted both to Herbert Boyer of the University of California, San Francisco (UCSF) and Stanley Cohen of Stanford University (Cohen and Boyer 1980). This occurred just 6 weeks after the initial public stock offering for Genentech, one of the world’s first biotechnology companies, which was based largely on rDNA technologies and co-founded by Boyer and the venture capitalist Robert Swanson (Hughes 2001, 2011). A fourth, final event was the signing of the US Bayh-Dole Act (Pub. L. No. 99–517). By allowing grantees and contractors in nonprofit institutions, universities, and small businesses the first right to patent inventions drawing on publicly funded science, the Bayh-Dole Act sought to increase the public’s return on research investments, in particular through commercialization following downstream development of basic research.

These events opened new scientific and commercial vistas based on the manipulation of DNA. Recombinant DNA, and the inventions it generated, was a major step in the application of molecular biology to higher organisms (Morange 1997, 2000, 2008). Few US patents covered full-length human genes before 1990, but afterwards more than 1,000 patents claimed genes or nucleic acid sequences “focused on the workhorse tools of molecular biology: cloning vectors, bacterio- phage DNA, and purified plasmids” (Sherkow and Greely 2015, p. 166; see also, Cook-Deegan et al. 1996). The first patent on a human gene, *CSH1*, was granted to UCSF researchers in 1982. *CSH1* codes for the fetal growth and development protein, chorionic somatomammotropin, and the patent in question covered the “654 base-pair sequence of the gene, as well as technologies to make recombinant versions of the hormone in bacteria” (Ibid.). The historian Nicolas Rasmussen has chronicled the history of rDNA cloning for many such therapeutically valuable proteins, including for insulin, interferon, tissue plasminogen activator, erythropoietin, and human growth hormone (2014). These developments reflected the sparkling commercial potential of the new molecular biology, but in turn fueled uncertainty and outright alarm regarding the issues of sharing and secrecy. A 1982 summit in California had already addressed the widespread concerns about biologists in start-up advisory roles (Culliton 1982). In 1985, the American Association for the Advancement of Science issued a report on the “causes and effects of secrecy and openness” (Chalk 1985, p. 29); and soon after that, the sociologist Stephen Ceci reported that losing out on patents or funding was the most significant reason that individuals in biotechnology failed to share their data (1988).

These worries spilled over into the 1990s, as biologists whose research applied clearly to humans faced cutthroat workplaces where data, materials, and techniques were often kept from competitors, even after publication.^36^ Researchers raced to find and sequence the genes deemed responsible for diseases such as cystic fibrosis, Huntington’s disease, neurofibromatosis, and muscular dystrophy (Davies and White 1996; Hilgartner 2004a, b, 2017, Chap. 2, esp. pp. 47–52; Lindee 2005; Wailoo and Pemberton 2006; Comfort 2012; Rasmussen 2014). The processes of mapping, localizing, and sequencing a gene, wherein craftwork still dominated over automation, could take up to 10 years to complete (Hilgartner 2004a, pp. 117–118, 2017, Chap. 2). The “zero-sum” enterprise of human gene mapping gave rise to strategies for *not* sharing data (Hilgartner 2004a, p. 119, 2017, pp. 34–36): a pervasive problem that persisted into the 2000s and beyond, and stimulated initiatives, such as Vice President Joseph Biden’s Cancer Moonshot, emphasizing rapid sharing and the potential destructiveness of hoarding data and resources in biomedical science (Campbell 2002; Stokstad 2002; Cancer Moonshot 2017; Cook-Deegan et al. 2017; Lowy and Singer 2017). Enthusiasm over the economic potential of medical genetics in the late 1980s and early 1990s paired with painful uncertainty about how best to reach it, with careers on the line and data-sharing strategies ranging from rapid, pre-publication data release to outright hoarding and secrecy.

The 1988 OTA report, *Mapping Our Genes*, also reflected this apparent tension, between rapid data sharing and commercialization in the biomedical sciences. The authors hoped “a large Federal investment in genome projects” would be “translated efficiently into new products and services … creating new jobs and other economic benefits,” but conceded that the “uncertainty about the magnitude of economic impact means that genome projects cannot be justified purely as an economic investment” (p. 165). The OTA report envisioned a “clear role for congressional oversight … in ensuring that data are shared promptly and fully,” but suggested only that the “disclosure of *data* should not be long delayed by policies designed to encourage patenting,” because “data per se are not eligible for patent protection” (16, p. 168). A timeline and policy for data sharing in genome projects once again remained elusive, as had occurred with the NRC’s report on the same topic, from that same year.

The HGP began officially in 1990. The initial planning document prepared by the NIH and the DOE, *Understanding Our Genetic Inheritance*, implicitly acknowledged the NIH as the lead agency, with an NIH-to-DOE funding ratio of 2:1 (pp. 30–37). The National Science Foundation (NSF), National Institute for Standards and Technology, and several Department of Defense research and development agencies were also expected to play less conspicuous roles (Palca 1989). The main goal of the new project was to develop a human reference genome, to be accomplished by a “multi-phase program” in which the initial phase would encompass refining the genetic map, beginning the construction of a physical map, and investing in more accurate, efficient, and affordable DNA sequencing (*Understanding Our Genetic Inheritance*, pp. 5–6). Partly to encourage data sharing and quality control, a digitally compatible language of “sequence-tagged sites” (STS) would help to decouple physical mapping from material clones (Olson et al. 1989, p. 1434; *Understanding Our Genetic Inheritance*, p. 11; Hilgartner 1998, pp. 208–210, 2004a, pp. 124–125, 2013, esp. pp. 406–407, 409, 2017, pp. 96–107; Bostanci 2004, pp. 159–161).^37^ Finally, the HGP was to absorb five model organism projects, both for comparative genomics and for technological guidance in the map-based sequencing strategy (Cantor 1990; Watson 1990; Lewin 1990; Ankeny 2000, 2007). Completion of the human genome sequence was set for 2005, with a budget of approximately $200 million annually for 15 years from 1990 (*Understanding Our Genetic Inheritance*, ix; Bostanci 2004, p. 162). The “basic data produced” from the project, the 1990 NIH-DOE report described rather vaguely, would “be collected in electronic databases that will make the information readily accessible in convenient form to all who need it” (vii-viii).

With its peer-reviewed grant structure, long-term goals, technological optimism, and predominant reliance on public funding, the HGP bore a marked likeness to the “big” scientific projects of the post-World War II era (de Solla Price 1963; Weinberg 1967; Heilbron and Kevles 1988; Capshew and Rader 1992; Galison and Hevly 1992; Balmer 1996a, b; Kevles 1997; Strasser 2003; Jasanoff 2005; Bartlett 2008; Vermeulen 2009; Aronova et al. 2010; Hilgartner 2013, 2017; Vermeulen and Penders 2013; Aircadi and García-Sancho 2016; Leonelli 2016; Shaw 2016). Many of the molecular biologists accustomed to bench-level science feared that the HGP was an insatiable monster, an inexorable drain on grant funding for smaller laboratories that would drown out research on smaller, more hypothesis-driven projects (Watson 1990, p. 45). While the HGP’s leaders proudly waved the “big science” banner later on (Green et al. 2015), in the early phases of the project they assiduously avoided this image (*Mapping and Sequencing the Human Genome*, pp. 23–24, 87; *Mapping Our Genes*, pp. 6, 10). An initial 3% of the budget was dedicated to addressing the ethical, legal, and social implications (ELSI) (*Understanding Our Genetic Inheritance*, p. 20)—a number raised to 5% in 1992 (Cook-Deegan 1994, p. 255)—given the massive commercial potential, complex symbolism, and opportunities for discrimination associated with determining the full DNA sequence of the human genome (Kevles and Hood 1992; Cook-Deegan 1994, Chap. 16; Reardon 2005, 2017).^38^ The staggering cost of the project was further justified with the promise of generating public resources, but no systematic data-sharing policy yet existed.^39^

## The Bermuda Meetings and Principles

The HGP consolidated many international programs and initiatives, expanding the bureaucratic umbrella of the project quickly to beyond just the United States. Indeed, the 1988 founding reports from the NRC and the OTA had encouraged and anticipated an international effort, marking collaboration as crucial, even as they emphasized the importance of genome research for American economic interests in a growing and global biotechnology sector (*Mapping and Sequencing the Human Genome*, Chap. 7; *Mapping Our Genes*, Chap. 7).^40^ By 1990, the DOE had established three genome analysis centers at the Berkeley, Livermore, and Los Alamos National Laboratories, working mostly on physical mapping and technology development (*Understanding Our Genetic Inheritance*, pp. 27–33, esp. p. 31). The NIH had planned between 10 and 20 Genome Science and Technology (GESTEC) centers of its own, each set to address a discrete task such as the physical mapping of a single chromosome. The concurrent efforts in Britain in human and model organism genomics were also highly advanced, beyond the *C. elegans* tradition that had been spearheaded by Sydney Brenner and perpetuated by John Sulston, Alan Coulson, and others. For example, the Austrian geneticist Hans Lehrach led the Genome Analysis Laboratory at London’s Imperial Cancer Research Fund (ICRF), boasting a “reference library” of data and materials for physical mapping in the fission yeast, *S. pombe* (Kahn 1995, p. 1557; Hilgartner 2004b).^41^ Another effort in the U.K. was the MRC-funded Human Genome Mapping Program (HGMP), which was heavily orchestrated by Brenner and the geneticist Walter Bodmer, then the director of research at the ICRF, and focused on the localization and sequencing of highly expressed genes (Galloway 1990; Rees 1991; MRC Review 1992; Balmer 1996a, b, 1998; Balmer et al. 1998; Hilgartner 2017, pp. 137–139). After becoming the director of an MRC Molecular Genetics Unit in Cambridge in 1986, Brenner had begun pushing for funding for a publicly available library of cosmids, to be used as a national resource for human gene mapping in the UK (Balmer 1996a, p. 535; 1996b, p. 258). The resulting HGMP maintained British competitiveness in genomics without an exorbitant outlay of funds, with the bulk of the cost totaling £11 million annually over three years (Balmer 1996a, p. 535). For Brenner, the HGMP primarily represented a public infrastructure project, but thanks to its emphasis on genes, the initiative also held commercial potential that overcame the skepticism towards basic science that had generally been evinced by Prime Minister Margaret Thatcher’s administration (Balmer 1996b, p. 255). Inaugurated in 1989, the HGMP relocated to the Sanger Centre in Hinxton in 1994 (Balmer 1998, p. 17).

Genomics in France and Japan around 1990 each reflected a similar diversity of actors, funders, and institutions. A private-sector collaboration between the French Muscular Dystrophy Association (AFM), the Centre d’Etude du Polymorphisme Humain (CEPH, now Foundation Jean-Dausset-CEPH), and the laboratory Généthon began in 1990 and was already producing, and would continue to produce, world-class genetic maps (Litt and Luty 1989; Weber and May 1989; Weissenbach et al. 1992; Gyapay et al. 1994; Dib et al. 1996) as well as physical maps (Chumakov et al. 1992, 1995; Bellanne-Chantelot et al. 1992; Cohen et al. 1993; Schuler et al. 1996).^42^ By the early 1990s, the CEPH-Généthon collaboration would generate the first of the partial physical maps of the human genome, and by 1996 it would help to finish a detailed STS-based physical map of the entire genome, in conjunction with a consortium including the laboratory headed by Eric Lander at the Whitehead Institute of MIT (Hudson et al. 1995; Schuler et al. 1996; *Human Genome Program Report*, iii; Rabinow 1999; Kaufmann 2004; Rabeharisoa and Callon 2004). Généthon-generated YACs would became important mapping tools for the human genetics community around 1993 (*Human Genome Program Report*, ii); and after the Bermuda meetings, Généthon-identified genetic markers became a standard mechanism for coordinating human sequencing, allowing HGP centers to identify and “claim” regions of the genome that they planned to sequence (1997 Bermuda Meeting Report; 1997 Meeting Report Summary).

Efforts in Japan, in turn, led the world in other areas of physical mapping, technology development, and sequencing. In 1981, Akiyoshi Wada had secured funding from the Science and Technology Council of Japan through the Science and Technology Agency (STA, or JST [About JST 2009]) for a project called “Extraction, Analysis, and Synthesis of DNA,” which became “Generic Basic Technologies to Support Cancer Research” in 1984 (Cook-Deegan 1994, p. 215). The initiative, closely tied to traditions of robotics and automation in Japan, soon gained industry support, including from Fuji Photo and Hitachi, alongside a headquarters at the elite RIKEN Institute for Physical and Chemical Research in Tsukuba Science City around 1985 (Ibid.).^43^ The Japanese-US competition in genomics was particularly intense, given the trade tensions between the two nations that had lasted throughout the 1980s. Yet as the leader of the Japanese sequencing projects, Akiyoshi Wada had stimulated international efforts to organize a coordinated human genome project, visiting the US in 1986 to promote such collaborations and also to help squeeze genomics funding from his own government (Ibid., esp. pp. 216–218). With support from various other agencies, including the Ministry of Education, Science, and Culture (or Monbusho), in 1991 the STA expanded its DNA sequencing program to include projects in universities and also to mapping in human chromosomes 21 and 22 (Swinbanks 1991; Matsubara and Kakunaga 1992; Cook-Deegan 1994, p. 217). Throughout the 1990s, as a part of the HGP, Japanese investigators also contributed much of the DNA sequence for these areas of the genome (Hattori et al. 2000; Slavov et al. 2000).

The overall coordination of these projects, however, was a hodge-podge (for a summary, see Cook-Deegan 1994, Chap. 18, esp. pp. 288–291). The International Human Genome Mapping Workshops (HGMWs) had commenced in 1973. By the late 1980s, these had spawned the annual Single Chromosome Workshops (SCWs) (MRC Review 1992, pp. 21, 22; Hilgartner 2017, Chap. 3); and, before 1987, data accepted at these meetings was submitted to the Human Genome Mapping Library at Yale University (*Mapping Our Genes*, pp. 157, 189). The Genatlas, which collated genetic and clinical information, grew out of the 1987 HGMW in Paris; and in 1990, the Howard Hughes Medical Institute (HHMI), later joined by the DOE, the NIH, and several other benefactors, established the Genome Database (GDB) at Johns Hopkins University, a repository for maps and related data including STS mapping markers (Brandt 1993; Fashman et al. 1997; Letovsky et al. 1998). The LLNL of the DOE developed Hypertext Markup Language (HTML) connections on the World Wide Web, linking the GDB, GenBank, and Victor McKusick’s *Online Mendelian Inheritance in Man (OMIM)* (Stevens 2015c, p. 481).^44^ McKusick himself founded the Human Genome Organization (HUGO) with backing from the HHMI in 1988 (Cook-Deegan 1994, pp. 123–124, 208–209), an entity that was supposed to coordinate these disparate activities “to encourage collaboration and the sharing of information and resources” (Collins and Galas 1993, p. 44). Finally, several meetings specifically intended to harmonize international genomics took place in the late 1980s, including an opulent conference in Valencia, Spain in October 1988 and a meeting in Moscow, co-sponsored by UNESCO and the USSR Academy of Sciences, in June 1989 (Cook-Deegan 1994, p. 219).

Despite this seeming coordination, however, reality was messy. Various news outlets reported dissatisfaction with HUGO, exacerbated by access problems to the GDB (McGourty 1989; Swinbanks 1990; Anderson and Aldhous 1992). The UK MRC Human Genome Mapping Program, for instance, ran the only GDP node outside of Baltimore (MRC Review 1992, p. 24). While by the early 1990s, some biologists at the Whitehead Institute were writing Perl scripts for sharing physical maps (Crossman and Rai 2005; Stevens 2015c, p. 482), the GDB itself worked best for genetic maps, and the scale-up to physical maps posed substantial challenges (MRC Review 1992, p. 25). Rivalries also persisted. Frenzied competition infiltrated the chromosome meetings, and workshop organizers worried that participants were growing less and less forthcoming with their data (NCHGR Genome Report Card 1992; Hilgartner 1998, pp. 206–207). As the German mapper André Rosenthal recalls, thanks to “competition in positional cloning … resources were hot objects. People fought for them. There were political maneuvers to own these resources and not make them available whatsoever” (Rosenthal interview 2011). When sharing did occur, the process was jumbled. Echoing the methods of the nematode biologists, the HGMP’s scientists and administrators had maintained communication through the quarterly newsletter, *G-Nome News* (which had been called *G-String* for its first three issues) (Balmer 1996a, p. 536, n. 29). Yet more broadly, the coordination amongst human mappers in the early 1990s resembled what Susan Wallace, who was a lead HUGO administrator, remembers: “Everybody got together … in front of their computers. Pieces of paper on the walls. Sat around for a few days, shared what they’d found, wrote up bits and pieces” (Morgan and Wallace interview 2012). These mappers often worked on overlapping sections of the genome to boot, and with divergent methods and technologies.

This disarray brought the Wellcome Trust into closer contact with the HGP. In the early 1990s, the American entrepreneur Frederick Bourke had approached John Sulston and Robert Waterston with a private offer to sequence the human genome, following on their successes with worm sequencing (Cook-Deegan 1994, pp. 333–336; Sulston and Ferry 2002, pp. 81–107; Waterston and Sulston interview 2011; Morgan and Wallace interview 2012). The Wellcome Trust had just rejected Sulston’s request to fund the nematode genome on the grounds of uncertain applications to human medicine; but at James Watson’s urging—and thanks largely to his and Sulston’s desire to keep the human genome in the public domain, rather than under the jurisdiction of Bourke’s proposed private firm—the Trust agreed to a joint venture with the MRC. The Trust had the money, as they had “reorganized their stocks in the late ‘80s” and “needed some decent flagship projects” (Waterston and Sulston interview 2011). The whole of Sulston’s work was transferred to the Sanger Centre from 1993 as a result, with the MRC funding the nematode and the Wellcome Trust sponsoring his human and yeast genomics activities (Cook-Deegan 1994, pp. 48–55, 333–335; Rogers interview 2012).

Sulston and Michael Morgan soon began worrying about the overall organization of the HGP, of which the former’s work now constituted an official part. As human sequencing scaled up, Morgan recalled in 2012, individuals at the Wellcome Trust began having “conversations … about the sensibility of establishing a small international meeting of the main players” (Morgan and Wallace interview 2012). Francis Collins from the NIH participated in these deliberations (Collins interview 2012); and Jane Rogers, who organized the sequencing groups at the Sanger Centre and was thus instrumental in discussions about ramping up the human efforts, recalls that Robert Waterston (who had been visiting the UK), David Bentley, and Richard Durbin were also involved, the latter two as the heads of human genetics and informatics at the Sanger Centre, respectively (though Durbin was also involved in the worm efforts) (Rogers interview 2012). The Wellcome Trust had a tradition of small workshops, called the “Dormy House workshops. They would last two or three days, there would be about 50 participants…. And we usually did it in a very nice hotel in the Cotswolds, it’s called Dormy House” (Morgan and Wallace interview 2012). The inaugural International Strategy Meeting for Human Genome Sequencing, held at the Hamilton Princess Hotel in Bermuda from February 25–28, 1996, grew directly out of this Dormy House tradition.

On those four days, the weather in Bermuda happened to be horrible (McElheny 2010, p. 117). The occasion was not the tropical holiday that some had anticipated, though the gloom did not preclude ginger beer from flowing (Cox interview 2011). The meeting’s main aims were to discuss “mechanisms to co-ordinate, compare, and evaluate … strategies for human genome mapping and sequencing”; to assess “new technologies in sequencing and informatics”; and to weigh “scenarios for data release” (1996 Bermuda Programme and Participants List). Debates and discussions about data sharing, as well as accountings of laboratory resources, funding, and mapping and sequencing progress, permeated the conversations, as demonstrated by the actual transcript of the meeting and other contemporaneous and subsequent documentation, including both Francis Collins’s and John Sulston’s handwritten notes (1996 Bermuda Meeting Transcript; 1996 Wellcome Trust Bermuda Meeting Report; 1996 Wellcome Trust Programme; Collins 1996 Notes; Sulston 1996 Notes). Coordination, managing competition, and preventing duplication were the biggest concerns, especially since 99% of the human genome still needed to be sequenced (Hilgartner 2017, p. 172).

**Fig. 2:**
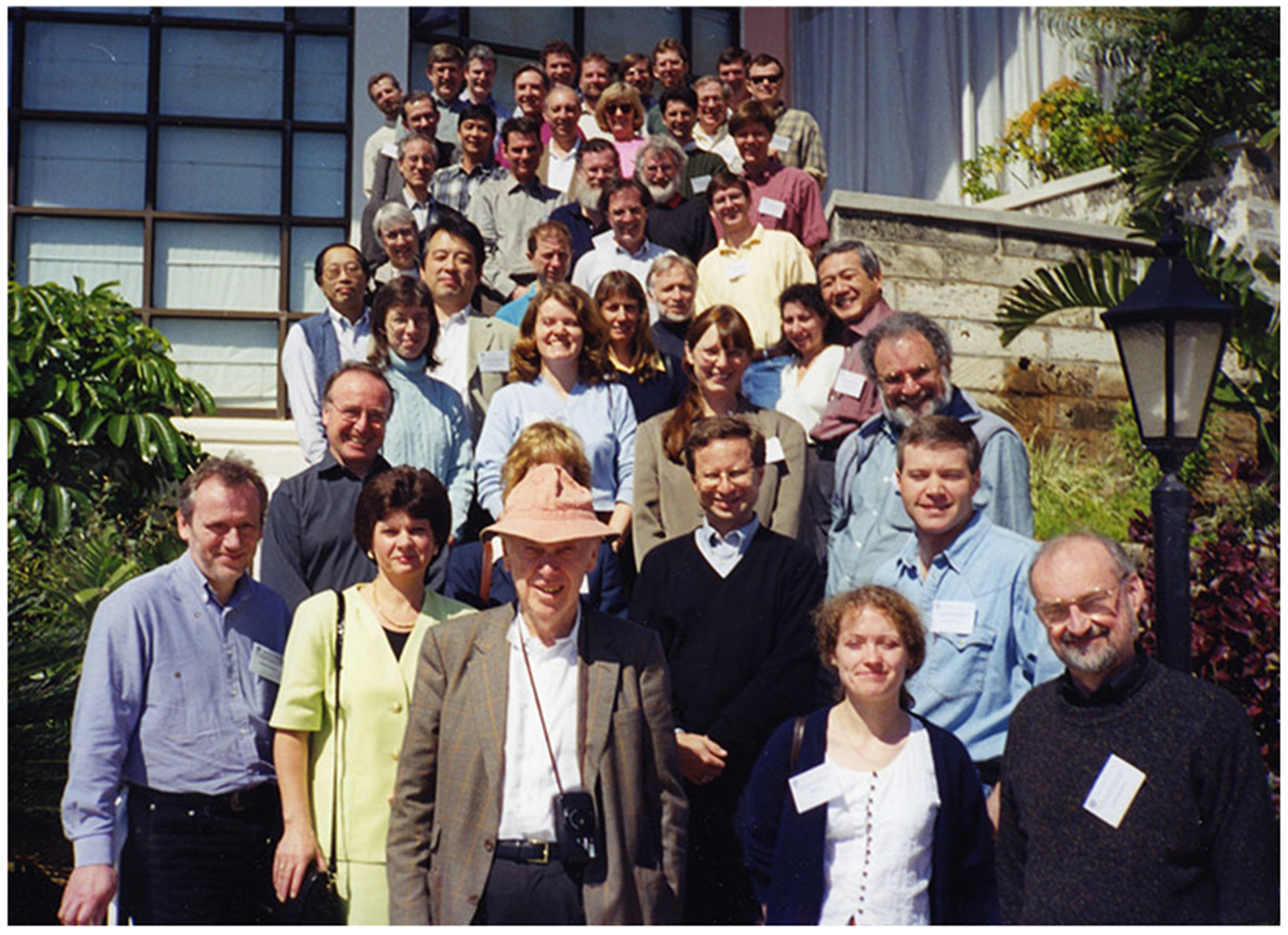
Participants at the first Bermuda meeting, held at the Hamilton Princess Hotel in Bermuda, February 1996. Photograph courtesy of Richard Myers, HudsonAlpha Institute for Biotechnology, reproduced with his permission. Previously published in: Cook-Deegan et al. 2017, Figure 1. Also available from: http://hdl.handle.net/10161/7713

The approximately 50 invitees included genetic and physical mappers, sequencers, bioinformatics and computer experts, and administrators from public, nonprofit, corporate, or other organizations already affiliated with the HGP or intent upon becoming so (Fig. 2; also, 1996 Bermuda Programme and Participants List; 1996 Wellcome Trust Bermuda Provisional Participants List).^45^ The meeting’s opening session incorporated an “enumeration of existing plans,” especially from the “Sanger Centre, St Louis, MIT, LBL [Lawrence Berkeley], Japan, Germany, France, [and] TIGR [The Institute for Genomic Research; see below]” laboratories (1996 Wellcome Trust Programme). The people delivering these updates were mostly white and male, and all hailed from rich nations, yet the size of the assembly was still impressive, given that a large number of genomics experts believed that the mapping-to-sequencing scale-up in the human genome would require a reduction in numbers both to streamline the workflow and to concentrate expertise existing “at the center director level” (Hilgartner 2017, p. 189). Jane Rogers recalls how people in the field at this time were indeed mostly male, though she does remember that genomics administrators at the Wellcome Trust, the NIH, and the DOE, who made most of the invitation decisions, did work hard to invite those whose laboratories had demonstrated the ability and willingness to productively contribute to the mapping and sequencing projects of the HGP (Rogers interview 2012). The Wellcome Trust was the primary sponsor of the meeting, and the selection of Bermuda as the location was supposed to be more “neutral” than convening in either the US or the UK (Branscomb interview 2011; Waterston and Sulston interview 2011; Morgan and Wallace interview 2012; Hilgartner 2017, p. 172). Still, the funding lent an air of Anglo-American dominance, and the locale raised connotations of colonialism. Nevertheless, 6 years after the HGP’s founding report in 1990 (*Understanding Our Genetic Inheritance*), and a decade after the first whispers of generating a human reference genome within the US DOE, this was the project’s largest organizational meeting to date.

The project was transitioning rapidly from mapping to sequencing. Michael Morgan had called a meeting to discuss this scale-up in 1994, partly as a response to the commercial interests in gene patenting that were swelling at the time (Morgan and Wallace interview 2012; Hilgartner 2017, pp. 186–189). The technologies for mapping and sequencing were also improving, building on the early forays into model organism sequencing and the human genetic and physical maps. By 1995, a combination of these factors had prompted Olson, Waterston, and Sulston to call for pilot human sequencing (Olson 1995; Marshall 1995). The grant winners from the resulting NCHGR sequencing initiative were announced in January 1996, right before the first Bermuda meeting, and the funding was slated to begin in April of that year (Macilwain 1996; Marshall and Pennisi 1996; NHGRI Policy Regarding Intellectual Property of Human Genomic Sequence 1996). The grants applied for an initial 2 years, with a third year contingent upon performance (Waterston and Sulston interview 2011). Alongside Robert Waterston at the Washington University Genome Sequencing Center, the Principal Investigator (PI) recipients of the NCHGR grants included Mark Adams of the Institute for Genomic Research (TIGR) in Maryland, Richard Gibbs of the Baylor College of Medicine, Richard Myers of Stanford University, Maynard Olson (by then) of the University of Washington, and Eric Lander of the Whitehead Institute. Lander’s new project in particular would focus on human chromosomes 9 and 17, and “an exportable robotic system that will … have the capacity to sequence human DNA rapidly, accurately, and cost-effectively” (1996 NCHGR Pilot Study Press Release). Lander’s team had already been producing mouse genetic linkage maps of increasing precision (Copeland et al. 1993; Dietrich et al. 1994, 1996), fulfilling one of the HGP’s original goals from 1990 (*Understanding Our Genetic Inheritance*; Hilgartner 2017, Box 4.1). Supported by the NCHGR, these projects had complemented Lander’s leadership in human physical mapping (Hudson et al. 1995; Schuler et al. 1996), and his wide experience now informed his early human sequencing efforts. Sulston and Waterston believed, at the time, that the entire human genome could be sequenced earlier than initially anticipated—that is, before 2005—with only ongoing incremental improvements to prevailing procedures (Marshall 1995).

Predictably, the US cohort in Bermuda in 1996 was the largest, followed by the British (Fig. 2; also, 1996 Bermuda Programme and Participants List). The Americans in attendance included James Watson, who had been the inaugural director of the NIH HGP (1996 Wellcome Trust Bermuda Meeting Report); Francis Collins, now the director of the US NCHGR; Aristides Patrinos, the director of the Office of Biological and Environmental Research (OBER) at the DOE; Mark Guyer, the assistant director of the NCHGR and the program officer responsible for grants administration; Jane Peterson, an NIH program officer overseeing sequencing grants; and J. Craig Venter and Mark Adams, both of TIGR in Maryland. Michael Morgan organized and hosted the meeting on the behalf of the Wellcome Trust, and three colleagues from the Trust accompanied him. Sohaila Rastan joined from the MRC, although this British medical research agency—one of several research councils responsible for academic research and development in the UK (e.g., Balmer 1996b, p. 255, n. 4)—would “not be spending significant amounts of money on production sequencing” in the human genome (Guyer to Collins 1996; also, 1996 Wellcome Trust Bermuda Meeting Report). Four scientists, including John Sulston and Jane Rogers, both of whom attended all three Bermuda meetings, attended from the Sanger Centre, meaning that the NIH, the DOE, and the Wellcome Trust all were able to send both extramural grantees and intramural employees as their representatives to the meeting (1996 Bermuda Programme and Participants List; Rogers interview 2012).

Various German, French, and Japanese institutions were also represented. The German Federal Ministry of Education and Research (Bundesministerium für Bildung und Forschung, BMBF) boasted considerable attendance, including Frank Laplace as the administrator for the German human genome program (DHGP) (1996 Bermuda Programme and Participants List). DHGP biologists in attendance included Hans Lehrach, formerly of the ICRF in London, and André Rosenthal, from the Max Planck Institut für Molekulare Genetik (MPI) in Berlin and the Institute of Molecular Biotechnology (IMB) in Jena, respectively (Unterhuber 1995; Rosenthal interview 2011). Jean Weissenbach was a French genetic mapper at Généthon and also the head of the new French national sequencing center, Genoscope, funded by the basic research agency the Centre National de la Recherche Scientifique (CNRS). Masahira Hattori of the University of Tokyo and Naotake Ogasawara of the Nara Institute of Science and Technology were also at this 1996 meeting, funded by the STA (Hattori to KMJ 2012). Yoshiyuki Sakaki of the University of Tokyo directed the Japanese human sequencing efforts focused on chromosomes 4, 14, 16, 21, and 22, with other aspects of the Japanese HGP under the direction of Ken-ichi Matsubara of Osaka University (1996 Wellcome Trust Bermuda Meeting Report). Neither Sakaki nor Matsubara attended the 1996 Bermuda meeting, though they would have been made aware of the proceedings, and remained closely involved with the HGP’s data-sharing politics in the years to come. While the STA’s funding for the HGP focused on human sequencing, additional support for the Japanese HGP came from the Monbusho (the Ministry of Education, Science, and Culture) and the Ministry of Health and Welfare (Ibid.).

Germany, France, and Japan each had established human sequencing programs soon before the first Bermuda meeting. Germany had arrived late to postwar molecular genetics, a relic of its Nazi past (Jasanoff 1985; Feder 1995; Kahn 1995; Deichmann 1996). The 8-year DHGP, announced in 1995, focused on functional analyses of medically relevant genes (Kahn 1995; Unterhuber 1995; BMBF to KMJ 2012 and 2013). André Rosenthal led the German DNA Sequencing Consortium, alongside Hans Lehrach’s group based at the MPI in Berlin and Helmut Blöcker’s efforts at the German Research Centre for Biotechnology (GBF) in Braunschweig (Unterhuber 1995; Collins to sequencers and data 2000; BMBF to KMJ 2012 and 2013; Lehrach interview 2012). A French national sequencing center had been proposed in 1990, but political changes had caused delays until 1996 (Balter 1998, p. 30). Jean Weissenbach recalls that the newly announced government patronage for HGP sequencing felt quite significant in that year, given the predominantly private and nonprofit backing of French mapping to that point (Weissenbach interview 2012). The Japanese STA had begun funding human sequencing in 1995, under its Genome Frontier Programme (Collins 1996 Notes; Hirakawa et al. 1997; Saegusa 1998b; Hattori to KMJ 2012). Masahira Hattori and Yoshiyuki Sakaki led the principal sequencing group in Tokyo, with additional teams located at Keio University, Tokai University, and the Japanese Foundation for Cancer Research.^46^

The bulk of the Bermuda discussion regarded pragmatics, particularly for quality control, data sharing, and coordination (1996 Bermuda Programme and Participants List; 1996 Wellcome Trust Bermuda Meeting Report; Sulston and Ferry 2002, p. 144; Stevens 2015c, p. 491). The agenda for the meeting included generating an inventory of physical maps, obtaining updates on ongoing disease gene projects, and discussing quantitative tools for data collection and assembly (1996 Bermuda Programme and Participants List; 1996 Wellcome Trust Programme). After James Watson opened the meeting, representatives from each of the laboratories filled out “Resources Available” handouts, indicating “resources available (software, maps, clones etc.)” from each center, any means of requesting and sharing these resources, and “any conditions attached” to the data and materials (Resources Available Handouts 1996). One integral product of the meeting was a new website, the Human Sequencing and Mapping Index, which was supposed to house the Resources Available handouts and provide a forum for the HGP’s centers to notify HUGO, and the project’s other laboratories, of plans to map or sequence certain parts of the genome (Kahn 1996; Skene to Sulston 1996; Bentley et al. 1998; Bostanci 2004, p. 169; Stevens 2015c, p. 491). According to the 1996 Bermuda meeting report, the logic was to “enable centres to declare their [sequencing] intentions in a general framework whilst also allowing more detailed interrogation at the local level” (1996 NIH Bermuda Meeting Report; 1996 Wellcome Trust Bermuda Meeting Report). The website, in other words, would allow the HGP’s centers to notify the other participants of broad plans for sequencing, while also directing users to individual laboratory websites for up-to-date details on progress, difficulties, and changes in plans. Another theme from the 1996 meeting was the dense dialogue on how the five model organism projects, including those for the mouse and the nematode, related to the HGP as a whole.

In Session II, the speakers were asked to “present empirical data from model organisms and explain how these are being used to implement strategies for large-scale human sequencing” (1996 Bermuda Programme and Participants List). In response, a member of the worm project noted how in the nematode, “34 Mb had been sequenced by the two centres (WashU and the Sanger Centre), 6000 genes had been identified,” and “key features of the strategy were the use of multilevel maps (cosmids and YACs) to resolve difficulties and provide a range of resources for use by the scientific community” (1996 Wellcome Trust Bermuda Meeting Report). Along these lines, another member described the worm community’s data-sharing norms:

[W]e rather piously … go on about our communal effort, but actually, we all subscribe to this…. The entire worm community was involved in putting together the genome map, and I do mean the entire, that’s not just … the two central map assembly and subsequently sequencing labs, but also from the beginning of the map, and now the central labs strictly refrain from participating to their advantage in the use of the data. That’s not to say they can’t use it, but they strictly refrain from using it before anybody else does…. That’s why I draw attention to the way that the data is released. And this has allowed, and encouraged, the effect by which the community of molecular geneticists in the worm … have made the links between genes, [on] the functional side, [and] the genetic map and the physical, and subsequently sequenced map…. It’s a communal property. (1996 Bermuda meeting transcript, Session II, “Sequence-Ready Maps”)

Pre-publication sharing had clearly traveled from the larger nematode laboratories to the smaller ones. Analyses took a back seat to sharing, and all the community’s centers enjoyed equal access to the sequences. While there were certainly political, and strikingly Mertonian (Merton 1973), undertones in the language employed by the speaker above (“communal effort,” “communal property”), moreover, the underlying rationale was pragmatic: the efficient and accurate completion of the worm genome. Sulston and Waterston had pioneered this kind of data sharing for their worm and early human sequencing, and at the Bermuda meeting in 1996, they proposed the same strategy for the HGP human genome writ large.

In an “Interlude” between Sessions II and III, documented in Francis Collins’s handwritten notes, the pair proposed the “Timing of Data Release—Immediate (1 kb contigs)” (Collins 1996 Notes). The 1 Kb criterion, representing the shortest contigs thought likely to be accurate, came directly from the worm, as well as from the human sequencing Waterston and Sulston had piloted together following their successful nematode work (Waterston and Sulston interview 2011). Maynard Olson, in comparison, had never even attempted daily sharing in yeast (Olson to Waterston 1996); and with their mouse and “unpublished preliminary versions of STS-based maps” (Hilgartner 2017, p. 106), Lander and his team at the MIT Whitehead Institute had been sharing data through various electronic media at roughly a monthly frequency, but had not yet attempted daily sharing (Lander interview 2012).^47^ Waterston, Sulston, Michael Morgan, and Susan Wallace all remember that the “interlude” in Bermuda in 1996 was meant to include Craig Venter, who had to leave the meeting early (Waterston and Sulston interview 2011; Lander interview 2012; Morgan and Wallace interview 2012). According to both Collins’s and Sulston’s notes, Venter was definitely present at the start of the conference (Collins 1996 Notes; Sulston 1996 Notes). Yet Venter himself does not remember how long he stayed (Venter interview 2012); and James Shreeve, the science writer who chronicled Venter’s role in the HGP, confirms only that Venter was present in Bermuda at some point (Shreeve 2004, p. 46). The *Drosophila* expert Michael Ashburner insists Venter stayed for the whole meeting (Ashburner interview 2011). Whether or not Venter was present, however, the daily data-sharing proposal broached by Sulston and Waterston in the 1996 interlude ignited major fireworks.

Concerns over data quality were paramount. “We have to agree on public release being very important,” one delegate had conceded in Session I, but “we can debate whether or not that should be done hourly, weekly, monthly … every three weeks or so it has to be done” (1996 Bermuda Meeting Transcript). In Session III, one attendee asked, “Why can’t you just finish your BAC … so what if it takes another week? Is that that bad? I don’t want to see sloppy stuff out there. There’s enough of that out there now.” A second cautioned: “All I want to do is make sure that we don’t get so enthusiastic about instantaneous [release], that we run into some other realistic problems about whether people can deliver on that.” Sulston and Waterston’s clout on the issue was clear, but deep reservations about daily data sharing remained. “You’ve got me basically won over,” a third person said. “The two of you are powerful enough that I’m basically won over though I’m a little uncomfortable…. [I]f I’ve got 10 megabases of unfinished but … publicly available sequence … there’s the possibility of … mismatch.” The mouse and human STS projects had just moved from 90- to 30-day release—clearing the NIH-DOE 6-month requirement easily, but also enabling “the chance to make sure” that the data to be released “was somewhat cleaned up for errors and nonsenses”—and the NSF *Arabidopsis* project had recently adopted a 3-month sharing policy. Why not follow one of these alternate strategies, cutting the non-worm biologists some slack? Why daily release? There were other ways. As a fourth individual in Session III noted directly to Sulston and Waterston:

Many people are just, frankly, uncomfortable around the table, not putting out as much data as you two guys do, with the notion of baring their soul on a nightly basis, because then it becomes infinitely obvious that things have screwed up in the past 2 weeks. And I’m quite serious. It’s just too early to press the notion of nightly … what we ought to do is set a precedent of regular [release]. (1996 Bermuda meeting transcript, Session III, “Large-Scale Sequencing”)

Sulston and Waterston, nonetheless, continued in their strident defense of the daily data-release proposal. In Session III, there had been an explicit discussion of how in the *BRCA2* project involving the Sanger Centre, there was definitely a trade-off in balancing the immediate sharing of unfinished data with the later release of more curated data. But, “experience … had shown that such information [released daily] could be effectively utilised by both academic and commercial groups” (1996 Wellcome Trust Bermuda Meeting Report). The Sanger Centre had released 2.7 Mb of finished human sequence into the public domain by 1996, with the goal of scaling up from 20 Mb per year to 100 Mb annually in the next 3 years; and in Session IV, on “Informatics,” a member of the Washington University Genome Sequencing Center noted that its goal was “to provide immediate data release with local annotation of sequence,” the discrete “units of release” including BACs (for human sequence) and “cosmids which could be updated into larger contigs” (Ibid.). Along with James Watson, moreover, Sulston and Waterston chaired the 1996 Bermuda meeting’s fifth and final session, focused on “[c]o-ordination,” “[f]unding agency viewpoints,” and “[c]riteria for comparing cost/accuracy/value of sequence data” (1996 Bermuda Programme and Participant’s List; also, 1996 Bermuda Meeting Transcript, Session V, “Panel/Open discussion; 1996 Wellcome Trust Bermuda Meeting Report). The issues of data release and developing a consistent data-sharing policy for the HGP emerged as key discussion points yet again, with Sulston taking notes on the white board while Waterston directed the conversation and edited the wording for a draft data-release strategy as the final session progressed (Waterston and Sulston interview 2011). The *C. elegans* pair wanted to listen, they recalled in 2011, but were not determined to push a specific agenda. Nevertheless, by the end of this session, the statement that appeared in John Sulston’s loopy handwriting directly echoed the data-sharing strategies from the worm, which had infused this model organism community since at least the 1980s.

**Fig. 3:**
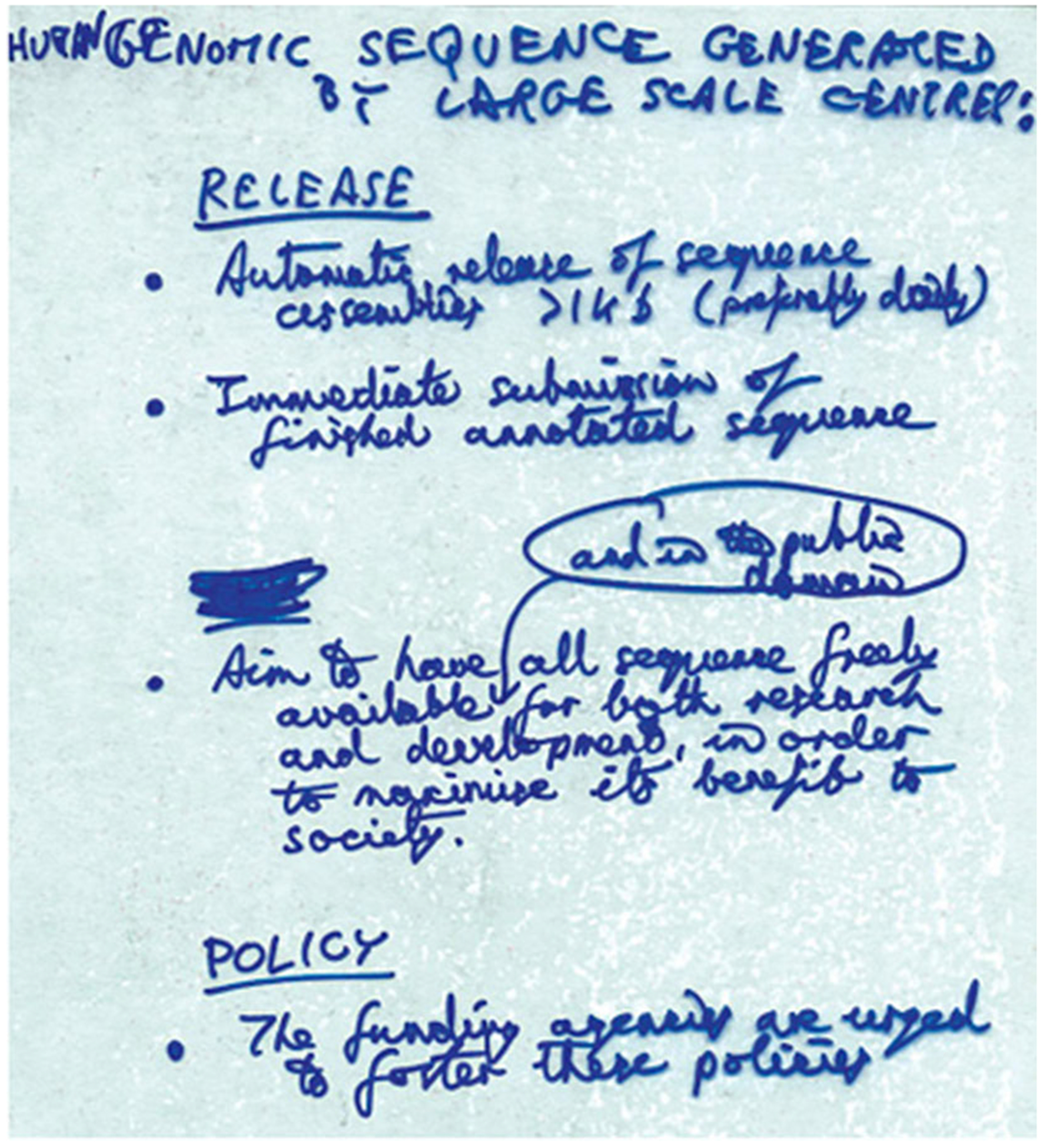
The first draft of the Bermuda Principles, as John Sulston wrote them on a white board in Bermuda in 1996. Photograph courtesy of Richard Myers, HudsonAlpha Institute for Biotechnology, reproduced with his permission. Previously published in: Sulston and Ferry (2002, Figure between pp. 150 and 151 and Cook-Deegan et al. (2017, Figure 2). Also available from: http://hdl.handle.net/10161/7721

The statement suggested that all “human genomic sequence generated by large-scale centres” undergo the “automatic release of … assemblies > 1 kb (preferably daily)” onto individual laboratory websites, with the “immediate submission of finished annotated sequence” to the three public nucleotide databases (Fig. 3; also, Marshall 1996). As Mark Guyer confirmed and elaborated after the meeting:

[D]ata should be released regularly and very quickly from large-scale sequencing projects, perhaps as frequently as daily but maybe weekly would do; this refers to preliminary data (i.e. contigs > 1 kb, not finished to database submission quality) which would be put up locally automatically; it was also agreed that finished, annotated sequence would be immediately submitted to databases. (Guyer to Collins 1996)

This division between unfinished and finished sequences, to be released in different venues and also at different times, harkened back to the earliest days of the *C. elegans* genome project, with the logic once again including, “the need to have buffers between different aspects of the operation” given that the “different cosmids have different problems,” and “we don’t have all parts of the pipeline in perfect balance all the time” (Bentley to Sulston 1996). As Sulston and David Bentley of the Sanger Centre also noted later, while preparing for the 1996 CSHL genome meeting, “the instant release [as suggested in the Bermuda Principles] is a prerelease, for people to use if they want to and to ignore if they don’t. It in no way affects the quality of our finished data,” and “by using the prerelease, we minimize duplication and confusion, and avoid any suspicion of large centres exploiting the sequence unfairly” (Ibid.). The rationales on the white board for aiming “to have all [the] sequence freely available and in the public domain” included the advancement of “both research and development, in order to maximize its [the sequence’s] benefit to society”; and, crucially, “the funding agencies” supporting the HGP’s human sequencing were “urged to foster these policies” (Fig. 3).

Despite this aspirational, rather than literal, interpretation of the daily data-release policy, Maynard Olson actively boycotted the 1996 Bermuda meeting. Perhaps, Olson had failed to realize that Sulston and Waterston intended daily data release as “a prerelease, not the real thing”; or maybe this truly was not clear in the lead-up to the meeting, even though Sulston scribbled to Waterston after the conference that, “heaven knows I thought I said it enough times” (Sulston to Waterston c. 1996). Nevertheless, Olson had had some an inkling of what was to come in Bermuda before the meeting that February, and he explained to Waterston afterward that, “I do not believe that any scientists should ever be forced into what amounts to real-time release of raw data” (Olson to Waterston 1996). He was furious, hated the Bermuda Principles, and also noted later that, “I do not like manifestos and ‘unanimous’ pronouncements on highly technical issues. Most people are simply being rail-roaded under these circumstances, in response to [an] unwise conversion of a technical issue into a moral one” (Ibid.). Olson had no intention of patenting sequences, and favored open access to data (Olson 2002, p. 940). But, as he recalled in 2012, “one needed time to do some basic computational assessments of what kind of data one was releasing” (Olson interview 2012). For sure, he continued in more detail, those overseeing the research needed “more than 24 h to figure out whether or not the machine was running properly or the technician had any idea what he or she was doing” (Ibid.).

Similarly, following the 1996 Bermuda meeting, the debate over HGP data release made its way onto the pages of *Science*. J. Craig Venter and Mark Adams of TIGR, and David Bentley of the Sanger Centre, published dueling opinion pieces on the issue, with Venter and Adams arguing against the Bermuda Principles and Bentley supporting daily release (Adams and Venter 1996; Bentley 1996). Venter likened the Principles to data dumping, a dangerous bifurcation of data production and analysis that was tantamount to bad science (Venter interview 2012). While perhaps possible for larger centers, such as the Sanger Centre, the MIT Whitehead Institute, and the Washington University Genome Sequencing Center, Venter and Adams claimed that daily release was highly inappropriate for many, if not most, of the HGP’s other laboratories, and was therefore an unwise policy for the project overall. “We believe,” Venter and Adams wrote,

there are substantial reasons why scientists should be cautious about using or releasing data and results that have been neither peer-reviewed nor extensively self-reviewed. Although we do not object to the policy of nightly data release adopted by some genome centers, we do object to having these terms applied across the board to all labs involved in genome research. (Adams and Venter 1996, p. 534)

In the aftermath of the 1996 Bermuda meeting, in sum, the human genomics community was split (Kahn 1996). Much to Sulston’s chagrin (Sulston to Waterston c. 1996), in a *Nature* news article, David Dickson publicized even further the idea that according to “some critics, the type of immediate release favoured by Sulston could have unforeseen, and potentially damaging, implications” (Dickson 1996). The schism—which seems to have had more to do with *ideals* about how data *might* be released, than with how data was *actually* going to be released—was similar to that over pre-publication sharing which had divided the nematode and yeast biologists a decade earlier. As with the yeast-nematode discrepancies of the 1980s, moreover, there were real justifications on both sides.

Daily data release would, arguably, help both human mapping and sequencing proceed efficiently and without duplication of work (1996 Bermuda Meeting Transcript, esp. Session V, “Panel/Open discussion”). Sequence volume, including the actual-to-promised output ratios for sequences from each center, would be monitored over the Human Sequencing and Mapping Index (1996 Wellcome Trust Bermuda Meeting Report; Bostanci 2004, p. 174); and individual laboratory websites, where data was supposed to be updated daily, were to report “data quality issues” alongside their “sequence data, particularly in the case of preliminary sequence” (Guyer to Collins 1996). Records are mixed regarding when a formal quality standard for the HGP’s finished sequence was set, with Mark Guyer of the NCHGR noting that, while “the group … [in 1996] seemed to be moving toward agreement that the goal is 99.99% accuracy” (Guyer to Collins 1996), the NCHGR’s pilot projects were determining “whether it will be necessary to strive for 99.99% accuracy” and “whether it can be done cost effectively” (1996 NCHGR Pilot Study Press Release). The 1996 Bermuda meeting report confirmed the quality standard at 99.99%, mentioning a quality-control workshop planned at the NCHGR for that April (1996 Wellcome Trust Bermuda Meeting Report). The report also suggested that “the quality of the data could be determined by the ease of assembly and the use of software programmes … which compared the consensus sequence with the raw data,” being released, ideally on a daily basis, to the public domain.

Finally, at least with regards to project coordination, there was the peer pressure that the Bermuda Principles enabled: what the physical mapper Elbert Branscomb, the director of the DOE’s newly-formed Joint Genome Institute (JGI), labeled “punishment by public humiliation” in 2011 (Branscomb interview 2011).^48^ Some of the HGP’s scientists wondered if grants should be terminated if PIs were “seriously defaulting” on sharing the sequences they generated (1996 Bermuda Meeting Transcript, Session III, “Large-Scale Sequencing”). Waterston, similarly, remembers that if the data “wasn’t out there, it didn’t count” as a part of the human reference sequence (Waterston and Sulston interview 2011). In 2001, the *Science* reporter Eliot Marshall called the Bermuda Principles, “Community … spirit, with teeth” (Marshall 2001, p. 1192). Dissenters and laggards could be barred from what had become the hottest project in science, because “if they didn’t play ball then the big guns, the big sequencing centers, would just roll over them” (Morgan and Wallace interview 2012). With this embedded threat, the Bermuda Principles were no small stick. This stick would play an integral role in the policy translation of the Bermuda Principles within the HGP’s funding agencies in the years that followed, the carrot being who was, and who was not, allowed to participate in the HGP.

Yet further reasoning for daily sharing centered on patents. “We want immediate data released for more than one reason,” a Session III participant noted, “for reasons of coordination as well as” (1996 Bermuda Meeting Transcript). The speaker was cut off. Francis Collins’s notes from that session, however, reveal the reference. The interlude from earlier in the meeting had brought up the patenting elephant: “No patenting of genomic sequence & fundamental info,” Collins’s minutes from that moment read (Collins 1996 Notes). They continued—”Genes should be freely available for research work”—and from Session V, the “final session,” Collins took down the newly-drafted Bermuda Principles with key clarifications: “Aim to have all human primary genomic sequence from large scale sequencing centers freely available and in the public domain for both research and development, in order to maximize its benefit to society.” To the first white board version of the Principles, Collins had added the phrase, “human *primary* genomic” in front of “sequence” (italics mine). He was chronicling the mood in the room, which apparently favored the idea that: “Primary means in the absence of additional experimental information about function or diagnostic utility.” Moreover, the 1996 discussants elaborated: “Endorse the principle that genomic sequence, in the absence of any additional experimental information about functional or diagnostic significance, is not an appropriate subject for patent protection” (Collins 1996 Notes; also 1996 Bermuda Meeting Transcript, Session V, “Panel/Open discussion”).

The central issue was the patenting of unique short fragments of expressed genes (Cook-Deegan 1994, Chap. 19 and pp. 337–340; Cook-Deegan and Heaney 2010, pp. 400–402; Sherkow and Greely 2015, pp. 166–167; Hilgartner 2017, Chaps. 5, 7, and 8). Complementary DNAs (or cDNAs) are DNA copies of the sequences of ribonucleic acid (RNA) messengers corresponding to active genes; and expressed sequence tags (ESTs) are uniquely identifiable, truncated segments of cDNAs. In the early 1990s, cDNAs, ESTs, and materials and methods for detecting them were valuable commodities for patent claims, particularly as they could serve as targets for drugs and genetic tests. In June 1991, Mark Adams, Craig Venter, and several of their colleagues at the National Institute of Neurological Disorders and Stroke (NINDS) announced that they had sequenced ESTs from 600 proteins in the human brain (Adams et al. 1991, 1992, 1993a, b). One day before the first of these papers was submitted, Reid Adler of the NIH Office of Technology Transfer filed patent applications on the ESTs that also claimed the full-length cDNAs associated with them (Cook-Deegan 1994, Chap. 19, esp. pp. 311–312). These events sparked debates over the appropriateness and validity of certain kinds of gene patents, with *Science* stoking further controversy through news stories published as the incidents unfolded (Anderson 1991; Roberts 1991a, b).

The United States Patent and Trademark Office (USPTO) rejected the initial Venter-Adams patent applications in 1992 (Calia 1992; Fox 1992; Martinell 1992; PTO examiner to the NIH: Ptooey! 1992). But disagreements over whether the NIH should continue to pursue them, and whether it should have even applied for the patents in the first place, generated a rift between the then-NIH director Bernadine Healy and the then-NCHGR director, James Watson (Cook-Deegan 1994, esp. Chap. 20 and Epilogue). Watson hated the patent applications, at least partly because they could undermine the HGP’s value; Healy, on the other hand, supported them (Healy 1992; Thompson 1993).^49^ Watson resigned from the NCHGR, and Healy’s tenure as the NIH director expired with the Bush administration, leaving Harold Varmus in charge of the NIH and Francis Collins at the helm of the NCHGR. After careful consideration, including soliciting the advice of the patent experts Rebecca Eisenberg and Robert Merges, Varmus abandoned the NIH’s patent applications on Adams’s and Venter’s ESTs (Eisenberg and Merges 1995). A year later, Congress defunded the OTA, meaning that a report on patents and the HGP, which had been approved for publication, was nonetheless never published (*The Human Genome Project and Patenting*; Macilwain 1995). Trouble on the topic of patenting DNA, moreover, was still brewing.

From 1991 to 1993, several companies began seeking patents on cDNAs (Marshall 1994a, b; Cook-Deegan and Heaney 2010, pp. 401–402; Sherkow and Greely 2015, pp. 166–167). In the aftermath of the NIH controversy, the angel investor Wallace Steinberg persuaded Craig Venter and Mark Adams to leave the NINDS and direct the nonprofit laboratory, The Institute for Genomic Research (TIGR), which was dedicated to cDNA discovery. The company Human Genome Sciences (HGS), under CEO William Haseltine, held patent rights to some of TIGR’s results, which in turn extended exclusive rights for some uses of these cDNAs to SmithKline Beecham. Incyte Genomics and several other companies were also directing their research and development towards cDNA sequencing; and finally, a battle over patents on the *BRCA1* and *BRCA2* genes, responsible for inherited risks of breast and ovarian cancers, was smoldering between Myriad Genetics in Utah and OncorMed in Maryland, alongside parallel clashes over *BRCA2* in the UK and elsewhere (Davies and White 1996; Gold and Carbone 2010; Baldwin and Cook-Deegan 2013; Sherkow and Greely 2015, pp. 172–175; Parthasarathy 2017).^50^ No litigation on the *BRCA* patents had yet begun, but the controversy was already infamous, and the Bermuda delegates discussed it explicitly in 1996 (1996 Bermuda Meeting Transcript, Session III, “Large-Scale Sequencing”; 1996 Wellcome Trust Bermuda Meeting Report).

These patenting issues deeply affected Sulston’s, Waterston’s, and many additional laboratories well before 1996 (Cook-Deegan et al. 1996). In the early 1990s, Sulston’s laboratory had helped Mike Stratton, of the UK Cancer Research Campaign, sequence clones near *BRCA2*, convincing Stratton of daily data release even given the ongoing patent race surrounding that gene (Durbin interview 2012; Waterston to authors Feb 2017). In 1994, the then-vice president of Research at Merck, Alan Williamson, had become concerned that overly broad EST and cDNA patents—on genes of incompletely characterized biological function, for which HGS and Incyte were known to be applying—might hinder downstream development (Dickson 1994; Williamson 1999). Thickets of these broad patents might cause logjams, due to permissions required for work on many genes at once. Thus, at the 1994 meeting for the American Society of Human Genetics, with input from HUGO, Thomas Caskey, who attended the first Bermuda meeting, announced the Merck Gene Index Project (Caskey interview 2012; Morgan and Wallace interview 2012). The goal of the Gene Index was to “make data on human expressed gene sequences accessible for biological research everywhere” (Williamson 1999, p. 117). Merck’s nonprofit funding meant that the company had to prove “no special access to the resulting data” (Cook-Deegan and Heaney 2010, p. 402), so thousands of ESTs, produced by Waterston’s team in St. Louis, were to be placed in GenBank within 48 h of generation. By August 1995, Waterston had released about 163,000 sequences from 96,000 clones through the program (Waterston to authors Jan 2017). The logic was that limiting broad patents on the most fundamental HGP data would be as good for industry as it would be for science, allowing unfettered access to, and use of sequence data on, genes whose sequences were only partially known or whose function had generally not been characterized (see also, Stevens 2015c, esp. p. 484).

Each of these issues spilled into the 1996 Bermuda meeting. University, nonprofit, government, and industry stakeholders were all represented, but opposition to overly broad EST and cDNA patents was largely shared (e.g., Hilgartner 2017, pp. 137–139). Daily data release created prior art, effectively defeating the novelty and non-obviousness requirements for patents on genes of unknown function.^51^ In a 1996 internal memo, Mark Guyer of the NCHGR affirmed that the Bermuda Principles were indeed “intended to mean that the primary producers of the sequence from the Human Genome Project would not attempt to patent the sequence they generate” (Guyer to Collins 1996). Yet the 1996 Bermuda meeting transcript, particularly from the final session, reflects substantial debate about whether a formal statement—on eschewing overly-broad gene patents— should come from the HGP’s leadership, or rather from HUGO (Session V, “Panel/Open discussion”). Patents reflecting real novelty and utility, on the other hand, which the Bermuda Principles did not endanger in the US, were favored by the discussants, with one attendee asserting that: “The raw sequence of the genome, that is just the naked sequence of the genome, should be in the public domain, precisely because I think it will encourage creative and patentable activity by later parties” (1996 Bermuda Meeting Transcript, Session III, “Large-Scale Sequencing”). In 2012, the (now late) David Cox of Pfizer joked of the issue that: “the private sector is realizing that [when] patenting early information without biological understanding, it’s actually more effective and more fun just to set the money on fire” (Cox interview 2011). In 1997, HUGO did release a policy statement reiterating this nuanced Bermuda position, calling as well for patent policy changes regarding incompletely characterized cDNA and ESTs (HUGO Calls for Patent Policy Changes 1997). The HUGO statement, in conjunction with the Bermuda meetings from 1996 to 1998, then became part of a complex series of related events resulting in revised guidelines, between 1999 and 2001, for demonstrating utility and providing written descriptions for US genomic inventions (Lane 1999; Enserink 2000; Kowalski 2000; Loots 2002; Cook-Deegan and Heaney 2010, pp. 400–401). At least one US EST patent was issued in October 1998, on genes encoding kinase proteins (Robertson 1999), but the controversy surrounding the development was minimal, “no doubt because the patents were not enforced against researchers” (Cook-Deegan and Heaney 2010, p. 401).^52^

For daily-release supporters, however, a final argument remained. The HGP’s public funding was predicated primarily on constructing maps and sequences, to be shared as openly as possible as digital tools that biologists could gain access to easily and quickly. Michael Morgan elaborated in 2011 that, “a very small number of groups” were “receiving enormous sums of money to do this job. And they were doing it as a public service” (Morgan and Wallace interview 2012). One Bermuda attendee remarked in 1996 that, “early release … is the most effective way of ensuring … rapid exploitation by the whole community of workers in human biology” (1996 Bermuda Meeting Transcript, Session I, “Introductory session”). While those without reliable Internet access would certainly have missed out on many of these benefits, scientists in the larger HGP centers would not have held much of an advantage over smaller HGP groups given this equal access to the sequences.^53^ Tom Maniatis at Harvard monitored the data nightly, scanning for genes expressed in neurons (Branscomb interview 2011). He knew that the HGP had to release its data daily to everyone, but at the height of the public project he phoned Elbert Branscomb of the DOE anyways, hoping that the Branscomb group could fill in a sequence gap that was crucial to his own research.^54^ Thomas Caskey, in turn, recalls how “virtually every cell biologist I know started the morning by … looking at the latest entries,” which “enabled an entire community to be able to work on function” (Caskey interview 2012). These results validated what Aristides Patrinos of the DOE called “the religious issue” (Patrinos interview 2011), or getting “tools into the hands of the scientific community as rapidly as possible” (Marshall 2001, p. 1192). Eric Lander called this argument for the Bermuda Principles “realpolitik,” wherein those receiving the most public funding had powerful obligations to serve their scientific communities (Lander interview 2012). Public funding, by this rationale, meant publicly available resources, ideally in the form of a genome commons.

Formalized in the 1996 Bermuda meeting report, which exists in both a summary and a fuller version, the Bermuda Principles called for the attempted daily release of all HGP-funded, human DNA sequences stretching to at least one kilobase (1996 NIH Bermuda Meeting Report; 1996 Wellcome Trust Bermuda Meeting Report). Unfinished, raw “sequence assemblies” of the designated length were to be “released as soon as possible” onto individual laboratory websites, while “in some centres, assemblies of greater than 1 kb would be released automatically on a daily basis,” and any “finished annotated sequence” was to “be submitted immediately to the public databases” (1996 NIH Bermuda Meeting Report). While the 1996 handwritten Principles had not acknowledged the issue of laboratory size explicitly, the report of the 1996 meeting confirmed that one of the major goals was “to prevent such [large scale] centres [from] establishing a privileged position in the exploitation and control of human sequence information” (1996 Wellcome Trust Bermuda Meeting Report). The point about exploitation was affirmed in two April press releases after the 1996 meeting—one in the HGP newsletter *Human Genome News*, the other from the Wellcome Trust—which also advanced two additional goals that had been included in the inaugural white board draft of the Principles handwritten by John Sulston: “to encourage research and development and to maximize the benefit to society” (International Large-Scale Sequencing Meeting 1996, p. 19; also, 1996 Wellcome Trust Press Release).

The most public statement of the Bermuda Principles, however, was issued by HUGO after apparent input and revision from the 1996 Bermuda meeting participants (Steward to Bentley 1996). This statement was then reproduced on the now-archival website of Oak Ridge National Laboratory (ORNL) (Table 1; original link in International Large-Scale Sequencing Meeting 1996, p. 19). A position on gene patenting, which had been implied by Mark Guyer in his 1996 memo to Francis Collins (Guyer to Collins 1996), reflected in the meeting transcript and in Collins’s 1996 notes (1996 Bermuda Meeting Transcript, Session V, “Panel/Open discussion”; Collins 1996 Notes), and even echoed in the Wellcome Trust press release (1996 Wellcome Trust Press Release), was not included in the HUGO text, though it did, like the *Human Genome News* and Wellcome Trust statements, contain sections on participant endorsement, technical standards, and project coordination (Table 1). Additional statements related to the Principles, including by the NCHGR in April 1996 (NHGRI Policy Regarding Intellectual Property of Human Genomic Sequence 1996) and the National Research Council in 2006 (*Reaping the Benefits of Genomic and Proteomic Research*), did contain sections on patenting, with the NCHGR, reflecting the wording from Collins’s notes, explicitly discouraging patents on DNA sequences of unknown function and the NRC writing rather vaguely that, “it was … agreed that patents should not be sought” (57, Box C).^55^ While the HGP’s most formal announcement of the Bermuda Principles did occur through HUGO on the ORNL website, the most likely sources for the NRC’s 2006 account are the NCHGR’s statement on patenting and the Wellcome Trust’s press release, both from April 1996.

The policy precedents for the Bermuda Principles were diverse. From the HGP’s founding reports in 1988 and 1990, rapid and public-domain data release had always been a priority (*Mapping and Sequencing the Human Genome*; *Mapping Our Genes*; *Understanding Our Genetic Inheritance*). Yet the period from 1990 to 1992 had shown a real jumble of data-release strategies amongst the HGP’s early mapping and sequencing centers, with some centers releasing data every 40,000 base pairs, another every 100,000, and other PIs worrying about the implications of pre-publication data release for junior investigators whose careers rested on gaining credit for analytical articles (Hilgartner 2017, pp. 166–169). The NIH-DOE 6-month data-disclosure policy, from 1992, had responded to this uncertainty and also built in time for quality control and patenting, noting that:

Although it is the policy of the Human Genome Project to maximize outreach to the scientific community, it is also necessary to give investigators time to verify the accuracy of their data and to gain some scientific advantage from the effort they have invested. Furthermore, in order to assure that novel ideas and inventions are rapidly developed to the benefit of the public, intellectual property protection may be needed for some of the data and materials. (NIH, DOE Guidelines Encourage Sharing of Data, Resources 1993)

Hans Lehrach’s ICRF “reference library” policies had similarly given collaborators between 6 months and 1 year to report gene hunt data gleaned from reference library resources (Hilgartner 2004b, p. 140). Généthon scientists in France had been secretive with their data at the CSHL genome meetings, but due to their nonprofit funding had regularly reported on the genes localized and identified with their markers (Kaufmann 2004, esp. pp. 142–147; Weissenbach interview 2012).^56^ Finally, in the late 1980s, the French Center for the Study of Human Polymorphisms (Centre d’Etude du Polymorphisme Humain, CEPH) had asked investigators to use a private database for the initial housing of their data, from which the data was moved to a public space “accessible to any qualified researcher” within one year or at the time of journal publication, allowing for the “timely sharing of information while affording investigators some proprietary protection for their results” (*Mapping Our Genes*, p. 146; also, *Mapping and Sequencing the Human Genome*, pp. 79–80). The French CEPH infrastructure, however, despite reaching to North America and Africa, had been judged as too small for a scale-up to the broader HGP, and there was no enforcement mechanism.^57^

The situation was similar in Britain. The Resource Centre of the MRC Human Genome Mapping Program (HGMP), a complement to the research arm of the project focused on medically-relevant genes (Balmer 1996a, p. 536), had supplied probes, clones, and a database to grantees, but required the sharing of results, such as sequences, in return for free access to the database’s information (Balmer 1996a, b, 1998). The 1992 HGMP report read: “those who do not co-operate [by sharing] find themselves waiting for service. In principle, non-co-operators would find their registration [from the Resource Centre] withdrawn” (MRC Review 1992, p. 39). No sharing timeline, however, was specified, and during and following the EST controversy at the NIH in 1991 and 1992, the Resource Centre’s leadership had temporarily suspended access to the HGMP’s cDNA and EST sequence data (Rees 1991; Balmer 1996a, p. 546). This limitation applied both to the HGMP’s and industrial users, the latter of whom were paying customers. Much to the chagrin of several HGMP scientists, moreover, so long as the NIH had applications pending for EST patents in the US, the MRC was determined to file its own, defensive patents on ESTs (Hilgartner 2017, pp. 137–139). The tension dissipated when Harold Varmus abandoned the NIH’s patent applications in 1994, but the situation showed just how difficult genomic data-sharing policies were to articulate and to maintain, especially given the shifting stakes and stakeholders in this rapidly moving field. Nevertheless, the 1992 NCHGR-DOE 6-month data-sharing policy was clearer than any of these precedents, and due to the generosity of the timescale proposed, the NCHGR encountered minimal friction when trying to enforce it (Hilgartner 2017, pp. 169–172). The Bermuda Principles were clearer, and stricter, still.

Most attendees, including our interviewees, saw the 1996 International Strategy Meeting in Bermuda as crucial, enabling the alignment of working priorities and technological strategies (Collins interview 2012; Hillier interview 2012; Morgan and Wallace interview 2012; Rogers interview 2012; Weissenbach interview 2012). The meeting was repeated in 1997 and 1998, in the same location and at the same time of year. Alongside the Wellcome Trust, the funding for later meetings came from the NIH, the DOE, the Japanese Society for the Promotion of Science, and the UK MRC (1997 Bermuda Meeting Report; 1998 Bermuda Meeting Report). The core of the attendance remained relatively stable, though some minor shuffling did occur. Elbert Branscomb of the DOE’s Joint Genome Institute (JGI), founded in 1997, attended the second and third Bermuda meetings, as did the BMBF genomics administrator Ursula Hurtenbach (1997 Bermuda Revised Delegates List; 1998 Bermuda Programme and Provisional Delegates List). The Netherlands’ Gert-Jan van Ommen and Australia’s John Mattick joined the group in 1997 (1997 Bermuda Revised Delegates List); and the Japanese sequencing leader Yoshiyuki Sakaki as well as Fumihiko Kikuchi, from the University of Tokyo and JST, respectively, traveled from Japan in 1998 (1998 Bermuda Programme and Provisional Delegates List). Finally, in this third year of the Bermuda strategy meetings, the weather cleared, enabling the delegates to take scooter rides (1998 Meeting Photographs).

From 1996 through 1998, the details of the Bermuda Principles were modified slightly as the sequencing and assembly technologies improved and the HGP’s sequencing efforts continued apace. In 1997, the daily-release trigger was extended to 2 Kb (1997 Bermuda Meeting Report; 1997 Meeting Report Summary). The goal for minimizing gaps in the finished sequence was “set at zero [gaps],” with “only 1 gap in 250 kb of ‘finished’ sequence” deemed “allowable” and an aim “to reduce this to 1 in 1 Mb or better” articulated (1997 Meeting Report Summary).^58^ The 1997 participants, in turn, solidified the standard for what was to count as “finished” sequence: 99.99% accuracy, with no more than one error per 10 Kb, reflecting the inclinations of the attendees from 1996 (1996 Wellcome Trust Bermuda Meeting Report; 1997 Bermuda Meeting Report; 1997 Meeting Report Summary). This standard was met with general agreement—except from Craig Venter, who hated it—and was especially lauded by Francis Collins and Maynard Olson, who insisted on producing a reference genome that would be analytically useful for generations (Shreeve 2004; Venter interview 2012). Where possible, alongside details on the enzymes and clones used for sequencing, annotations from Phil Green’s *phred* (clone alignment) and *phrap* (quality score) programs were to be included with finished sequence, as it was released to the public databanks (1997 Meeting Report Summary). Moreover, a “data exchange exercise” was agreed upon for quality assessments, wherein “raw sequence data would be exchanged among sequencing centres,” the “centres would reassemble the data and identify outright discrepancies or ambiguities with reference to the sequence submitted to the database,” and “the same data sets would be sent to two centres which would hopefully engender competition to detect errors” (1997 Bermuda Meeting Report).

Finally, in 1997, the Human Sequencing and Mapping Index shifted from Susan Wallace’s office at HUGO to the NIH National Center for Biology Information (NCBI), with additional changes to where unfinished sequence was to be reported and which of the HGP’s organisms, alongside human, the Bermuda Principles were to be applied (1997 Bermuda Meeting Report; 1998 Bermuda Meeting Report; Waterston to Collins 1997). The 1997 Bermuda meeting participants were asked to delineate regions they planned to sequence, using Généthon markers, and these goals were evaluated in year-over-year confessions of actual-to-predicted sequence ratios, such that claims on the Human Sequencing and Mapping Index were allowed only “in the order of three times the sequence released by the centre in the preceding year (1997 Bermuda Meeting Report; also, 1998 Bermuda Meeting Report; 1998 Bermuda “Optimism Factor” Photographs; Branscomb interview 2011; Rogers interview 2012). Between 1997 and 1998, GenBank finally agreed to host the HGP’s unfinished data in a special division, the High Throughput Genomic Sequence Division, though the arrangement came with caveats as errors could result in shoddy follow-on research (1998 Bermuda Meeting Transcript; Bentley 1996; Ouellette and Boguski 1997; Guyer 1998; Hilgartner 2017, Chap. 6, esp. pp. 173–175). In 1998, the Bermuda meeting participants extended the Principles to mouse sequencing, an unsurprising move given Eric Lander’s support and the technological capabilities of the Whitehead Institute (1998 Bermuda Meeting Report; Lander interview 2012); and later that year, as reported by Mark Guyer, the Principles were finally applied to all of the HGP’s model organisms (Guyer 1998).

Throughout all of these changes, however, the nematode worm’s legacy endured: it was fundamental and explicit. As one Bermuda delegate declared in 1998, during the discussion about extending the Bermuda Principles to mouse: “The data release policy really hasn’t come about through the human genome, it was alive and well and really working for the nematode before it. So the advantages are not species specific but really relate to coordination and building a really effective collaborative community” (1998 Bermuda Meeting Transcript, Session VII, “Future Meetings”). In 2009, echoing the title of a 2003 book by Andrew Brown, the LMB worm biologist Sydney Brenner wrote of his participation in the HGP that: “In the beginning was the worm…” (Brown 2003; Brenner 2009, p. 413). As the worm went, so went the human genome, alongside the HGP’s other model organisms. Yet with such diverse precursors, wholesale subscription to the Bermuda Principles would presumably have been quite unlikely. How did such a large group of actors come to agree?

## Measured Consensus

In reality, not everyone did agree. After John Sulston first drafted the Bermuda Principles on the white board, a simple show of hands was requested (1996 Bermuda Meeting Transcript, Session V, “Panel/Open discussion”). The meeting report noted that “all participants voted, on a personal basis to endorse these principles,” yet “some centres may find it difficult to implement thse [sp.] principles because of legal constraints and it was agreed that all participants should be given an opportunity to comment on the final Bermuda statement before it was released” (1996 Wellcome Trust Bermuda Meeting Report). The bioinformaticist LaDeana Hillier recalls that the group sat in a U-shape, and it felt much “like the United Nations…. It was … basically going around the group and asking each … are you willing to agree?” (Hillier interview 2012). *Human Genome News* reported that the Principles “were endorsed unanimously by the attendees” (International Large-Scale Sequencing Meeting 1996, p. 19). Mark Guyer, similarly, reported that the attendees “unanimously agreed” (Guyer to Collins 1996), but Francis Collins recorded two very different sentiments: “endorsed unanimously” in one place (from Session V), and “I think we agreed” in another (from the “Interlude”) (Collins 1996 Notes). Michael Morgan remembered that “there were voices *au contraire*” (Morgan and Wallace interview 2012), and the fearlessly contrary Maynard Olson does not believe “that there was even majority support at Bermuda for these principles that were unanimously adopted” (Olson interview 2012). In 2012, Craig Venter, an avid sailor, claimed half-humorously that the 1996 Bermuda meeting had left a “bad taste” in his mouth, which “kind of destroyed Bermuda for me” (Venter interview 2012). At the most, therefore, despite the claims of unanimous endorsement, the Bermuda Principles initially garnered only a measured democratic consensus.

John Sulston and Robert Waterston certainly convinced many. André Rosenthal loved the Bermuda Principles, but perhaps he had been influenced by the time he spent in Sulston’s laboratory as a postdoctoral researcher (Rosenthal interview 2011). Jane Rogers liked releasing data as rapidly as possible, so long as users understood that the quality would vary (Rogers interview 2012). Eric Lander loved the Principles too, and he remembers having discussed the idea with Sulston and Waterston before the first Bermuda meeting (Lander interview 2012). Michael Morgan recalls that the nematode and Wellcome Trust contingents had essentially agreed unanimously, “those … represented by John and Bob and … who had been persuaded no doubt at bar room meetings and the like” (Morgan and Wallace interview 2012). The Principles were directly a product of *C. elegans*, of the sharing norms developed within this community, and as John Sulston recalled in 2011: “The two of us [he and Waterston], were … promoting the map…. [T]hen as it got bigger we pasted it on the wall…. So it [the first Bermuda meeting] was a … third session … of trying to listen to the anxieties, to reassure people, and then … distill onto the board” (Waterston and Sulston interview 2011). While once embodied in the nematode physical map at Cold Spring Harbor in 1988, the Bermuda Principles were given a more official form, on a white board in the Hamilton Princess Hotel, in 1996.

“John and Bob were recognized by the community as people who had to be listened to,” Michael Morgan explained succinctly in 2012. Whether over beer, during scooter rides, or in formal sessions, “they were extremely elegant in their advocacy of free and open data release” (Morgan and Wallace interview 2012). Drawing on the daily release of sequences from prior work on *BRCA2*, and following discussion among the trustees and the board of governors at the Wellcome Trust, the Trust adopted the Bermuda Principles relatively swiftly, and in 1996 deployed them as conditions on their HGP human sequencing grants (1996 Wellcome Trust Press Release; Rogers interview 2012). “We did have in-house questions … as to how all of this was going to work,” Michael Morgan remembers, but when “it was suggested that this data could be captured in some way by nefarious individuals who would then go ahead and patent it,” the relevant leaders at the Trust were persuaded (Morgan and Wallace interview 2012). Indeed, “there was nobody at the Sanger Centre who was going to do sequencing other than … by those principles because they were the ones who were driving the principles” (Ibid.). By May 1996, “assembled data” from contigs longer than 1 Kb was placed on the Sanger Centre’s “ftp site as it becomes available,” with daily updates and the caveat that “this data is clearly marked on our site as incomplete data, subject to error and possibly even to misidentification. People use it at their own risk,” though further curated sequences were submitted “to the public databases after they are finished and annotated in a timely manner” (Bentley to Sulston 1996). In 1998, when the Bermuda Principles were extended to all the HGP’s model organisms, the MRC confirmed that it had adopted the data-sharing policy for its nematode work, led largely by Sulston, and also for the bits of human genomics with which it remained involved (1998 Bermuda Meeting Transcript, Session VII, “Future Meetings”). China joined the HGP in 1999 (Lander et al. 2001), contingent on the compliance of its funders, the Chinese Academy of Sciences (CAS) and the Chinese Ministry of Science and Technology (MOST), with daily data release (Qiang 2004, p. 215; Morgan and Wallace interview 2012).

Roadblocks to this policy existed, however, including in the US. Maynard Olson, Mark Adams, and Craig Venter had already vehemently vocalized their reservations regarding data quality, and Francis Collins even went through an “evolutionary process” (Collins interview 2012). In the 1980s, Collins had found himself in the midst of several heated races to find disease-associated genes, including those for cystic fibrosis and neurofibromatosis. While working on the gene for Huntington’s disease, Collins’s team at the University of Michigan had broached collaboration with Sulston. “John’s very clear statement was … we have to make the data available to everybody…. And … that was the first time for me as a gene hunter in the human genetics community that this immediate access had been proposed as a requirement of a collaboration.” At first, Collins was “taken aback,” but “with a bit of pain and suffering” was convinced, especially for sequences of unknown function: “It seemed to me by about 1991, 1992 … there’s no justification for holding onto this” (Collins interview 2012). The patent battle over *BRCA2* in the UK had only strengthened Sulston’s resolve, and with leverage from the St. Louis-Merck Gene Index Project, he and Waterston convinced Mike Stratton, of the UK Cancer Research Campaign, to implement immediate release of his data associated with this gene (Durbin interview 2012; Waterston to authors Feb 2017). Sulston and Waterston’s pressure swayed Collins, who became the chief architect of the NIH’s response to the Bermuda Principles.

The NIH and the Wellcome Trust could mandate rules for data sharing, but they could not entirely suppress patents on DNA sequences: the Bayh-Dole Act stood in the way. As the deputy director of the NCHGR, Elke Jordan, told *Science* in 1996, neither the NIH nor the DOE could “legally restrict researchers or their institutions from applying for patents” (Kahn 1996, p. 1799). While the Bermuda Principles provided a powerful philosophical statement from the HGP’s leadership, therefore, they actually held little legal or policy weight. Funders did not have to agree, and neither did scientists, at least until the Wellcome Trust solidified its policies, at which point its grantees were bound to daily release if they wanted the funding. Francis Collins remembers from Bermuda in 1996 “that most of us in the room had no authority to make this decision on part of whoever it was … actually pulling the strings. I maybe did, although I’m not sure I did” (Collins interview 2012). Collins could not dictate policies that implied patenting restrictions on the recipients of NIH funding, yet he wanted the Principles’ data-sharing stipulation to be a grant condition for the HGP. As Robert Waterston recalls of that uncertain, yet pivotal, moment, Collins still “thought he could maneuver it” (Waterston and Sulston interview 2011).

On April 9, 1996, the Bermuda Principles, or at least a summarized version of them, officially appeared as a condition on the NCHGR’s pilot human sequencing grants. The policy read: “human genomic DNA sequence data … should be released as rapidly as possible … in the public domain where it will be freely available” (NHGRI Policy Regarding Intellectual Property of Human Genomic Sequence 1996). The statement acknowledged that, “grantees have the right … to retain title to subject inventions,” but it also warned, reflecting Collins’s notes (Collins 1996 notes), the transcript from the 1996 meeting (Session V, “Panel/Open discussion), and the contemporaneous press release from the Wellcome Trust (1996 Wellcome Trust Press Release), that:

Raw human genomic DNA sequence, in the absence of additional demonstrated biological information, lacks … specific utility and therefore is an inappropriate material for patent filing. NIH is concerned that patent applications on large blocks of primary genomic DNA sequence could have a chilling effect on the development of future inventions. (Ibid.)

The NIH would “monitor grantee activity … to learn whether or not attempts are being made to patent large blocks of primary … sequence” (Ibid.). Violators could have lost their funding, yet it does seem that the NCHGR’s grantees complied voluntarily, at least with the patenting provision. No Determination of Exceptional Circumstances (DEC), which could have allowed the NIH to circumvent the Bayh-Dole Act in order to prohibit incompletely characterized EST patents, was ever filed (Eisenberg 2000; Collins interview 2012).^59^ Plenty of gene hunts were, in reality, occurring in the HGP’s centers, but raw sequence patents do appear to have been avoided (Cook-Deegan and Heaney 2010, p. 404). It also appears that Collins’s political clout was strong enough to leave the pilot grants’ policy wording, which persisted into later sequencing grants (NHGRI Policy for Release and Database Deposition of Sequence Data 2000), as stated. Yet in 1996, data release within a few days or even a few weeks was still an optimistic goal for most centers (1996 NCHGR Pilot Study Press Release). In 1997, “some centres were releasing data as infrequently as quarterly” due to technical issues (1997 Bermuda Meeting Report), with similar difficulties persisting into 1998 (1998 Bermuda Meeting Transcript, Session III, “Data Release and Availability”). Even amongst the NIH’s grantees, daily data sharing was a paradigmatic standard to which only a few were able to adhere, with rapid, pre-publication sharing being the much more essential result.

The situation in the DOE was even more complicated. “I think at that point since the people we were imposing upon had all been [a] part of the decision there was not a lot of objection,” Francis Collins recalled (Collins interview 2012). This was certainly true of Elbert Branscomb, who supported the Principles (Branscomb interview 2011). But while the DOE had indeed enjoyed representation in Bermuda, some of the leaders within the agency, including Aristides Patrinos, remained skeptical of nightly sharing (Patrinos interview 2011). Many within the DOE also felt that they had not received enough credit for launching the HGP in the first place (Ibid.). Wary of inferior status, in 1998 Patrinos and others contemplated a mutually beneficial sequencing and analysis collaboration with Craig Venter’s company, Celera Genomics (Shreeve 2004, Chap. 12). But the initiative withered upon discovery by the Wellcome Trust’s leadership, and, as Thomas Caskey remembers, the “NIH held the purse strings” (Caskey interview 2012). There existed “a coalescence of views, perhaps for somewhat different reasons, between Francis Collins and John Sulston and Bob Waterston,” and because Collins “basically controlled the funding in the U.S. it was a foregone conclusion that things were going to develop along the lines he advocated” (Olson interview 2012). By the Bermuda meeting in 1997, the DOE was “trying to make its investigators adhere to the principles in the strictest interpretation” (1997 Bermuda Meeting Report). By 1998, the agency was in compliance with the Bermuda Principles (1998 Bermuda Meeting Report). “Even if we had protested,” Aristides Patrinos remembers, the NIH “could have very easily ignored us” (Patrinos interview 2011). There was no formal DOE policy statement, but as Mark Guyer reported in *Genome Research* in 1998, “most of the funding agencies engaged in supporting the Human Genome Project have adopted policies that reflect the importance of rapid dissemination of genomic sequence data” (p. 413).

In continental Europe and Japan, a much larger gulf existed between the HGP scientists and their funders. Guyer had written in 1996 that the Principles were “understood to be the sense of the attendees and that different organizations/agencies/countries might be under … constraints that might or might not allow them to adopt this as policy” (Guyer to Collins 1996). At the first Bermuda meeting, some had certainly felt blind-sided. Andreas Weller, Frank Laplace, and Ursula Hurtenbach of the German BMBF recall their “perception … that the speed of publication of results was an important issue,” but that in 1996 no international consensus had yet been reached (BMBF to KMJ 2012 and 2013). Along similar lines, Masahira Hattori from Japan remembers that most of the British and American delegates had seemed aware of the “outline of the Bermuda Rule,” hence the criticism that his team had received for not having declared the sequencing of human chromosome 21 before beginning it (Hattori to KMJ 2012). Yet not even all of the American participants knew what was going to be proposed. “We had no indication coming into that meeting that this was going to happen,” the NIH program officer Jane Peterson recalled in 2011 (Guyer and Peterson interview 2011). To make matters even knottier, the German, French, and Japanese delegates could not agree to the Principles on site, as they had to liaise further with their funders.

The problem, yet again, was commercialization. Alongside ESTs, valuable full-length cDNA patents covered genes of understood therapeutic value (for instance, insulin and growth hormone), their protein products, disease-causing sequence mutations, manipulation techniques (such as rDNA), and drugs developed from these inventions (Cook-Deegan and Heaney 2010, esp. pp. 391–396; Rasmussen 2014). In the United States, a 1-year grace period existed between when sequences were placed in the public domain and when patent applications drawing upon them could still be filed (35 U.S.C. §102(b)).^60^ Yet no such grace period, as had been discussed in Session III of the 1996 Bermuda meeting (1996 Wellcome Trust Bermuda Meeting Report), existed in many other patent jurisdictions, so outside the US the Bermuda Principles really did endanger even patents that reflected genuine novelty (Contreras 2011, pp. 86–87).^61^ The 1997 HUGO patenting statement called for the implementation of an analogous grace period in non-US patent jurisdictions, to level the playing field (HUGO Calls for Patent Policy Changes 1997), but to no avail. *Nature* reported in 1997: “[T]he US patent system gives a considerable competitive edge to US industry because it allows a ‘grace period’ under which patents can be applied for up to one year after the publication of research results” (Unterhuber 1997, p. 111). Another news feature repeated: “Raw sequence data need extensive—and time-consuming—analysis to identify genes and their functions. European patent laws exclude patents on any gene whose sequence has already been published” (Abbott 1997, p. 536). The resistance to patents on incompletely characterized gene fragments, such as on ESTs, was indeed generally shared among the attendees in Bermuda. But the threat to other kinds of commercialization, which the Bermuda Principles posed outside the US, divided the delegation, at times creating damaging friction.

The BMBF sent delegates to all three Bermuda meetings, but its policies were the most overtly incompatible with the Bermuda Principles. On May 20, 1995, the BMBF, the DFG (Deutsche Forschungsgemeinschaft), and the Max-Planck Society, three predominant research patrons in Germany, published the funding concept that launched the German Human Genome Project (DHGP), aimed at “the development and strengthening of the German scientific community in the area of human genome research at large” (BMBF to KMJ 2012 and 2013).^62^ In 1996 and 1997, under the administration of Frank Laplace, the BMBF awarded 60 peer-reviewed grants, three of which constituted the German Genomic DNA Sequencing Consortium led by André Rosenthal, Hans Lehrach, and Helmut Blöcker.^63^ The main goal of the consortium was “to establish a national research network (DHGP) for the identification of medically relevant genes and … analysis of their structure, function, and regulation.” A second purpose was “to increase knowledge on the etiology and pathology of diseases with high socioeconomic relevance,” and a third was the “development of new products and services by the German pharmaceutical and biotech” industries (BMBF to KMJ 2012 and 2013).

Early on, the BMBF received the criticism “that [German] industry had declined to contribute to the [national] human genome programme” (Unterhuber 1997, p. 111). Consequently, the ministry invited the firms Asta Medica, Bayer, BASF, Boehringer Ingelheim, Boehringer Mannheim, Hoechst, Merck KgaA (separate from the US Merck), and Schering into an industry consortium, the Förderverein Humangenomforschung (Unterhuber 1997, p. 111). The mandate of the consortium was “the transfer of scientific results into novel industrial applications” (BMBF to KMJ 2012 and 2013). The consortium funded a patenting and licensing agency in return for privileged access to the German HGP’s data, located in a Primary Database housed at the Resource Center (RZPD) at the MPI in Berlin.^64^ The development resembled the British HGMP and the French CEPH arrangements from before 1995, especially the “reference library” that Hans Lehrach had run at the ICRF in London. All of the RZPD’s users, including the DHGP centers, were obliged to deposit their new data, including DNA sequences, into the database. The data’s producers could request up to 6 months of confidentiality, after which 3 months’ privileged access was granted to the Förderverein “for bilateral co-operations … or for licensing negotiations with the owners of the data” (BMBF to KMJ 2012 and 2013). The Förderverein funded no sequencing, but got a head start in commercialization. After the period of privileged industry access, all data were released to the public domain.

The BMBF policy, not surprisingly, raised eyebrows in Bermuda, though it is unclear if the dissenters understood the complete details of the arrangement.^65^ “There is discussion on how to get industry money into the entire project and then … industry is likely to request … a few months in which they can access the data,” the 1996 meeting transcript read (1996 Bermuda Meeting Transcript, Session V, “Panel/Open discussion”). The speaker may or may not have understood, though, that German companies were not funding the DHGP, but rather helping in efforts towards downstream development. Preceding the 1996 meeting, the BMBF had thought its data-sharing arrangement was “quite in line with the time frames discussed in the scientific community” (BMBF to KMJ 2012 and 2013). It was, indeed, in accordance with general standards in genetics and genomics, yet several voices in Bermuda deemed the plan intolerable (1997 Bermuda Meeting Report; 1997 Bermuda Meeting Transcript; Unterhuber 1997, p. 111). “[I]nternational concern with the German policy was made known in the strongest possible terms,” the 1997 Bermuda meeting report read. It “could both endanger the early data release policies in other countries and … lead to duplicate (and therefore uneconomic) sequencing,” so “the scientific community should exert its influence to prevent this.” The DHGP policy was up for review after one year anyways, and the members of the sequencing consortium still shared their data amongst one another for quality control (1997 Bermuda Meeting Report). The 1997 Bermuda delegates, however, were speaking past one another, generating friction within the German genomics community and beyond.

André Rosenthal vehemently defended daily data release on German television and the radio (Rosenthal interview 2011). After the second Bermuda meeting, in March 1997, he informed Michael Morgan of a new working arrangement he had broached with Schering, which would divide his time between industrial research and the DHGP (Rosenthal to Morgan 1997). As a half-time endeavor, alongside his HGP sequencing efforts in Jena, Rosenthal would direct an industry-funded Institute for Genome Research, to be located near Hans Lehrach’s laboratory at the MPI in Berlin. Yet he promised Michael Morgan of the Wellcome Trust that there would be no conflict of interest, asserting that, “I am personally committed” to the “instant release of genomic sequence data to public databases with no delay,” and that he was “the only scientist … funded by the German BMBF who repeatedly criticized the intention of the … BMBF to submit genomic sequence data into a … database which is accessible to a selection of German Pharmaceutical Companies” (Ibid.). Rosenthal recommended that the major British and American funders liaise closely with the BMBF to help promote a shift in the privileged-reading policy, suggesting a letter to *Nature*, and one 1997 Bermuda delegate even “proposed that there should be lobbying to put the data release policy on the agenda for the next G7 summit” (Kent to Collins 1997). The BMBF began an informal moratorium on the privileged database access in March of that year (BMBF to KMJ 2012 and 2013). Meanwhile, on March 21, a heavy-handed letter to Frank Laplace from Francis Collins and Aristides Patrinos arrived, which cautioned that:

It is essential that all of the participants in the international Human Genome Program have the same policy with regard to the release of human DNA sequence data. We urge the BMBF to reconsider its decision and bring its policies into line with those of the other participants…. It is our opinion that, by definition, continuation of such restrictions on the immediate availability of the sequence data would mean that the German program is not, in fact, participating in the Human Genome Project as it is defined and practiced in the rest of the world. (Collins and Patrinos to Laplace 1997)

The warning of HGP exclusion was coupled to one from André Rosenthal, who “threatened to refuse his approved ministry [DHGP] grant of nearly DM20 million (US$12 million) to set up his sequencing laboratory unless he was allowed to publish his data immediately on the Internet” (Abbott 1997, p. 536). To ease the escalating tensions, the BMBF held a summit in Bonn on May 26. While “all [the] important players from the US and the UK were invited,” however, none of them came (BMBF to KMJ 2012 and 2013).

By the 1998 Bermuda meeting, the BMBF’s privileged data-access policy was formally lifted. It remained in effect though 2004 for the other DHGP laboratories, but it no longer applied to the DNA Sequencing Consortium (BMBF to KMJ 2012 and 2013). *Nature* had reported a few months earlier that, “the threat by geneticists in the United States and Britain to exclude German scientists from international collaborations in the Human Genome Project, and to block their access to vital biological material, outweighed the advantage to industry” the Förderverein’s privileged data-reading time presumably conferred (Abbott 1997, p. 536). This *Nature* report fueled one misconception—that German scientists could somehow have been blocked from data in the public domain—but it did accurately communicate a more viable threat, that Germany could have been excluded from the HGP. Indeed, “the major driving force” behind the BMBF’s policy shift by the 1998 Bermuda meeting was “to hold off distress from the German scientists and to keep them an equal member of the human genome research community” (BMBF to KMJ 2012 and 2013). The transcript of the 1998 meeting indicated that “the [German] Genomic Consortium can now fully adhere to the Bermuda principles,” a notice that was met with applause and soon confirmed by the meeting report (1998 Bermuda Meeting Transcript, Session III, “Data Release and Availability”; also, 1998 Bermuda Meeting Report). In 2013, the BMBF wrote that it had been unable to trace any patents directly to the short-lived delay-for-review data access policy, despite this having been the policy’s original, putative objective (BMBF to KMJ 2012 and 2013).

The situation in Japan was similar. The JST had contracted with each HGP sequencing center beginning in 1995, when the agency had explicitly explained its data-release policies to grantees (Sakaki interview 2011). The sequencing program included the Advanced Life Sciences Information Systems (ALIS) Project, the goal of which had been a “*human genome database that will provide an efficient source of information for researchers after the human genome has been sequenced*” (Hirakawa et al. 1997, p. 278, italics in original). The STA had no hard policy on unfinished sequences, though (according to Masahira Hattori’s recollections) finished sequences were to be submitted to the ALIS master database and released to the public domain every 3 months so that quality control checks could be performed (Hattori to KMJ 2012). In 1997, some of the Japanese investigators were releasing their unfinished data to laboratory websites (1997 Bermuda Meeting Report). By 1998, the STA’s grantees had up to 3 months to report their finished sequences to the database—a window that was down from six months, in 1997 (1997 Bermuda Meeting Report)—thereby allowing a potential delay of up to 6 months (down from nine, after the STA’s quality control checks) before public release (1998 Bermuda Meeting Transcript, Session III, “Data Release and Availability”). The 1998 Bermuda meeting report confirmed that the “Science and Technology Corporation does not restrict or require immediate data release, however finished data has to be released by the JST” (1998 Bermuda Meeting Report). The Japanese human sequencing leader, Yoshiyuki Sakaki, was releasing his unfinished data in agreement with the Bermuda Principles, and so was Keio University, once again according to Hattori (Hattori to KMJ 2012). The Tokai University and Japanese Foundation for Cancer Research teams, on the other hand, may have been delaying unfinished data release.^66^

While the practices for unfinished data varied in Japan, everyone was bound by the STA rules for finished sequences, and “in a delicate position to get support to continue the project” (Sakaki interview 2011). Despite the stated reason of quality control for the delayed release of data from ALIS, there was also the suspicion that the STA was seeking gene patents, doing “whatever it is they do with it [the data] and we’re still not sure what that is” before public release (1998 Bermuda Meeting Transcript, Session III, “Data Release and Availability”). In 2000, Steven Collins and Hikoji Wakoh reported on several Japanese reforms, modeled on the US Bayh-Dole Act, which let university investigators retain title to publicly funded inventions (Collins and Wakoh 2000). If the STA was indeed seeking patents, it was not alone, and it was in fact well in line with the global trend towards commercialization in molecular biology. Nevertheless, the STA’s delegates could never accept the Bermuda Principles directly on site. In 1996, Fumihiko Kikuchi had had to return to Japan to consult with the relevant government administrators. In 1998, so did Sakaki, who was surprised at the extension of the Bermuda Principles to mouse in that year. “It was beyond my responsibility,” he recalled in 2011, to accept this proposition, even though he seems to have agreed with it (Sakaki interview 2011).

In March of 1998, following that year’s Bermuda meeting, Francis Collins, Aristides Patrinos, and Michael Morgan sent stern a letter to Ken-ichi Matsubara of Osaka University, echoing the one sent to the BMBF in 1997 (Collins, Patrinos, and Morgan to Matsubara 1998). Matsubara was not an STA sequencing PI, but he was no stranger to genomics, nor to the heated data-sharing politics so closely associated with the field. He had long been a leader in molecular biology, completing his postdoctoral fellowships at Harvard and Stanford and, by the late 1980s, playing the anchor role in bringing the Monbusho (the Japanese Ministry of Education, Science, and Culture) into the fold of funding genomics in Japan (Cook-Deegan 1994, Chap. 15, pp. 218–223). In 1989, James Watson had even ignited a controversy when, following a visit from Matsubara to the US, he had refused a reciprocal visit to Japan on the grounds that the Japanese government was being stingy with its funds for genome research. Watson threatened that the US would “make access to databases and research materials difficult” for Japan (Ibid., pp. 219–220); and 10 years later, with Matsubara now at the helm of several non-sequencing projects in the Japanese HGP (1996 Wellcome Trust Bermuda Meeting Report), history repeated itself.

“Only Professor Sakaki appears to be adhering to … rapid release of unfinished DNA sequence contigs of 2 kb or more,” the letter from Collins, Patrinos, and Morgan to Matsubara described of the Japanese HGP in 1998; “the other three centers apparently do not do this.”^67^ “Second,” the letter continued,

it seems that JST still insists on reviewing completed sequence contigs (which are sent to them every 3 months) for an undefined period of time before permitting their deposit into the international DDBJ database. Thus, completed sequence contigs may often be inaccessible for many months. We have been unsuccessful in our efforts to learn the reasons for the JST review of the data. (Collins, Patrinos, and Morgan to Matsubara 1998)

The 1998 Bermuda Meeting Report had noted that, “the requirement to release finished data via the JST database … resulted in a delay of approximately 3 months in the release of data,” and the 1997 Meeting Report had confirmed the JST’s checking period of 3 months. This was hardly the “undefined period of time” before public release that the warning letter above reported, and the 1998 meeting transcript indicated that the JST did not intend to unduly delay its release of finished data (1998 Bermuda Meeting Transcript, Session III, “Data Release and Availability”). Nevertheless, the potentially six-month delay, incorporating the time that investigators had to report their sequences, was consistent with the assertion that “completed sequence contigs may often be inaccessible for many months” (Collins, Patrinos, and Morgan to Matsubara 1998). “It would be unfortunate,” the letter from Collins, Patrinos, and Morgan elaborated, “if the current situation of less than complete compliance [with the Bermuda Principles] were to be perpetuated in Japan, as the consequences for international cooperation and good will in this noble enterprise might well be significant” (Collins, Patrinos, and Morgan to Matsubara 1998). There was no explicit threat of HGP exclusion akin to the one that had been levied against the BMBF in 1997, but perhaps that warning was implied.

As the first round of STA sequencing grants was ending, in January 1998, *Nature* reported a massive “human genome” funding boost in Japan, up 162.5% from 1997 despite a relatively austere budget overall (Saegusa 1998a, p. 111). In March, the journal reported on a new Japanese HGP, the Biomolecular Research Programme, which was to be backed jointly by the STA and the RIKEN Institute of Physical and Chemical Research (Saegusa 1998b). The Programme would focus on sequencing and protein analysis, more specifically on mouse cDNAs and human chromosome 21. Yoshiyuki Sakaki would direct the entire Programme beginning in October 1998, honoring the Bermuda Principles for finished and unfinished data, and the Tokai University and Japanese Foundation for Cancer Research Teams would finish out their grants under the STA’s original rules (Hattori to KMJ 2012). Japanese compliance with the Bermuda Principles, as in Germany, thus came not through a new policy on data release, but rather through the selective neglect of an old one. “We were not in the position to be able to request … some written agreement,” Sakaki recalled in 2011. The STA had “allowed us to release the data as a member of the international consortium,” taking a “‘non-written agreement’ on mutual reliability” (Sakaki interview 2011). No formal policy reflecting the Bermuda Principles was adopted in Japan; and this fuzzy approach, to further complicate matters, spilled into France.

Two reports, both authored by Jean-Marc Egly, launched the French human genome program (1995 French HGP Planning Report; 1996 French HGP Planning Report). The central policy question was whether a single sequencing center, akin to the situation in the UK, was preferable over distributed centers, such as in the American HGP. The 1995 planning report recommended the latter option, at which point the experienced French mapper, Jean Weissenbach, suggested that Egly attend the first Bermuda meeting (Weissenbach interview 2012). The second planning report, published after the meeting, reversed the conclusion from the original report, recommending the centralized national sequencing center that became Genoscope. Both of the plans, however, emphasized data sharing. As the 1995 report stated: “A very large sequencing center must supply databases to a very significant level and in so doing gain international gratitude for the effort made in France” (1995 French HGP Planning Report). The 1996 report noted, similarly, that, “the product of a sequencing center is sequencing data, which are then made into public data” (1996 French HGP Planning Report). Following the Bermuda discussion in 1996, Jean-Marc Egly reported the immediate data-release policy of the HGP to the French basic research agency, the CNRS (Weissenbach interview 2012). Weissenbach recalled in 2012 that the CNRS immediately acquiesced to the Bermuda Principles, but the actual implementation was more complicated. The main problem, once again, was patenting.

Genoscope began sequencing genomes in 1997. As the Bermuda report from that year described, two project types were already ongoing in France: the in-house, completed with only Genoscope’s researchers, and the external, which were collaborative projects with other French laboratories (1997 Bermuda Meeting Report). Under Jean Weissenbach’s leadership, all of the in-house projects would immediately adhere to the Bermuda Principles, but the policies for external collaborations, not entirely under Weissenbach’s control, would be much harder to dictate. In June 1997, *Nature* reported of Genoscope that “a decision about whether to place all its data on the Internet or to grant French industry privileged access is due this month” (Abbott 1997, p. 536). The BMBF uproar was still fresh, and Weissenbach feared comparable international censure if an agreement similar to the German one were to be brokered with French companies. The 1998 Bermuda meeting transcript, however, showed little change from the previous year (1998 Bermuda Meeting Transcript, Session III, “Data Release and Availability”). The meeting report elaborated that Genoscope’s in-house sequences of human chromosomes 3 and 14 were to be released daily, but “no such requirement was being made for collaborative projects” (1998 Bermuda Meeting Report). None of Genoscope’s collaborations at the time involved the human genome, but concern over the French national sequencing center’s data-sharing policies persisted, especially given the extension of the Bermuda Principles to all of the HGP’s organisms in 1998.

French genome policy, as in other developed nations, promoted rapid commercialization (Rabinow 1999). Genoscope was part of a new genomics research park, aptly called Genopole, in Evry, which was home to several institutions building on the strong French traditions in genetic and physical mapping (Kaufmann 2004). In April 1998, Genoscope planned to sequence between 5 and 10% of the human genome, alongside the genome of the archaebacterium, *Pyrococcus abysii* (Balter 1998, p. 30). The French sequencing center was also working on the flowering plant, *Arabidopsis*, and the puffer fish, both of which were to be treated as in-house projects and governed by the Bermuda Principles. More broadly, however, daily data release was always a “sticky point because the French research ministry wants its biomedical research activities to pay off its public investment by generating patents” (Balter 1998, p. 31). The resulting policy compromise thus resembled that in Japan and Germany and was, rather than a newly adopted HGP data-sharing policy, akin to de facto Bermuda compliance. “We are not as strong as the Sanger Centre, which can impose its own rules on its collaborators,” Weissenbach admitted (Ibid.). Indeed, despite heavy pressure from John Sulston and the Sanger Centre, Weissenbach could never promise daily data release for the several external projects with which his center was involved. Aside from work on the mouse map, however, Genoscope was hardly ever connected to external projects involving the HGP’s official model organisms. Weissenbach’s teams contributed to the rice map, organized by the Rice Genome Project (International Rice Genome Sequencing Project 2005; Halliday 2010), the genome of the flowering plant, *Medicago truncatula*, managed by Bruce Roe at the University of Oklahoma (Young et al. 2011), analyses of the genome of the puffer fish, *Tetraodon nigroviridis* (Jaillon et al. 2004), and minimally to the rat genome (Weissenbach to KMJ 2017). Yet even for in-house projects, daily data release at Genoscope “was not a case of formal policy…. I think it was easier to just follow the rules” (Weissenbach interview 2012).

Weissenbach never received a warning letter. In 1999, the French initiative GenHomme launched “to generate economic benefits from the post-sequencing phase of the human genome project” (Butler 1999, p. 569). One of GenHomme’s major strategies was the restricted release of annotated sequences, “in which the structure[s] and function[s] of genes have been identified” (Ibid.). Yet Jean Weissenbach strongly opposed the policy, as did David Bentley of the Sanger Centre. Presumably, the French genome program had always observed the Bermuda Principles to the fullest extent that it could, in order to avoid the scorn that was levied at Germany and Japan. By the summer of 1998, moreover, the HGP’s leadership was otherwise preoccupied, this time with new developments concerning Craig Venter and Celera Genomics.

## Celera and Community Resource Projects

The Celera Genomics bombshell burst in May 1998 (Shreeve 2004). Mark Adams of TIGR had attended the third Bermuda meeting, but Francis Collins remembers him being slow on delivering some of his promised sequences (Collins interview 2012; Venter interview 2012). Then Collins and Michael Hunkapiller, president of the Applied Biosystems (ABI) section of Perkin-Elmer, shared a plane flight, during which Hunkapiller dished some of the details of his, Adams’s, and Venter’s plans to form a sequencing company. The news became public through an exclusive in *The New York Times*, in which the journalist Nicholas Wade reported that a new company, later named Celera Genomics, would “race” the HGP to complete the human genome sequence at a vastly lower cost (Wade 1998c; also, 1998a, b).^68^ The vehicles powering this effort would be the ABI Prism 3700s, recently unveiled sequencing marvels which—by relying on capillary tubes instead of slab gels for the fluorescent detection of DNA nucleotides—could operate around the clock (García-Sancho 2015, pt 3).

The new company, Celera, was to utilize a markedly different sequencing strategy from that of the HGP. Following projects by Frederick Sanger on bacteriophage and other viruses in the 1980s, Whole Genome Shotgun (WGS) sequencing was tested once again in the bacterium *Haemophilus influenzae* in 1995 (Fleischmann et al. 1995; Venter interview 2012). This *Science* paper had implied that the alternative method of sequencing might be extended to even larger genomes, and the genome of the fruit fly, *Drosophila*, yet another test case, was completed by 2000 (Adams et al. 2000). WGS sequencing had originally been debated, but rejected, as a strategy by the HGP’s leadership, due mostly to concerns about data quality and continuity (Green 1997; Weber and Myers 1997; Shreeve 2004, p. 22; Hilgartner 2017, Chap. 7). Accordingly, it was a topic discussed in Bermuda, during the HGP’s sequencing strategy meetings (1997 Bermuda Meeting Report; Rogers interview 2012). WGS sequencing skipped the genetic and physical mapping steps, relying predominantly on computer algorithms to assemble fragmented genomes using overlapping pieces (Venter et al. 1998; Bostanci 2004, Figures 8.2–8.3; Hilgartner 2017, Figures 7.1–7.2). Venter’s goals for Celera were the sequencing, identification, and patenting of novel drug targets, including “new genes that are key for pharmaceutical development, new hormones that could become pharmaceutics” and tools for understanding diseases (*How Private Sector Developments Affect the Government Program*, p. 77). Venter even planned on “patenting cDNA’s in a limited number for new, exciting discoveries that we make with the genome,” and after having disagreed with the publicly funded HGP’s technical and organizational strategies for years, considered Celera as an opportunity to profit while also doing science his own way (Ibid.).^69^ Venter felt that the yeast genome could have been the first one completed had the effort not been so fragmented, and he argued forcefully that the HGP was suffering from the same defects.

The response to the Celera announcement was profound. The Wellcome Trust redoubled its financial commitment to the HGP (Wade 1998a), ensuring that the Bermuda Principles were preserved even though Celera could, and did, make use of the public project’s data (Shreeve 2004, Chap. 14). The HGP’s sequencing efforts were narrowed to just five centers (nicknamed the “G5”) in 1999: the Sanger Centre in the UK, the Washington University Genome Sequencing Center in St. Louis, the Baylor College of Medicine, the DOE’s Joint Genome Institute (JGI), and the Whitehead Institute of MIT (later the Broad Institute of MIT and Harvard), still led by Eric Lander (Pennisi 1998, 1999; Waterston and Sulston 1998; Sulston and Ferry 2002; Shreeve 2004; Venter 2007; McElheny 2010). The Bermuda-style International Strategy Meetings continued, complete with their peer-pressured enforcement for daily data release, but at much less regular intervals and in different locations (for instance, 4th International Strategy Meeting 1999; also, Branscomb interview 2011; Morgan and Wallace interview 2012). The organization of the project transitioned from meetings to phone calls, often held weekly. The goal of having a finished sequence was moved forward, from 2005 to 2003, and immediate work was redirected towards a draft human genome, to be completed by 2001, which would maintain high accuracy (still around 99.99%) but allow for lower coverage and more gaps (ultimately including only 94% of the genome) (Wadman 1998; Lander et al. 2001).^70^ As was the case with Celera, the HGP moved to the ABI Prism 3700 sequencers, such that these machines now powered both sides of the sequencing race (García-Sancho 2015, pt 3). Finally, the new National Human Genome Research Institute (NHGRI), upgraded to an NIH Institute from its Center status in 1997 (NCHGR Becomes NIH Institute 1997), extended the Bermuda Principles to WGS data in 2000 (NHGRI Policy for Release and Database Deposition of Sequence Data 2000), another notch in the series of revisions to the Principles that had occurred since 1996.

With the news that a private company intended to produce a human reference sequence earlier, and more cheaply, than the public Human Genome Project, there were open calls to end federal funding (Dickson and Wadman 1998). There is some evidence that the Bermuda Principles themselves had endangered the project once before, as a 1996 Bermuda delegate had referenced, during the meeting’s final session, “at least one person running for high public office in the US, who, were he to read this [data-sharing] statement, would conclude that the publicly funded genome project should stop” (1996 Bermuda Meeting Transcript, Session V, “Panel/Open discussion”). Giving away costly genomic data on a daily basis, with the bill footed largely by taxpayers, had never been an entirely popular tactic; and, according to Elbert Branscomb’s recollections, some of the political support for the Bermuda Principles in the US had even been initially contingent upon securing compliance from foreign governments, ensuring that all of the HGP’s participants were playing by the same rules (Branscomb interview 2011). But the threat to the HGP, and to the Bermuda Principles in turn, grew more genuine on June 16 and 17, 1998, at a hearing before the Committee on Science of the US House of Representatives. The purpose of the hearing was to assess the continuing utility of the public HGP in the face of the Celera surprise, guided by four penetrating questions:

[A]re the goals of this [private] initiative realistic or just an optimistic vision? Will this private sector initiative duplicate the federal program and make it redundant or is it another approach that can complement the federal program and make it stronger? Is the pace and the cost of the federal program increased by the bureaucratic nature of any federal program or does the timetable and cost reflect what is necessary to do a thorough job? And will the federal program utilize the latest technology described in the private sector announcement? (*How Private Sector Developments Affect the Government Program*, pp. 1–2)

Given this challenge, the HGP’s leaders now had to defend their project. They faced a major scale-back in goals and symbolic significance, and perhaps a wholesale shutdown. At a meeting of the competing sides at CSHL on May 13, 1998, Venter had suggested to Francis Collins and the broader HGP that: “If we can get the human genome done,” then “you could concentrate on the mouse genome. Everybody wins” (Shreeve 2004, p. 51). Few were amused.

In defense of the public project, the HGP’s leadership brought up major problems with Celera’s approach in terms of data quality and access. Celera relied on relatively unproven machines (though the HGP soon came to adopt the very same ones), and the WGS strategy would leave gaps that only the public effort could close (*How Private Sector Developments Affect the Government Program*, pp. 12, 22; Wade 1998b). Francis Collins argued in the 1998 hearing that “the publicly-funded effort is probably the only part of this enterprise that’s absolutely dedicated to obtaining the completely contiguous, highly-accurate, close-all-the-gaps” product, and “I think we need to take that … seriously” (*How Private Sector Developments Affect the Government Program*, pp. 75–76). While the HGP’s daily data-release policy had indeed induced its own share of friction about data quality in 1996, in the face of the Celera race, even Maynard Olson worried more about the gaps that Venter’s WGS sequencing would leave (Olson 2002). These gaps could create problems for downstream research, and in the 1998 hearing, Olson criticized the WGS approach sharply for this reason (*How Private Sector Developments Affect the Government Program*, p. 23). In 2002, Olson also recalled that Congressman Vernon Ehlers, who held a PhD in physics, wanted to see how both the public and private projects proceeded, in order to assess “how the Celera *versus* public-sector competition played out” (Olson 2002, pp. 937–938). Ehlers only desired this outcome, however, if the HGP’s higher-quality sequences were also guaranteed. All of these quality concerns were moot, moreover, if the human reference sequence were not to become rapidly available, in the public domain, as a genome commons to be used by biologists for generations. Venter’s team had proposed to release its data quarterly, allowing for patents, and planned only limited access to its sequences through costly database subscriptions. As a direct foil to Venter’s approach, the HGP’s leadership called daily data sharing to their defense, openly invoking the Bermuda Principles.

Francis Collins in particular argued for the necessity of a genome commons, transforming the public HGP from possibly superfluous, given the private sector’s efforts, to essential. The HGP leadership’s rhetorical strategy ranged from discouraging patenting, to expediting downstream research, to loftier and more general claims about publicly funded science conducted for the greater good (Hilgartner 2017, Tables 7.1 and 7.2). “While quarterly data release,” the proposal offered by Celera, “is commendable,” Francis Collins noted in his advance statement for the 1998 House hearing, “the plan is not as strong as the standards established by the international sequencing community which require [the] release of data within 24 h and discourage patenting” (*How Private Sector Developments Affect the Government Program*, p. 23). Celera’s quarterly sharing promises, moreover, were in no way binding, meaning that researchers outside the company might be left with either a genome behind a pay wall, or no genome at all, if the Celera effort did prove to be unsuccessful. “Daily release … was arrived at because of the great interest in the scientific community in gaining access to this highly valuable information,” Francis Collins explained, and “any delay [in data release] can result in wasted effort in research” (Ibid.). He also warned that “if business demands were to change or personnel were to change or the [Celera] stockholders were to decide it’s not such a good thing to be giving this all away anymore, one would not want to see a circumstance where the publicly-funded effort … dropped the ball. We don’t intend to drop the ball” (p. 79).

These arguments employed daily data sharing to emphasize the differences between the HGP and Celera. They relied on the relative speeds (and mechanisms) by which data would be shared in the public domain, convincing the members of the House Committee on Science that both the public and private efforts should continue. The science writer James Shreeve observed that in this turning point, the most important thing, for Collins in particular, “was about community, about rules that applied to all, about the sacrifice of individual motives for the collective good.

It was even a bit about God” (Shreeve 2004, p. 124).^71^ “Public funding of the HGP,” the project’s 1998 progress report echoed, “is predicated on the belief that public availability of the human sequence at the earliest possible time will lead to the greatest public good” (Collins 1998, p. 685). The HGP survived and daily sharing had come to its rescue, standing in as an argument for the value of the public project even though the Bermuda Principles had encountered much resistance in the years before. The Principles soon helped the project in a more functional sense, too, keeping investigators at maximum efficiency by requiring that everyone “sequence blind” and not “pay attention to the data,” releasing their sequences daily “even if one of the juiciest genes in the genome has just come out” (Branscomb interview 2011). Enriching the religious metaphors so closely associated with this aspect of the HGP, Elbert Branscomb judged in 2011 that the Bermuda Principles, especially in conjunction with the eminent threat from Celera, offered an excellent solution to the “puritanical problem. How do you keep people disciplined? When they’re digging gold how do you keep them from stealing any of the gold,” and then “walking off with it?” (Ibid.).

The contrasting positions between the HGP and Celera, however, underscored by frenzied press coverage in *The New York Times*, were largely overblown, particularly regarding patents. Francis Collins noted in the 1998 House hearing that “to our knowledge, none of the genome centers are filing for intellectual property protection. They just don’t have time and their goal is … to get the data out there” (*How Private Sector Developments Affect the Government Program*, p. 82). Similarly, Aristides Patrinos of the DOE wrote in his answers to the post-hearing questions that “we are not, at the NIH, allowed to deny our grantees the opportunity to file for intellectual property rights on things they discover with NIH funds, because of the Bayh-Dole Act,” but “as a practical matter … the publicly supported sequencing community has agreed to a 24 h data release policy, and we are not aware that there have been any patent filings” (p. 94). Collins and Patrinos, however, were likely referring to patents on uncharacterized ESTs, the only kinds of patents that the Bermuda Principles—at least in the United States—really threatened. The HGP was actually generating plenty of patents on genetic material, including in its own laboratories, and stimulating much downstream patenting as well (Cook-Deegan and Heaney 2010, p. 404; Tripp and Grueber 2011; *National Bioeconomy Blueprint*; Gitlin 2013; Hayden 2014; How to Get Ahead 2014; Rasmussen 2014; Sherkow and Greely 2015; Rai and Sherkow 2016). In 2012, Maynard Olson recalled that the public project’s “strategy for sequencing … would have been the worst possible technical strategy for preempting gene patenting if that was actually our goal because we weren’t [preferentially] targeting genes” (Olson interview 2012). Craig Venter’s more cynical interpretation was that the HGP’s method of data “dumping,” his description of the Bermuda Principles, actually “sped up patenting of the genome” (Venter interview 2012).

The blurriness between the public and private efforts, moreover, went even farther than this. In his own set of post-hearing questions and answers, Craig Venter claimed in 1998 that “we [at Celera] are not planning to seek patents on broad sets of ESTs similar to what was done at NIH [in 1991 and 1992],” but rather they hoped “to fully characterize a small subset of key genes for which we will seek to identify and understand their biological significance” (*How Private Sector Developments Affect the Government Program*, p. 99). Similar dialogues had taken place during the HGP’s early planning sessions, in the Bermuda meetings, and afterwards, especially in reference to the biologically and commercially valuable research the HGP would ideally fuel downstream. Aristides Patrinos noted in his own post-hearing questions that, “the sequence itself is publicly accessible,” so “future investigators, who figure out the value of a particular gene sequence and/or turn that … information into a pharmaceutical or a new diagnostic, may decide they have added enough value to meet the patent criteria … and file for a patent” (p. 94). While the arguments defending the HGP against Celera indeed proved successful, therefore, they did create some misconceptions. Rhetoric related to the genome commons and commercialization in 1998 largely (though with exceptions) lacked the nuance of previous deliberations, including those in Bermuda, when the HGP’s leadership had hardly proven itself as wholly opposed to genomic patenting and commercialization.

These misconceptions, in turn, engendered lingering consequences. On March 14, 2000, at the National Medal of Science and Technology Awards Ceremony, US President Bill Clinton and UK Prime Minister Tony Blair issued a joint statement supporting the “rapid release of human genome sequence data,” following discussions of a possible extension of the Bermuda agreement to additional government agencies in the US and the UK (Hencke et al. 1999; Marshall 2000a, p. 1903). Drafts of this statement, and the associated correspondence, indicated that President Clinton’s science advisors anticipated a potential misunderstanding, noting for instance that, “the statement refers to ‘raw fundamental data’ regarding the human genome, which generally does not qualify for patent protection because there is no immediate use of this information other than to use it for further research. Wider distribution of this information will stimulate inventions that can be patented” (Joint Statement on the Human Genome 2000). Indeed, in a hiccup exacerbated by the White House press secretary’s own misunderstanding, the US markets responded to the March 14 declaration as if the leaders had denounced all patents on human genetic material, not just some, resulting in a colossal sell-off of biotechnology stocks that led to a steep drop in the NASDAQ biotechnology index, at the time its second-largest daily loss (Berenson and Wade 2000; Marshall 2000b). To make matters worse, the fiasco came on the heels of a similar mix-up in the UK, when an *article in* The *Guardian* had “inaccurately portrayed” the 1997 Bermuda sharing statement—which was a re-affirmation of the original, 1996 Bermuda agreement with only minimal technical amendments—as being “designed to preclude all gene patents” (quotes from Lane 1999; also, Hencke et al. 1999). As was predicted in Bermuda, however, the HGP’s daily data-sharing strategy actually proved better for innovation than did Celera’s approach. In 2013, the economist Heidi Williams compared genomic sequences initially produced under the Bermuda Principles to those subjected to Celera’s proprietary database constraints (Williams 2013). Williams found 20–30% increases in scientific citations to the genes sequenced and released under the Bermuda Principles, with comparable upticks for the development of diagnostics for conditions related to these genes. The daily-sharing framework, perhaps counter-intuitively, was as productive for commercialization as it was for science, helping to develop products that built on the HGP’s sequences funneled daily into the public domain.

Yet the Celera challenge also elevated the Bermuda Principles, exaggerating the necessity of daily sharing in a manner quite inconsistent with their history. The Principles, as a powerful foil to Craig Venter’s efforts, began as a radical proposal for data sharing, but within 2 years grew into a rallying cry for public science akin to a defense of the public HGP itself. Robert Waterston believes that the Bermuda agreement “clearly attained more significance in’98 with Celera’s entry … and a clearly competing model … I’m not sure it would have had the impact it did without Celera” (Waterston and Sulston interview 2011). Craig Venter himself quipped in 2012: “You still see things written that they had to dump the data every night because the evil guy … was patenting the whole genome” (Venter interview 2012).

This constructed contrast between the two projects, however, and the associated public attention, obscured (and continues to obscure) the detailed history of the pragmatic issues, in particular data quality and project coordination, for which the Bermuda Principles were initially employed. Daily data release had always faced opposition, for instance from Mark Adams, Craig Venter (who had hated the notion well before Celera), and Maynard Olson, each of whom had harbored serious concerns about data quality.^72^ Moreover, daily sharing, as modeled after the practice pioneered in *C. elegans* mapping and sequencing, had never been the only system available to accomplish these pragmatic goals. The yeast and mouse genome projects had provided other possible examples of pre-publication sharing, and even if Craig Venter kept his promise of quarterly data release, daily release would not have been necessary to beat him.^73^ The apparent urgency of daily data sharing and, indeed, the appearance that the HGP’s sequencers had always adhered to it, was largely an artefact of the rhetorical purposes for which it was employed, mostly in^72^ Interestingly, a second issue with data sharing had also plagued both TIGR’s relation to Human Genome Sciences and HGS’s agreement with SmithKline Beecham. Venter’s TIGR, a nonprofit laboratory, fought mightily and repeatedly with its for-profit unit, HGS, which did not adhere to TIGR’s preferred data-release policies. The company wanted to keep data private for at least a year, while Venter wanted TIGR’s ESTs shared at the time of publication. To make his case, Venter used the Merck Gene Index project “as a weapon,” arguing that since “similar sequences are out there … TIGR is free to go ahead and publish them” (Venter interview 2012). Venter, it appears, did not like either extreme. Ultimately, the issue led to the termination of his partnership with HGS, as Venter described in his post-hearing questions in 1998: on “June 20, 1997, The Institute for Genomic Research (TIGR) and Human Genome Science[s] (HGS) ended a collaborative arrangement that required TIGR to forego payments totalling [sp.] $38 million. The primary reason for my choosing to end this relationship and access to significant financial resources was a philosophical disagreement about the public release of DNA sequence data. The day after this relationship was terminated, TIGR made the largest deposit of DNA sequence data into the public domain in history” (*How Private Sector Developments Affect the Government Program*, 98). 1998 and afterwards. These purposes were the defense of the HGP under an imminent threat, alongside the creation of a genome commons before—as many in the HGP’s leadership feared—Celera locked up the human genome sequence in a restrictive database, effectively hijacking the basis for future discoveries.

The HGP-Celera race ended in a brokered tie, a “truce negotiated by Ari Patrinos” (Shreeve 2004, p. 360), a skeptic of the Bermuda Principles from the beginning. On June 26, 2000, a joint press conference was broadcast from the White House and simulcast with 10 Downing Street, as Craig Venter and Francis Collins flanked President Bill Clinton at the podium in the West Wing and UK Prime Minister Tony Blair joined to declare the “Completion of the First Survey of the Entire Human Genome” (Office of the White House Press Secretary 2000; also, Macilwain 2000). The rival draft sequences were published a day apart, Celera’s in *Science* and the HGP’s in *Nature* (Lander et al. 2001; Venter et al. 2001), with Eric Lander heading the imposing list of authors in what came to be known as the “International Human Genome Sequencing Consortium (IHGSC).”^74^ The HGP paper emphasized that, “sequence data have been made available without restriction and updated daily throughout the project” (Lander et al. 2001, p. 860). The terms of the public-private “tie,” in turn, were negotiated by the editors of the two journals, and stipulated that Celera could publish about its genome in *Science* while still restricting the underlying data to its proprietary website, so long as academic and nonprofit researchers had “free access” and were “allowed to patent at will any discoveries they made using Celera’s information without any commercial obligation to Celera” (Shreeve 2004, p. 361).^75^

Celera’s assembly also relied on the HGP’s sequences, however. The feverish press coverage that followed documented debates between the HGP’s leaders (especially Eric Lander) and Celera’s scientists, regarding the relative quality and completeness of the two rival genomes (for instance, Ghosh 2001; Shreeve 2004, Epilogue). The follow-on analyses explored just how critical the HGP’s sequences, released under the Bermuda Principles, proved to the final Celera product, appearing in tit-for-tat publications from 1998 through 2003 (Venter et al. 1998; Waterston et al. 2002; Myers et al. 2002; Adams et al. 2003). Celera never did release its data quarterly, and most of its database users required an expensive subscription to gain access to more than 1 Mb of its sequence (Williams 2013, pp. 10–12; Hilgartner 2017, p. 221). From a technical standpoint, moreover, it is not even clear what quarterly release would have meant for Celera’s data, given that its WGS strategy did not modularize to contig-by-contig sharing. Yet the company’s eventual patent portfolio—the greatest fear from the beginning—was modest. Venter received only 15 DNA patents attributable to human genome sequencing, mostly from his tenure at TIGR (Cook-Deegan and Heaney 2010, p. 404).

The success of the Bermuda Principles, in perpetuating and preserving the HGP, fueled their extension to additional contexts. In 2003, the year the IHGSC declared the HGP complete, the Wellcome Trust convened a meeting in Ft. Lauderdale, Florida (International Consortium Completes Human Genome Project 2003). This was an explicit continuation of the Bermuda tradition, but it was also prompted, thanks in part to heavy pressure from Eric Lander, by Celera’s appropriation of the HGP’s data alongside other recent controversies regarding the fair use of sequences in journals (*Sharing Data from Large-Scale Biological Research Projects*; Lander interview 2012; Hilgartner 2017, Chap. 6, esp. pp. 175–181). The NHGRI’s data-sharing policy from 2000, which had extended the Bermuda Principles to WGS data, included stipulations for HGP-related publications, declaring that while the generators of NIH-funded sequences reserved the rights to publish their initial assemblies and broader analyses, more directed investigations, including the identification of genes, were fair game for others to publish (NHGRI Policy for Release and Database Deposition of Sequence Data 2000). The fair use issue had infiltrated the Biology of Genomes Meeting at CSHL in 2001, where the participants had debated the strange question of when scientists could claim authorship rights for accomplishments that they had yet to complete (Hilgartner 2017, p. 178). The 2003 meeting in Ft. Lauderdale addressed a similar issue, but for the post-HGP era. The conference assessed, in Stephen Hilgartner’s words, how authors and journals should treat “UJAD data,” which was “unpublished in journals but available in databases” and, as a consequence, required different rules for governance and control (Hilgartner 2017, pp. 175–185, 229).

The echoes from Bermuda, by design, were loud. The published rationales of the meeting were “to discuss how, at this point in the development of the field of genomics, pre-publication data release can promote the best interests of science and … maximize the public benefit to be gained from research” (*Sharing Data from Large-Scale Biological Research Projects*, p. 2). The central outcome of the meeting was the formalization of the concept of “community resource projects,” to which the Bermuda Principles were officially extended and applied. These kinds of projects—similar to their exemplar, the Human Genome Project—were “specifically devised and implemented to create a set of data, reagents or other material whose primary utility will be as a resource for the broad scientific community” (Ibid.). Two such projects underway by 2003 were the Single Nucleotide Polymorphism (SNP) Consortium, focused on finding single-base pair variations in human genomes, and the International HapMap Consortium, devoted to finding other common human genomic variants (Holden 2002; Gibbs and The International HapMap Consortium 2003; Thorisson and Stein 2003; Thorisson 2005). A crucial difference between these community resource projects and the HGP, however, was that rapid pre-publication data release, rather than daily data sharing, was the explicit objective. The success of the Bermuda Principles did drive their extension to further projects, but this occurred in a flexible, rather than a literal, sense.

The 2003 Ft. Lauderdale report, *Sharing Data from Large-Scale Biological Research Projects: A System of Tripartite Responsibility*, defined three categories of stakeholders. It asked data *producers* to share data early and without restrictions on access, data *users* to responsibly and clearly acknowledge producers, and science *funders* to support the processes related to, and the infrastructures required for, rapid data sharing. Especially after the “completion” of the HGP declared in 2004 (International Human Genome Sequencing Consortium 2004), this ethos spread to scientific congresses in Amsterdam (2008) and Toronto (2009), where the Bermuda Principles, or at least rapid, pre-publication data sharing, were extended to additional kinds of genomic and molecular proteomic data (Rodriguez 2008; Rodriguez et al. 2009; Toronto International Data Release Workshop 2009). Several legal scholars have examined these developments, and more recent extensions of the NHGRI’s data-sharing policy, in broader law and policy frameworks (Contreras 2010, 2011, 2014, 2015, 2016, 2017; Arias et al. 2015; Lee 2017). As Hilgartner has also noted, however, the Ft. Lauderdale report’s language of “rights” and “etiquette” meant that in most cases, as with the Bermuda Principles during the HGP, rapid data sharing in community resource projects had no formal enforcement mechanisms, requiring community buy-in, or peer pressure, for implementation and maintenance (Hilgartner 2017, pp. 178–184, and ch. 8, esp. p. 229).

The core imperatives and challenges for genomic data sharing persist to the present, even if the finer details of the process have changed with time. Many analysts believe that we have entered a “postgenomic” era, wherein genomes are understood as responsive, networked feedback loops rather than as the determinist entities they represented for most of the twentieth century (Richardson and Stevens 2015). This paradigm shift notwithstanding, genomic data remains the most scientifically valuable in statistical aggregate, in the context of thousands or millions of sequences, mutations, markers, and structural and functional studies. Molecular biology in 2018 looks much different than in 1988, when the data to be shared were genetic and physical maps with mostly un-annotated sequences. Even so, rapid sharing is more pressing a concern than ever, including for the pragmatic issues of data quality, interpretation, and project coordination, though it grows unprecedentedly complex with ethical concerns such as fair use, medical privacy, and unfair genetic discrimination (Heeney et al. 2010; Lemke et al. 2011; Tenopir et al. 2011; Dove 2015; Majumder et al. 2016; Vayena and Gasser 2016; Zarate et al. 2016; Cook-Deegan et al. 2017; Deverka et al. 2017; Reardon 2017).

Defining a genome commons and understanding its relationships to patenting and commercialization is also a daunting challenge in personalized and precision medicine, just as it was when DNA sequencing first infiltrated molecular biology in the 1970s (Boyle 2010; Reardon et al. 2016; Cook-Deegan et al. 2017; Deverka et al. 2017; Parthasarathy 2017; Reardon 2017). The nature of data itself, and the pace of technological change, are in flux now more than ever before, but rapid data-sharing policies remain integral to the practices of genomics and the other life sciences, such as proteomics, which rely heavily on the widespread availability and community interpretation of vast quantities of data (Rodriguez 2008; Rodriguez et al. 2009; Toronto International Data Release Workshop 2009). The term “open science” is also a buzzword for scientists and policy analysts—it has been since at least the start of the HGP—though little consensus exists about the meaning of open science or the best ways to practice it (Grubb and Easterbrook 2011; *Science as an open enterprise*; Davies et al. 2013; Leonelli 2013b; Levin and Leonelli 2016). The label generally refers to the unrestricted sharing, after publication, of the datasets underlying journal articles, yet pre-publication sharing is also an integral part of many open science frameworks, and is an activity that can be multiply realized in implementation and justification.

The timing of data release, and of journal publication, are perennially disjoint issues. Some data categories (such as maps, sequences, and fragments of anonymous, in-progress reference genomes) are highly amenable to pre-publication sharing. But others, such as patient health records and identifiable genomic sequences, are—for good reason—subject to privacy and ethical constraints that rarely, if ever, allow them to be shared even at publication. The strategies and policies for effective data release in any project must therefore depend on numerous factors, including but not limited to: data types; technological feasibility and implementation; community buy-in; project organization and goals; funding structures; local and international policies and laws; benefits and drawbacks of commercialization; and risks and rewards for all of the stakeholders, including for research subjects and those who stand to benefit or be harmed by the investigations at hand (Knoppers et al. 2011; Knoppers 2014; Cook-Deegan et al. 2017). The chosen path to data sharing in a given project may sometimes involve traditional publication models; in other projects, as with the HGP, standard journal practices may be heavily revised, even bypassed. Human genomics carries heavier symbolism, commercial potential, and anticipated medical risks and rewards than most model organism research, such as with the nematode worm *C. elegans*. Nevertheless, the legacies of the nematode community’s goal-directed, infrastructural work—which harmonized with, but surpassed in their radical sharing terms, the data-sharing norms in other model organism groups—remain perceptible in today’s data-sharing challenges, even if the original practices from nematode met friction in their translation to human genomics. Attention to history, in all of its rich contingencies, is crucial for biologists and policymakers, as they look to the past for precedents, interpret the present, and plan for the future.

## Conclusions

The Bermuda Principles stemmed directly from *C. elegans* biology, so much so that one attendee at the 1996 Bermuda meeting remarked, “I feel slightly uncomfortable about the notion that we would hang the human genome on the worm! Although, of course, historically, for the groups concerned, it is very much the way it’s come about” (1996 Bermuda Meeting Transcript, Session II, “Sequence-Ready Maps”). Nevertheless, despite this remarkably strong connection, daily data release was far from inevitable. Historical actors all work within the finite resources available to them, and no moment in the past has offered infinite possibilities (Hilgartner 2017, Chap. 8, esp. pp. 230–231). Robert Waterston, John Sulston, James Watson, Francis Collins, Michael Morgan, and Eric Lander all moved with great agency, but within existing frameworks of moral economy and policy. At any point, the most powerful argument for daily data sharing, at least scientifically, was always in its successful implementation in preceding projects, whether in *C. elegans* mapping and sequencing, human mapping and sequencing, or the broader HGP. The Bermuda Principles, moreover, and especially their daily data-sharing mandate, were often—perhaps even *usually*—aspirational when it came to practice, and they endured largely as an archetype for how pre-publication sharing was variously realized during the HGP. The many iterations of the policy wording, from Sulston’s initial white board draft in 1996 to the National Research Council’s reproduction of the Principles in 2006, reinforced this point. The Bermuda Principles were also flexible in their justifications; the arguments in their favor, whether for pragmatic outcomes such as data quality and coordination or for those related to the commons, changed over time and in varying contexts, especially in the public defense of the project after the Celera challenge beginning in 1998.

The Bermuda Principles were not a sociotechnical imaginary; they never were, nor are, institutionalized enough for this label (Jasanoff 2003, 2004, 2005, 2011; Jasanoff and Kim 2009, 2015; Hilgartner 2017). They did, however, constitute a groundbreaking, controversial “regime” for sharing data—one of the several that characterized the HGP from 1988 to 2003 (Hilgartner 2017, Chap. 6)—because they mandated that the responsibilities, entitlements, and burdens for data sharing be redistributed, causing disruptions and reorganizations in the regular workflows of genome science. The Bermuda Principles were certainly what Hilgartner terms a “vanguard vision,” developed and endorsed by influential, and principally Anglo-American, elites and institutions, even as this vision proved imperfectly implemented in the HGP’s laboratories in practice (Hilgartner 2017, esp. Chap. 8). As David Cox recalled in 2011, during the 1996 Bermuda meeting, “Michael Morgan got up and clinked his glass and had everybody stand up and sing, *God Save the Queen*” (Cox interview 2011).

Yet three further forces, related to the hegemony of the American and British scientists in the HGP, also helped drive this process. The first was the steadfast advocacy of John Sulston and Robert Waterston, the main architects of the Bermuda Principles in 1996. The second was the support of powerful scientists and administrators in the Wellcome Trust and the US NIH, most notably Michael Morgan, James Watson, Francis Collins, and Eric Lander (as an NIH grantee). As the worm community coalesced and grew through the 1980s and 1990s, Sulston and Waterston convinced others, including Collins, of the advantages of daily data sharing, resulting in the NIH and the Wellcome Trust adopting the Bermuda Principles as HGP policies quickly relative to the project’s other major funders.^76^ These two institutions, which could have sequenced the entire human genome without collaborators, then drove the attempted and actual implementation of the Bermuda Principles in the HGP and later genome-based fields, helping overcome the resistance to, and the commercialization policies incompatible with, daily release, including at the DOE. But the desires of the German, Japanese, and French scientists and administrators to remain a part of the HGP also proved integral to the survival of the Bermuda Principles, helping to broker the “measured consensus” that eventually surrounded them and their adoption in the project. Daily data release may have become a formal grant condition at the NIH and the Wellcome Trust, but the scientists and administrators at other major HGP funding agencies, particularly outside the US, found ways to sidestep or revise their existing polices just to remain compliant. Biology collaborations are often transnational, especially in the digital age (Maienschein 1993; Parker et al. 2010; Stevens 2013, 2017). In this case, however, “consensus” was also channeled through the local, the institutional, and the national, and in particular through the policies of major science funding agencies in powerful industrialized states (Kleinman 1995; Jasanoff 2005). This apparent “consensus” was fostered even further by the fact that, in reality, daily data sharing was hardly ever realized literally, especially before 1998.

The historiography of the HGP is maturing, complemented by new archives and digital resources (Aircadi and García-Sancho 2016; Shaw 2016). A notable example is the NHGRI’s History of Genomics Program, run by the historian Christopher Donohue, which features the history of genomics at NHGRI, and especially the ELSI program, through papers, oral histories, and more (NHGRI History of Genomics Program 2018).^77^ This next generation of scholarship is adding to perspectives already present in the many popular accounts (Sulston and Ferry 2002; Shreeve 2004; Venter 2007; Angrist 2010; Davies 2001, 2010; McElheny 2010; Wade 2014; Watson et al. 2017) and the large sociological literature (Hilgartner and Brandt-Rauf 1994; Hilgartner 1995b, 1997, 1998, 2004a, b, 2012a, b, 2013; Balmer 1996a, b, 1998; Jasanoff 2003, 2004, 2005), sharpening as well an historiography that has tended to focus on ELSI, big science, institutional histories, and politics (Heilbron and Kevles 1988; Kevles and Hood 1992; Cook-Deegan 1994; Kevles 1997, 2012; de Chadarevian 2002; Reardon 2005; Rajan 2006; Bartlett 2008; Aronova et al. 2010). This most recent work, particularly in the last half decade, has debunked the HGP as a teleological progress narrative, detailed its technological foundations, extended the previous analyses of the ethical, legal, and social implications (ELSI) of DNA sequencing and related technologies, and explored the evidence for our transition into a postgenomic era (November 2012; Stevens 2013, 2015a, b, c, 2017; García-Sancho 2015; Richardson and Stevens 2015; Leonelli 2016; Parthasarathy 2017; Reardon 2017). Further analyses are needed, however, particularly regarding the highly technical histories of assembling map-based and WGS genomes using programs such as *phred*, *phrap*, and *consed*; the evolving technologies for and related to sequencing, such as fluorescent capillary sequencing, advanced DNA polymerases and related enzymologies, BAC cloning, and “next-generation” (also known as post-Sanger) sequencing (Mardis 2008; Shendure and Ji 2008; Curnutte et al. 2014, 2016); the model organism and other kinds of biomolecular databases (as in, Leonelli and Ankeny 2012); human variation research; functional and regulatory studies of the genome; and integrative and translational genomic medicine. For the most part, in the present story, we have referenced the fundamental technological milestones throughout the HGP, but have far from fully developed their histories.

Our history links the HGP directly to nematode biology, and less directly to the practices and leadership of other influential model organism communities. Most of the rich historiography of model organisms has explored how practices, materiality, politics, theories, moral economies, and cultures, including norms of rapid and pre-publication data sharing, have evolved in relatively coherent and discrete communities (see, for instance, Kohler 1994; Ankeny 1997, 2001; Creager 2001; de Chadarevian 2004; Rader 2004; Leonelli 2007). More philosophical and ethnographic scholarship, in turn, has examined how such model organism-based knowledge has scaled to the human and broader medical sciences, creating generalizable and actionable conclusions from specific experimental circumstances (de Chadarevian 1998; Ankeny 2000; Creager et al. 2007; Nelson 2017). This large body of work, however, has spent less time exploring the social and political dynamics by which model organism communities have intermixed and interacted, especially with human biology. We have detailed an example of a deliberate and direct transfer of practices from *C. elegans* molecular biology to human genomics. In so doing, we have attempted to lay bare the political valence of the human genome, especially regarding the patenting of genetic materials and the attendant commercialization structures historically associated with molecular biology (see also, Parthasarathy 2017). Daily data sharing was, at first, very controversial in human genomics: not just because the HGP’s early data-sharing policies were ambiguous, but also because the numerous imagined paths to sharing and commercialization often came into conflict. In the worm, patents mattered little; in humans, they seemed crucial. What also emerges from this history, however, is that the many justifications for the Bermuda Principles, especially for daily sharing, were hardly neatly demarcated.

In what sense were the Bermuda Principles truly “principled,” akin to what Aristides Patrinos described as “religious” (Patrinos interview 2011) and Elbert Branscomb likened to “puritanical” (Branscomb interview 2011), or motivated by “conversion of a technical issue into a moral one” (Olson to Waterston 1996)? In reality, the problems addressed by the Principles fell along a spectrum, in the “topsy-turvy” world where “pragmatic” goals, such as the completion of a high-quality human reference genome in the public domain, also qualified as “principled” outcomes, assuming that the product in question did, as the HGP’s advocates hoped, translate into public goods such as downstream research and commercialization (Cook-Deegan and Heaney 2010, p. 402).^78^ The preferred paths to these outcomes, moreover, were always contested and contingent, with pragmatic and principled arguments by the varied stakeholders, whether over data release and quality, patenting and the genome commons, or other issues, vying for supremacy in the administration of the project. For Sulston and Waterston, daily data release, or at least working towards it, fulfilled what we have termed the “pragmatic” goals of quality control and project coordination, building on their vast experience with sharing in the nematode worm community. But the Bermuda Principles’ supporters also argued that, especially for the human genome, this strategy was the *right* way of doing publicly funded science, because it prevented overly broad patents on genes, promoted follow-on research and commercialization, and ensured broad access to data that might tell us something fundamental about humanity. *Daily* sharing, however, was only one strategy for rapid sharing; its consequences for the commons and for patenting were just one orientation, a particular policy configuration, for how public science might be translated into principled social outcomes.

Were the Bermuda Principles optimal? We do not know. It was precisely because the Principles absorbed and embodied so many scientific and principled meanings over time, which together constituted a multifaceted and coherent stance towards public science and the commons, that they engendered such great conflict, and yet, especially during the Venter race in 1998 and afterward, were reinforced. Moreover, it was through their aspirational and paradigmatic nature that the Bermuda Principles could repeatedly be so imperfectly implemented in policy and in practice, yet still remain as a spiritual rallying cry for the HGP, one that eventually stood in as a justification for the project and proved consistent with its public works mandate from the very beginning. In this sense, the Bermuda Principles functioned like a boundary object (Star and Griesemer 1989; Bowker and Star 1999; for an analysis of the UK HGMP as a boundary object, see Balmer 1996b), or perhaps as a “boundary policy.” In 2012, Maynard Olson quipped of the Principles: “there was this platonic world of ideal forms of 1-kb contigs and smoothly flowing data. Then in the lab, we had technicians getting sick. We had computers breaking down” (Olson interview 2012). The Bermuda Principles were certainly an archetype, especially for the less technologically privileged HGP centers. Inside and outside the HGP, many genomics research groups were simply not equipped with the technical capacities that would have been necessary to comply with the Bermuda Principles literally, let alone to carry out some of the lines of the follow-up research the Principles were supposed to promote.

There is also no doubt that the DOE, German, French, and Japanese scientists and administrators could have been treated more considerately, nor that the outcome was heavily biased towards the preferences and philosophies of Anglo-American scientists and institutions. Even Aristides Patrinos felt that the Bermuda Principles sounded “like a declaration of war, an attack against individuals or groups that for whatever reason could not endorse something like this because their governments had different notions” (Patrinos interview 2011). The hegemony of the NIH and the Wellcome Trust in the HGP does raise concerns about small, elite groups of funders controlling global science policy, especially in instances where the outcomes might not be so benign. How the benefits and risks of genomics research and development could, moving forward, be distributed more justly, given technological, educational, health, economic, and other disparities, remains a set of open and essential questions in the post-HGP world (Knoppers et al. 2011; Knoppers 2014; Reardon et al. 2016; Cook-Deegan et al. 2017; Reardon 2017). These interdisciplinary issues, indeed, should continue to draw sustained and critical attention from a range of viewpoints, including from historians, philosophers, social scientists, ethicists, legal and policy analysts, and scientists.^79^

At least in terms of the goals set in relation to them, however, the Bermuda Principles were a wild success. In the face of the private threat from Celera Genomics, the HGP remained on-task and efficient. The project was completed ahead of schedule, achieving widely accepted quality standards by the time of its publications and making primary DNA sequences and much else available in the public domain. The policy implications were profound, largely because pre-publication sharing proved useful for industry as well as for science (Williams 2013; Parthasarathy 2017). In 1996, one Bermuda attendee noted presciently: “If we sincerely agree that this is our aim, then, as a group, we shall change history a little bit” (1996 Bermuda Meeting Transcript, Session V, “Panel/Open discussion”). Francis Collins believes “this was a signature moment,” a “change in the ethics of how you do this kind of project” (Collins interview 2012). After the HGP, John Sulston himself left laboratory science largely behind, becoming a scientific statesman committed to promoting “freely accessible basic information” in the interpretation of the human genome (Sulston and the International Human Genome Sequencing Consortium 2001, p. 31). As he wrote in an article entitled “Society and the Human Genome” in 2001: “Many more large-scale projects, involving proteomics, structural biology, neurobiology, and so on will be needed…. The more communication we can have the better and faster it will go” (Ibid.). Ten years later, in 2011, Sulston reflected that, “I think that these principles extend to the way we need to run the world” (Waterston and Sulston interview 2011). The Bermuda Principles have certainly extended far beyond Bermuda.

## Figures and Tables

**Table 1:** Summary of Principles Agreed Upon at the First International Strategy Meeting on Human Genome Sequencing (Bermuda, 25–28 February 1996) as reported by HUGO

The following principles were endorsed by all participants. These included officers from, and scientists supported by, the Wellcome Trust, the U.K. Medical Research Council, the NIH NCHGR (National Center for Human Genome Research), the DOE (U.S. Department of Energy), the German Human Genome Programme, the European Commission, HUGO (Human Genome Organisation), and the Human Genome Project of Japan. It was noted that some centres may find it difficult to implement these principles because of legal constraints and it was, therefore, important that funding agencies were urged to foster these policies
*Primary genomic sequence should be in the public domain*
It was agreed that all human genomic sequence information, generated by centres funded for large- scale human sequencing, should be freely available and in the public domain in order to encourage research and development and to maximise its benefit to society
*Primary genomic sequence should be rapidly released*
Sequence assemblies should be released as soon as possible; in some centres, assemblies of greater than 1 Kb would be released automatically on a daily basis
Finished annotated sequence should be submitted immediately to the public databases
It was agreed that these principles should apply for all human genomic sequence generated by large- scale sequencing centres, funded for the public good, in order to prevent such centres establishing a privileged position in the exploitation and control of human sequence information
*Coordination*
In order to promote coordination of activities, it was agreed that large-scale sequencing centres should inform HUGO of their intention to sequence particular regions of the genome. HUGO would present this information on their World Wide Web page and direct users to the Web pages of individual centres for more detailed information regarding the current status of sequencing in specific regions. This mechanism should enable centres to declare their intentions in a general framework while also allowing more detailed interrogation at the local level

## Footnotes

1 For much of the twentieth century, genes were understood as the “expressed” parts of genomes, serving as the coding templates for proteins or RNA products through the genetic code. Genetics was the study of inheritance, molecular genetics the study of genes in relative isolation, and genomics the study of genes in concert and especially with computational methods. The “postgenomic” genome, a descriptor generally applied to genomics research after the HGP, is understood in contrast as a dynamic system of networked feedback loops (Judson 1992; Cook-Deegan 1994, Chap. 1; Kay 2000; Powell and Dupré 2009; Baetu 2012; Richardson and Stevens 2015; Stevens 2015b; Hilgartner 2017, esp. Chap. 2).

2 While the HGP was indeed declared “complete” between 2003 and 2004, hundreds of gaps, mostly repetitive regions, technically still remain in human genome. These gaps exist because even with the sequencing technologies available today, we cannot read them (Begley 2017).

3 Articles began rivaling books as the standard currency of natural philosophy in the late seventeenth century. By the mid-twentieth century, sharing research results at the time of publication, and not before, was a widely accepted scientific norm (de Solla Price 1963; Merton 1973; Hagstrom 1982; Gilbert et al. 1984; Latour 1987; Bazerman 1988; Hilgartner and Brandt-Rauf 1994; Hilgartner 1995a; Nummedal and Findlen 2000; Biagioli 2012; Ankeny and Leonelli 2015, p. 138). In a brief account of how “science studies scholars of the 1960s and 1970s first glimpsed the extent and significance of scientific collaboration through networks of co-authorship derived from the by-lines of articles,” Edward Hackett observed in 2013 how “publication is the existential act of science, and publications are the artifact that remains as evidence that the existential research act was consummated” (p. xvii).

4 While they have collected various names over the years, including the “Bermuda Resolution,” “Bermuda Agreement,” “Bermuda Rules,” “Bermuda Protocol,” and “Bermuda Accord,” we adopt “Bermuda Principles” predominantly here, as the most commonly-used term in the contemporaneous documentation.

5 As Stephen Hilgartner has recently noted, completing the genome “ahead of schedule and under budget” was of great political importance to the project (Hilgartner 2017, esp. p. 221). The NIH and the Wellcome Trust, the two predominant funders, ultimately provided nearly 90% of the funds for the HGP human genome, producing nearly the same percentage of the sequence (Collins to sequencers and data 2000).

6 Because most mapping and sequencing data can, and could for at least the latter part of the period under study, be stored and communicated digitally, we use the term “data” consistently with recent scholarly depictions of biology as a “data-driven” or “data-centric” science (Strasser 2012; Stevens 2013 2015a, b, c, 2017; Ankeny and Leonelli 2015; Leonelli 2016; for a similar presentation of “biomedicine as an information science,” see Lenoir 1999). This choice also aligns with our historical sources. Yet, additionally, we acknowledge the historicity and malleability of data as a category (Hilgartner and Brandt-Rauf 1994), as demonstrated in the HGP’s changing data-sharing policies for mapping and DNA sequences of different lengths and obtained through various methods and organisms (see below). Finally, our usage of the term “data” is not to deny the significance of other kinds of biological data, such as in taxonomic research, from before the influx of digital computing into the life sciences in the 1960s and 1970s (Lenoir 1999; November 2012). As Bruno Strasser has observed: “If there is any novelty to be found in today’s data-driven science, perhaps it might derive from the amount and nature of the data”; and given “that there is no early modern equivalent to the petabytes of digital data stored in computerized databases…. [I]t is more useful to compare the *dynamics* of data accumulation within research fields which [have] relied on analog and digital data, respectively” (2012, p. 86, italics in original). One of our goals in the present piece has indeed been to analyze these kinds of dynamics, and in so doing to document the reciprocal constructions of data accumulation, data sharing, and science policy as molecular biology transitioned from its classical era to genomics.

7 In addition to the published literature, we draw predominantly on our own semi-structured interviews with participants in the HGP and also a large collection of unpublished sources obtained through Freedom of Information Act (FOIA) requests to the NIH, from private hands, and from various archives. We cite in text all of our interviews and archival sources using parenthetical citations, with the full citations (i.e., interview dates and transcripts, URLs where available, and full archival finding aid information) for these items available in the References section. The current article was submitted and accepted for publication before Stephen Hilgartner’s recent sociological study of the HGP was published (2017), but we build upon and respond to his analyses here. Our conclusions are not only mutually compatible, they are mutually reinforcing, and this paper is far better than it might have been thanks to his contributions on this topic.

8 The co-production framework, as Hilgartner points out, extends the conclusions of Steven Shapin and Simon Schaffer in *Leviathan and the Air Pump* (1985), “using comparative analysis of law and politics” and proceeding “from three main assumptions: (1) that ways of knowing the world and ways of ordering social relations are deeply interconnected, forming a single mode of ordering knowledge and society; (2) that established orders change through processes in which knowledge and social relations are mutually adjusted; and (3) that analyzing these processes requires being symmetrically attentive to changes in knowledge and governance” (Hilgartner 2017, p. 6).

9 As the sociologist Karen Knorr Cetina argued, until the HGP, or at least until the rise of projects tackling the sequences of whole or large parts of genomes, molecular biology operated mostly as science driven by smaller groups at individual laboratories (Knorr Cetina 1998, esp. Chaps. 2, 4, 6, and 9). Hilgartner extends her argument from Germany to the US (Hilgartner 2017, esp. Chap. 2, n. 62).

10 Mapping identifies the genomic addresses of markers that can orient sequencing. Various sources have provided descriptions of the data produced in both mapping and sequencing, as well as of the differences between genetic, physical, and genomic maps and how they can be used to form more complete genomes (*Mapping and Sequencing the Human Genome*, Chaps. 1–2; *Mapping Our Genes*, Chaps. 1–2; *Understanding Our Genetic Inheritance*, Introduction, Program Goals, and Scientific Goals; Hilgartner 2017, Chap. 2). See also, below.

11 The “Community of *C. elegans* Researchers,” for instance, is listed as an editor of the influential book, *The Nematode Caenorhabditis elegans* (Wood and the community of *C. elegans* researchers 1988).

12 Hilgartner focuses his analyses on resistance to the Bermuda Principles based on publication norms and project coordination, including concerns that article delays brought about by daily data release (combined with the long co-author lists so characteristic of mapping publications) might hinder junior investigators’ careers. He also notes that even among the HGP’s leadership, “the ‘consensus’ underlying the agreement” to the Bermuda Principles was thin, and that some actors, especially in the smaller American laboratories, felt pressured into them (2017, esp. p. 173 and Chap. 6, n. 69). Hilgartner does not, however, address why *daily* sharing in particular was adopted in lieu of other data-sharing options.

13 Various social scientists and historians have written extensively on moral economies in science (e.g., Merton 1973; Kohler 1994; Daston 1995; McCray 2000; Strasser 2011).

14 Exploring such dichotomies has been a theme in the STS co-production framework. Hilgartner recently described “the official constitution of a state, which we confuse with its actual mode of governance at our peril” (Hilgartner 2017, p. 13).

15 Gene patents stoked controversies and debates in the 1980s and 1990s, as demonstrated by government reports and other policy analyses (*Commercial Biotechnology*; *New Developments in Biotechnology*; Cook-Deegan 1994; Burris et al. 1998; Parthasarathy 2007, 2017). Hilgartner also provides a useful summary (2017, Chap. 5).

16 De Chadarevian has argued that while in the 1970s Brenner sought a programmatic connection between genes and behavior, inspired by the French Pasteurian operon model (Gaudillière 1993; Judson 1996, pt 2), by 1984 he had grown skeptical of this view and sought instead an understanding of how genes affect biochemical cellular environments and influence behavior (de Chadarevian 1998, esp. pp. 96–97).

17 Sanger had already won a Nobel Prize in 1958 for his amino acid sequence of the protein, insulin. In 1980, he received the Prize again for his DNA sequencing method, sharing it with Walter Gilbert of Harvard, who had developed a competing method with Alan Maxam in 1977 (Maxam and Gilbert 1977). Initially, Maxam and Gilbert sequencing was more resilient, but Sanger’s was more readily automated (García-Sancho 2015, pt 3). Paul Berg of Stanford also shared the Prize in 1980 for methodologies related to recombinant DNA (rDNA).

18 *ACeDB* was developed in Sulston’s laboratory to facilitate *C. elegans* sequencing (Stein et al. 2001; Bostanci 2004, pp. 164–165; de Chadarevian 2004, pp. 106–107). Throughout the 1970s and into the 1980s, however, the most sophisticated application of computing to molecular biology was probably “MOLGEN—Applications of Symbolic Computation and Artificial Intelligence to Molecular Biology,” a project and network run by Joshua Lederberg and Edward Feigenbaum at Stanford that allowed access to software in order to “manipulate protein and nucleotide sequences … download sequences from databases and perform searches, analyze codon frequency, calculate base composition, align two sequences, and perform a range of other manipulations” (Stevens 2015a, p. 851; also, Roland and Shiman 2002, pp. 196–197).

19 Simon Pickvance, a graduate student in Brenner’s laboratory, was the first to use Nomarski optics to follow cell divisions in the embryo (Ankeny 1997, pp. 117–119). However, Pickvance ultimately left this problem, and molecular biology altogether, to follow a career in activism related to occupational health services (Pickvance et al. 2012).

20 Among biologists, the acronym “rDNA” more commonly refers to “ribosomal DNA,” or DNA located within the protein-making machinery of cells (Waterston to KMJ 2017). Because the term usually refers to “recombinant DNA” in the historiography, however, we adopt this latter usage here.

21 Contig maps, for instance, had gaps, because clones could be missing or overlaps could go undetected; and reproducing these maps required direct access to specific clone libraries, including the phage and host cell lines in which the target DNA had been replicated. Genetic maps, in turn, were generally low resolution, and markers were not distributed uniformly. Yet contig maps could be oriented and aligned if they contained genes on the genetic map; and “hybrid” maps could be undergirded by larger clone collections containing the sequences of missing fragments, allowing for gap closure. While the complete sequence of a genome and a genomic map are different things, one could conceive of the sequence as a map of the highest possible resolution.

22 Ultimately, both YACs and bacterial cosmids proved less stable and more prone to DNA rearrangements for the human genome than did bacterial artificial chromosomes (BACs), which after around 1999 were used for human sequencing (Marshall 1995, p. 784; Lander et al. 2001, p. 863; Sulston and the International Human Genome Sequencing Consortium 2001, p. 28; Weissenbach interview 2012).

23 Though Watson led the NCHGR only through late 1992, his opinion always carried weight. Following Watson’s departure, Michael Gottesman served briefly as acting NCHGR director, and Francis Collins took over operation of the NIH genome project in 1993 (Thompson 1993; Cook-Deegan 1994, pp. 339–341).

24 Three-quarters of Waterston’s NIH funds went to Washington University in St. Louis, while the other quarter went to Sulston’s group (Waterston to authors Jan 2017).

25 The WCS provided access to “gene descriptions, genetic maps, physical maps, DNA sequence, published literature, and unpublished and informal information,” making it possible as well “to search journal articles and newsletters and to navigate through genes, clones, physical maps, and anatomical and developmental databases” (Stevens 2015c, p. 479). The WCS was first released in 1991 and refined in 1993 (Pool 1993; Star and Ruhleder 1996).

26 The GDB “was accessible via the Internet, dial-up connections, file-transfer protocol, and the Wide-Area Information Service (WAIS)” (Stevens 2015c, p. 476).

27 Phil Green focused our attention on Hillier’s early work on *phred*, emphasizing that both *phred* and *phrap* were developed in the context of the *C. elegans* genome project, with the names of the programs inspired by other programs written by MRC worm collaborators (Green to KMJ 2018). Hillier did not mention working on *phred* in her interview with us (Hillier interview 2012).

28 The most comprehensive history of these early politics is Cook-Deegan (1994), upon which we draw, supplemented by other sources.

29 The Atomic Energy Commission was split into research and regulatory arms, the Energy Research and Development Administration (ERDA) and the Nuclear Regulatory Commission, until Congress created the DOE, which incorporated the ERDA research units and fulfilled a promise of President Jimmy Carter.

30 The idea of copyright for DNA sequences was floated in 1982, though trenchant critiques also appeared in that year (Kayton 1982; Kiley 1982, respectively). The concept was largely dismissed in two OTA reports, one in 1984 and the other in 1989 (*Commercial Biotechnology*, Chap. 16; *New Developments in Biotechnology*, Chap. 3, esp. pp. 33–34), though it did find its way into one of the HGP’s founding reports in 1988 (*Mapping and Sequencing the Human Genome*, 99). Today, at least for DNA sequences existing in nature without human intervention, the idea of copyright protection is mostly discredited. It was still a matter of some debate, however, in the early 1980s.

31 IntelliGenetics had already licensed the MOLGEN software from Stanford (Stevens 2015a, pp. 853–859, esp. p. 854). BIONET was based on this software, and by 1988 the network connected approximately 900 laboratories and nearly 3000 researchers around the world.

32 GenBank and the PDB did have divergent policies for data curation post-submission, however (Hilgartner 2017, Chap. 6, n. 50).

33 The NRC is a private, nonprofit research organization, founded in 1916 to advise the US government on scientific matters during the First World War (Kevles 1995, Chap. 8, esp. p. 112). The molecular biologist Bruce Alberts chaired the NRC study about the feasibility of mapping and sequencing large genomes (Cook-Deegan 1994, pp. 127–134).

34 Similar sharing patterns existed among *Drosophila* geneticists in the early twentieth century (Kohler 1994), but direct lines of continuity between these researchers and the Bermuda Principles are difficult to document.

35 Indeed, the yeast project developed its own “explicit set of rules,” which “designated some data as confidential and specified who had the authority to publish what, when, and under whose authorship” (Hilgartner 1998, p. 212, 2017, pp. 171–172). Participation incentives thus included privileged access to data, which could lead to publications or financial gains.

36 The sociologist Katherine McCain characterized genetics as “a discipline with a long history of openness in communication and expectations … that research-related information will be made available upon request” (1991, p. 495). By research-related information, McCain meant “stocks and strains, recombinant genomic libraries and clones, enzymes and vectors (physical products), computer software (research instruments), and techniques for producing or working with the above (research techniques)” (p. 500). Products also included datasets. Instruments, moreover, could include stock centers and repositories for model organisms, mutants, cell lines, phages, or plasmids; and techniques could include constructing recombinant clone libraries through published or unpublished manuals, protocols, and other media (pp. 500–506). But the sharing to which she referred applied mostly to the information behind publications, and to biologists from model organism communities: “Those working on basic molecular mechanisms,” she noted, “may behave more like ‘fly people’ than those working on medical applications” (p. 511). The implication, of course, was that individuals working on medically-oriented topics were more likely to behave secretively.

37 STSs are “operationally unique in the human genome (that is, [they] can be specifically detected via PCR [polymerase chain reaction] in the presence of all other genomic sequences)” (Olson et al. 1989, p. 1434). Moreover, “the overwhelming advantage of STSs over mapping landmarks defined in other ways is that the means of testing for the presence of a particular STS can be completely described as information in a database. No access to the biological materials that led to the definition or mapping of an STS is required by a scientist wishing to assay a DNA sample for its presence” (Ibid.). The polymerase chain reaction, developed in 1983, is a cornerstone technique in molecular biology that takes advantage of the complementary base-pairing properties of DNA to amplify tiny concentrations of target sequences, by the thousands or millions, both cheaply and accurately (Rabinow 1996).

38 The ELSI program spawned a field of its own, of which all three authors are beneficiaries (The Ethical, Legal, and Social Implications (ELSI) Research Program 2018).

39 As several scholars have noted, large-scale biological research, particularly in genetics and genomics, has often been justified with language of promises (Pauly 2000; Jasanoff 2005; Fortun 2008; Hilgartner 2017).

40 In the OTA report, James Watson even indicated that: “If they wished, either Western Europe or Japan could by themselves take on this project and it must be assumed that they will initiate their own efforts…. So it is a perfect project for us to pool our talents, as opposed to increasing still further the competitive tensions between the major nations of the world” (152, Box 7-D).

41 Lehrach’s strategy was called “high-density filter hybridization,” wherein radioactive DNA probes were washed over filters containing thousands of genomic samples. Based on complementary base pairing, these probes could be used to find clones containing particular sequences. The “reference library” of this information was based at Lehrach’s laboratory, which also coordinated the selective sharing of data and materials from Lehrach’s projects (Hilgartner 2004b).

42 Jean Dausset founded the CEPH with anonymous sponsorship in 1983 (*Mapping Our Genes*, p. 146). In 1980, he shared the Nobel Prize in Physiology or Medicine for his pioneering discoveries regarding the Human Leukocyte Antigen (HLA) system, a gene complex integral for immune system function.

43 RIKEN had been founded during the Meiji era, destroyed in WWII, and re-established in 1958 (Cook-Deegan 1994, p. 215).

44 *Mendelian Inheritance in Man (MIM)* began in 1966 and documented correlations between mutations (genotypes) and human diseases (phenotypes) (McKusick 1966; Lindee 2005; Comfort 2012). *MIM* was a major source for Margaret Dayhoff’s *Atlas of Protein Sequence and Structure* (Dayhoff et al. 1965; Strasser 2009; Ankeny and Leonelli 2015, p. 130). In 1987, this resource became available as *Online Mendelian Inheritance in Man (OMIM)*, through the Welch Medical Library at Johns Hopkins (Hamosh et al. 2005; McKusick 2007).

45 The available 1996 participants lists include those who were invited to the meeting, in both a draft (1996 Wellcome Trust Bermuda Provisional Participants List) and more finalized form (1996 Bermuda Programme and Participants List). We have been unable, however, to determine everyone who actually attended. The same caveat applies to the 1997 and 1998 participants lists (1997 Bermuda Revised Delegates List; 1998 Bermuda Programme and Provisional Delegates List). Mark Guyer published a list of attendees from the 1998 meeting, and there are some discrepancies between that article in *Genome Research* and the 1998 provisional delegates list (Guyer 1998).

46 In 1997, the Bermuda meeting report affirmed that there were indeed “four main groups with different sequencing targets,” but counted Hattori and Sakaki as the leaders of separate groups (1997 Bermuda Meeting Report). In 2012, Hattori said that he and Sakaki actually led the same group, and named the fourth as those at the Japanese Foundation for Cancer Research (Hattori to KMJ 2012).

47 The Whitehead Institute had also instituted a system for sharing materials. Thanks to an arrangement with a company in Alabama, researchers outside of MIT could obtain copies of the mouse mapping data alongside the genetic markers and associated PCR primers (which helped to amplify the markers) essentially at cost (Lander interview 2012).

48 Under the advice of Aristides Patrinos, the JGI consolidated the DOE’s highly technical genome efforts at the Lawrence Berkeley, Lawrence Livermore, and Los Alamos National Laboratories in 1997 (Branscomb interview 2011). Today, operated by the University of California and the DOE, the JGI represents the LBNL, LLNL, and the HudsonAlpha Institute for Biotechnology (About Us 2018).

49 While many experts now accept the utility of a gene-focused approach to sequencing, an emphasis on EST sequencing, rather than on whole genomes, had been discussed but rejected in the early HGP planning meetings (Cook-Deegan 1994, Chap. 19, esp. pp. 307–325; Sulston and the International Human Genome Sequencing Consortium 2001, p. 28; Branscomb interview 2011; Hilgartner 2017, Chap. 5).

50 Myriad was receiving worldwide negative press coverage (Baldwin and Cook-Deegan 2013). Amidst much litigation, sequence patents were granted to Myriad in 1997 and 1998, with further patents on mutations, antibodies, and detection methods granted between 1998 and 2000. Opposition was lodged in France, Belgium, and the Netherlands through the European Patent Office. Both Myriad and Mike Stratton, and their co-inventors, with the UK Cancer Research Campaign received *BRCA2* patents. In a landmark ruling in the summer of 2013, the US Supreme Court invalidated Myriad’s claims to DNA molecules corresponding to naturally occurring sequences (Association for Molecular Pathology v. Myriad Genetics, Inc.). Myriad subsequently sued seven competitors based on other patent claims, but the Court of Appeals for the Federal Circuit indicated that these claims were also invalid (Univ. of Utah Research Foundation et al. v. Ambry Genetics Corp.) and the lawsuits were later dismissed with prejudice or covenants not to sue (February 2015). This effectively ended litigation in the US; in 2015, the High Court of Australia similarly invalidated a claim to *BRCA*-sequence molecules (D’Arcy v. Myriad Genetics, Inc.).

51 Patent criteria in the US, which are similar worldwide, stipulate that inventions must be novel, non-obvious, and useful (35 USC. §§ 100–103). Though the Bermuda Principles created patent-defeating prior art, there were (and are) intricate debates about gene patents and the obviousness requirement, which fall beyond the scope of this article (Demaine and Fellmeth 2002).

52 In October 1999, Neal Lane, science advisor to President Clinton, wrote to the President that while the USPTO had “granted at least one patent on a partial gene sequence,” it was “rethinking its position” (Lane 1999).

53 Hallam Stevens has suggested that the Bermuda Principles explicitly benefitted larger laboratories (Stevens 2015c, pp. 492–493). Hilgartner, on the other hand, supports our view, that the Bermuda Principles were designed, at least in part, to democratize the access to and downstream utilization of the HGP’s sequences (Hilgartner 2004a, p. 125).

54 Branscomb obliged, as he was supposed to try to fill such gaps anyways (Branscomb interview 2011). Maniatis repaid him by giving a seminar in Branscomb’s laboratory.

55 For highlighting the inconsistencies between the original, 1996 HUGO statement and the 2006 NRC report, we are indebted to Jorge Contreras through both publication (Contreras 2011, p. 64, n. 6) and personal communication (Contreras to authors 2017).

56 Généthon raised (and continues to raise) its money by an annual Téléthon through the French Muscular Dystrophy Association (Association Française contre les Myopathies, AFM). By March 1995, the laboratory had reported 124 gene localizations and 15 gene identifications via its website and press releases (Kaufmann 2004, p. 147 and Figure 7.9).

57 In the early years of the HGP, the CEPH had acted as “an informal coordinator for approximately 40 investigators in Europe, North America, and Africa who use CEPH materials in exchange for reporting their data” (*Mapping Our Genes*, 145). In 1994, a controversy erupted when the CEPH intended to share French DNA samples with an American company, Millennium Pharmaceuticals, co-founded by Daniel Cohen (who had helped to found the CEPH with Jean Dausset; see *Mapping Our Genes*, 144–146, esp. Box 7-B) and Eric Lander of MIT (Rabinow 1999, pp. 1–2 and Chap. 15, esp. p. 117). In late 1993, the CEPH had beaten American teams in assembling the first physical map of the human genome. The Millennium deal, which was intended as a collaboration for the localization of genes for non-insulin-dependent diabetes, fell through, but it showcased how the connections between molecular biology and French industry, like elsewhere in the developed world, ran deep (Rabinow 1999).

58 The phrase “or better” was added, by hand, after the printing of the summary document, presumably (based on the provenance of the document) by Francis Collins (1997 Meeting Report Summary).

59 There was, in contrast, a DEC filed for the NIH’s Full-Length cDNA Initiative (NIH Initiative to Obtain Clones and the Full-Length Sequence for cDNAs 1999). The DEC, filed on November 16, 1999, read: “*The success of this initiative depends on the timely availability of all the clones and sequences generated by this initiative to ensure a publicly accessible gold standard set of reagents for biomedical research. The cDNA libraries, clones, and sequences generated as part of the NIH Full-Length cDNA Initiative will most effectively contribute to a resource for the research community if they are made publicly available without restriction and in a timely manner. The sharing of materials and data in a timely manner has been an essential element in the rapid progress that has been made in biomedical research*” (italics in original; Data Release and Access Principles and Policy 2012).

60 The Leahy-Smith America Invents Act of 2011 changed the rules slightly (Pub. L. No. 112–29; 35 U.S.C. §104).

61 The grace periods that do exist in different jurisdictions, such as in Canada and Japan, are for different things. The standards for what constitutes “publication” also vary widely. But the general point, that immediate online publication of sequence data endangered commercialization outside the US, holds. See *Patents Throughout the World* (Teegarden 2015).

62 At the time, the Max-Planck Society received government funding but was legally independent. The DFG received half state and half federal funds, and was the largest basic research patron for the German universities. The BMBF supported projects at universities and other institutes (*Mapping Our Genes*, 143–144).

63 Legally, these grants were administered through the Projektträger im DLR, a project management agency working on behalf of the BMBF and within the Helmholtz Research Center DLR (Deutsches Zentrum für Luft- und Raumfahrt, or the German Aerospace Centre) (BMBF to KMJ 2012 and 2013). In 1998, follow-up applications for the DNA Sequencing Consortium were successful, and the organization’s work continued through 2004.

64 There was also a second DHGP database, run by Annemarie Poustka at the German Cancer Research Center in Heidelberg (Unterhuber 1995). Presumably, this second resource was not involved in the privileged access arrangement that was negotiated with the Consortium.

65 To be fair, the arrangement had been in flux for years. German industry was initially expected to foot a full half of the DHGP bill (Unterhuber 1995).

66 In 1996, Sakaki had read a report on the Bermuda meeting from Naotake Ogasawara and immediately attempted to adhere to the Bermuda Principles (Sakaki interview 2011; Hattori to KMJ 2012). Francis Collins, however, remembers that only Sakaki was releasing his unfinished data in 1998 (Collins interview 2012).

67 As noted above, this contradicts Hattori’s recollections (Hattori to KMJ 2012).

68 Stephen Hilgartner dedicates an entire chapter to Celera, providing many more details about the lead-up to and fallout from the competition between the HGP and Celera and focusing on the press coverage (Hilgartner 2017, Chap. 7). Particularly useful are Hilgartner’s Figs. 7.1 and 7.2, which compare and contrast the two sides’ competing sequencing methodologies, and Tables 7.1 and 7.2, which document the public rhetoric. The science writer James Shreeve also chronicled the race in depth, documenting with aplomb the drama of this period (2004). It is not our intent to reproduce the complex narrative here; for the many twists and turns, readers should refer to Shreeve’s account.

69 *Haemophilus influenzae*, for Venter, had represented the finest way to do genomics: “We generated the data, we assembled it, we corrected all the errors, we interpreted it…. And that was our goal—not just to say, here is a sequence—but we’re the first ones on the planet to see the entire genome sequence of a species” (Venter interview 2012).

70 This shift in goals and standards, however, was made with some hesitancy on the parts of Collins, Olson, Waterston, and Sulston (Shreeve 2004, Chap. 14).

71 Shreeve’s statement was a reference to Francis Collins’s Christianity, which was widely recognized. Collins’s later book about genomics was entitled *The Language of God* (2007).

72 Interestingly, a second issue with data sharing had also plagued both TIGR’s relation to Human Genome Sciences and HGS’s agreement with SmithKline Beecham. Venter’s TIGR, a nonprofit laboratory, fought mightily and repeatedly with its for-profit unit, HGS, which did not adhere to TIGR’s preferred data-release policies. The company wanted to keep data private for at least a year, while Venter wanted TIGR’s ESTs shared at the time of publication. To make his case, Venter used the Merck Gene Index project “as a weapon,” arguing that since “similar sequences are out there … TIGR is free to go ahead and publish them” (Venter interview 2012). Venter, it appears, did not like either extreme. Ultimately, the issue led to the termination of his partnership with HGS, as Venter described in his post-hearing questions in 1998: on “June 20, 1997, The Institute for Genomic Research (TIGR) and Human Genome Science[s] (HGS) ended a collaborative arrangement that required TIGR to forego payments totalling [sp.] $38 million. The primary reason for my choosing to end this relationship and access to significant financial resources was a philosophical disagreement about the public release of DNA sequence data. The day after this relationship was terminated, TIGR made the largest deposit of DNA sequence data into the public domain in history” (*How Private Sector Developments Affect the Government Program*, 98).

73 Hilgartner (2017) links the necessity of *rapid* sharing to the project’s commitments, in the face of the Venter challenge, to a high quality public domain sequence. He does not, however, make the point that *daily* sharing was unnecessary, neither to “beat” Venter nor to accomplish these other goals. See, for instance, Hilgartner’s Table 7.2.

74 In the *Nature* paper, the “Genome Sequencing Centres” were “listed in order of total genomic sequence contributed, with a partial list of personnel” (Lander et al. 2001, p. 861). The Whitehead Institute was first, followed by the Sanger Centre—where Jane Rogers’s name appears first, followed by John Sulston’s—and the Washington University Genome Sequencing Center, led by Robert Waterston. The DOE’s JGI and the Baylor College of Medicine Human Genome Sequencing Center rounded out the top five laboratories.

75 As Shreeve elaborated: “Academics could download one million base pairs of DNA from the site per week simply by mouse-clicking their agreement not to redistribute the data for commercial purposes. If they wanted complete access to the genome, they had to submit a letter signed by an institution official agreeing to the same terms. The rules for private-sector users, however, were much more restrictive, and in effect prevented any commercial use without compensation to Celera” (2004, p. 361).

76 Hilgartner also notes the significance of backing by these powerful funders, but does not elaborate on the politics through which Sulston and Waterston gained favor for daily release (Hilgartner 2017, pp. 173–175).

77 CSHL has also developed a digital catalogue of international materials, alongside an interactive e-book for the US project (CSHL International History 2009; Davies 2016). Finally, the Bioethics Research Library at Georgetown University hosts a broad scope of primary source materials on the history of genetics and genomics, including interview transcripts and audio recordings from *The Gene Wars: Science, Politics, and the Human Genome* (1994; Cook-Deegan Archive).

78 These “principled” outcomes, downstream research and commercialization, could of course also be seen as “pragmatic,” promoting further scientific research and economic growth. The orientation of any such justification for public science, as we have shown, depends on context and framing. Several authors have argued how the HGP’s data-sharing policies were pragmatic and political (or, put differently, jointly scientific and symbolic, or scientific and political) (Hilgartner 1998, 2013, 2017; Stevens 2015c). It is a central tenet of the co-production framework, in fact, that these two categories, while they can be defined in various ways, are inextricably linked. Our story is an obvious affirmation of this concept.

79 Genomics education is the focus of the Bermuda Principles Foundation Fund, founded in Bermuda by Dr. Carika Weldon in 2016. In the spirit of public science for the public good, the fund facilitates programs designed to prepare Bermudian youth for careers in science and healthcare, and sponsors an annual, “Bermuda Principles Impact on [DNA] Splicing (BPIS)” conference (Bermuda Principles Foundation Fund 2017).

## List of Acronyms

ABI: Applied Biosystems
ACeDB: A C. elegans Data Base
AFM: Association Française contre les Myopathies (French Muscular Dystrophy Association)
BAC: Bacterial artificial chromosome
BBN: Bolt, Beranek, and Newman
BITNET: Because It’s There NETwork, or Because It’s Time NETwork
BMBF: Bundesministerium für Bildung und Forschung (German Federal Ministry of Education and Research)
cDNA: Complementary DNA
CEPH: Centre d’Etude du Polymorphisme Humain (Center for the Study of Human Polymorphisms, now Foundation Jean-Dausset-CEPH)
CNRS: Centre National de la Recherche Scientifique (French basic research agency)
Consed: Consensus editor
Contig: Contiguous DNA sequence
CSHL: Cold Spring Harbor Laboratory
DDBJ: DNA Databank of Japan
DEC: Declaration of Exceptional Circumstances
DFG: Deutsche Forschungsgemeinschaft (German basic research agency)
DHGP: German Human Genome Project
DLR: Deutsches Zentrum für Luft- und Raumfahrt (the German Aerospace Centre)
DNA: Deoxyribose nucleic acid
DOE: US Department of Energy
EBI: European Bioinformatics Institute
EC: European Community
ELSI: Ethical, Legal, and Social Implications (of genomics)
EMBL: European Molecular Biology Laboratory
ERDA: Energy Research and Development Administration
EST: Expressed sequence tag
FTP: File transfer protocol
FOSS: Free and Open Source Software
G5: The five largest HGP sequencing centers after 1999 (the Sanger Centre, Washington University in St. Louis, the Broad Institute of Harvard and MIT, the DOE Joint Genome Institute, and the Baylor College of Medicine)
GBF: German Research Centre for Biotechnology (in Braunschweig)
GDB: Genome Database (at Johns Hopkins University)
GESTEC: NIH Genome Science and Technology centers
HGI: Human Genome Initiative (of the US DOE)
HGMP: Human Genome Mapping Programme (of the UK MRC)
HGMW: Human Genome Mapping Workshop
HGP: Human Genome Project
HGS: Human Genome Sciences
HHMI: Howard Hughes Medical Institute
HTML: HyperText Markup Language
HLA: Human Leukocyte Antigen
HUGO: Human Genome Organization
ICRF: Imperial Cancer Research Fund (in London)
IHGSC: International Human Genome Sequencing Consortium
IMB: Institute of Molecular Biotechnology (in Jena)
JGI: Joint Genome Institute (of the DOE)
JST: Japan Science and Technology Agency (also STA)
KB: Kilobase (or Kb, kb)
LANL: Los Alamos National Laboratory
LLNL: Lawrence Livermore National Laboratory
LMB: Laboratory of Molecular Biology (University of Cambridge)
MB: Megabase (or Mb, mb)
MBL: Marine Biological Laboratory (in Woods Hole, MA)
MIM: Mendelian Inheritance in Man
MIT: Massachusetts Institute of Technology
MPI: Max Planck Institut für Molekulare Genetik (in Berlin)
MRC: UK Medical Research Council
NCBI: US National Center for Biotechnology Information
NCHGR: US National Center for Human Genome Research
NCI: US National Cancer Institute
NHGRI: US National Human Genome Research Institute
NIH: US National Institutes of Health
NINDS: US National Institute of Neurological Disorders and Stroke
NLM: US National Library of Medicine
NRC: US National Research Council (of the National Academy of Sciences)
NSF: US National Science Foundation
OBER: Office of Biological and Environmental Research (of the DOE)
OHER: Office of Health and Environmental Research (of the DOE)
OMIM: Online Mendelian Inheritance in Man
ORNL: Oak Ridge National Laboratory
OSRD: Office of Scientific Research and Development
OTA: Office of Technology Assessment (of the US Congress)
PCR: Polymerase chain reaction
PDB: Protein Data Bank (at the Brookhaven National Laboratory)
Phrap: Phil’s revised assembly program
Phred: Phil’s revised editor
PKD: Polycystic kidney disease
rDNA: Recombinant DNA
RIKEN: Japanese Institute of Physical and Chemical Research
RNA: Ribonucleic acid
RZPD: German genomic Resource Center (at the MPI in Berlin)
SAIL: Stanford Artificial Intelligence Laboratory
SCW: Single Chromosome Workshop
SNP: Single nucleotide polymorphism
STA: Japan Science and Technology Agency (also JST)
STS: Science and technology studies OR sequence-tagged site (meaning apparent with context)
TIGR: The Institute for Genomic Research
UCSF: University of California at San Francisco
UJAD: Unpublished in Journals and Available in Databases (Stephen Hilgartner’s term)
UNESCO: The United Nations Educational, Scientific, and Cultural Organization
USPTO: United States Patent and Trademark Office
USSR: Union of Soviet Socialist Republics
WCS: Worm Community System
YAC: Yeast artificial chromosome
